# Comparative morphological and molecular analysis confirms the presence of the West Nile virus mosquito vector, *Culex univittatus*, in the Iberian Peninsula

**DOI:** 10.1186/s13071-016-1877-7

**Published:** 2016-11-25

**Authors:** Verónica Mixão, Daniel Bravo Barriga, Ricardo Parreira, Maria Teresa Novo, Carla Alexandra Sousa, Eva Frontera, Marietjie Venter, Leo Braack, António Paulo Gouveia Almeida

**Affiliations:** 1 Global Health and Tropical Medicine, GHTM, UEI Medical Parasitology, Instituto de Higiene e Medicina Tropical, IHMT, Universidade Nova de Lisboa, UNL, Lisbon, Portugal; 2 Parasitology and Parasitic Diseases, Animal Health Department, Veterinary Faculty, University of Extremadura, Caceres, Spain; 3 Global Health and Tropical Medicine, GHTM, UEI Medical Microbiology, Instituto de Higiene e Medicina Tropical, IHMT, Universidade Nova de Lisboa, UNL, Lisbon, Portugal; 4 Centre for Viral Zoonoses, Department of Medical Virology, Faculty of Health Sciences, University of Pretoria, Pretoria, South Africa

**Keywords:** *Culex perexiguus*, *Culex univittatus*, Portugal, South Africa, Spain

## Abstract

**Background:**

*Culex univittatus* and *Culex perexiguus* mosquitoes (Diptera: Culicidae) are competent arbovirus vectors, but with unclear morphological differentiation. In Europe, and in the Iberian Peninsula in particular, the presence of either or both species is controversial. However, in order to conduct adequate surveillance for arboviruses in this region, it is crucial to clarify whether *Cx. univittatus* is present or not, as well as to critically assess existing differentiation tools. This study aimed to clarify this situation, by morphological and molecular phylogenetic comparison of Iberian specimens deemed as *Cx. univittatus*, with others of South African origin, i.e. from the type-locality region.

**Methods:**

Thus, morphological characteristics useful to distinguish both species, such as midfemur pale line, hindfemur R ratio, seta ***g*** R_1_ ratio, seta ***f*** shape, length of ventral arm of phalosome and number of setae on IX tergal abdominal segment, were observed. A phylogenetic analysis based on *cox*1 mtDNA, of which there were no sequences from *Cx. univittatus* yet available in the GenBank database, was performed.

**Results:**

This analysis showed that Iberian and South African specimens are morphologically similar, except for the length of the ventral arm of the phalosome, which was higher in the Iberian specimens. Although the Iberian specimens could not be accurately identified using BOLD Systems, phylogenetic analysis still grouped these closer to South African *Cx. univittatus*, than to *Cx. perexiguus* from Turkey and Pakistan, despite the observed segregation of both taxa as two individual monophyletic clusters with shared common ancestry.

**Conclusions:**

This survey demonstrates that the West Nile virus vector *Cx. univittatus* is present in the Iberian Peninsula.

**Electronic supplementary material:**

The online version of this article (doi:10.1186/s13071-016-1877-7) contains supplementary material, which is available to authorized users.

## Background

Mosquitoes are responsible for the transmission of several pathogens causing diseases with high morbidity and/or mortality [1]. Among them, the genus *Culex* comprises about 768 taxa, including some of the most ubiquitous, as well as important vectors of human pathogens which, in the present context of global warming and environmental changes, pose particular concern [1, 2]. Within the subgenus *Culex* lies the Univittatus subgroup, with four closely related taxa that exhibit external morphological similarities in all life stages [2, 3]: *Culex* (*Culex*) *univittatus* Theobald, 1901, *Culex* (*Culex*) *perexiguus* Theobald, 1903, *Culex* (*Culex*) *neavei* Theobald, 1906 and *Culex* (*Culex*) *fuscocephala* Theobald, 1907, the latter being an Oriental species [2]. *Culex univittatus* is a competent vector of arboviruses with public health importance, such as West Nile, Sindbis and Usutu viruses, in South Africa [4]. *Culex perexiguus* has also been found infected with West Nile, Sindbis and/or Usutu viruses, in Israel, Egypt and Saudi Arabia (reviewed in [3]). *Culex univittatus*/*perexiguus* was found as competent vector for West Nile in Portugal, Italy and Spain [5–7]. *Culex neavei* from South Africa seems to be a less competent vector of both West Nile and Sindbis viruses [8]. The methods of mosquito species identification in these viral surveys were not always stated, as referred below. However, this group has been subjected to extensive systematic treatment, with some taxa sunk under synonymy, or considered forms or varieties, until finally *Cx. perexiguus* was reinstated to full species rank [3, 9], as was *Cx. neavei* [8–10]. Thus, White [9] proposed a differentiation key that, based on the morphological studies by Jupp [10], indicates that analysis of morphological characters such as mid and hind femurs, and male genitalia, would allow the separation of *Cx. univittatus*, *Cx. perexiguus* and *Cx. neavei.* The characters used to distinguish *Cx. univittatus* and *Cx. perexiguus* are summarized in Additional file 1.

*Culex univittatus*, originally described from Salisbury, Zimbabwe (lectotype designated by White [9]), is widely distributed in the temperate highlands of the Afrotropical region, particularly in southern and eastern Africa, in countries such as Angola [11], Ethiopia, Kenya, Zimbabwe, South Africa and Madagascar [9, 10, 12], and Yemen, in the south-western corner of the Arabian Peninsula [3]. However, the occurrence of this species in the lowlands of western Africa, in countries such as Benin, Niger and Burkina Faso [9, 10, 12] has been considered controversial [3]. *Culex perexiguus* extends throughout the arid areas of West, North and East Africa, across the Sudan savannah belt, Mediterranean basin, Middle East, and south-western Asia, extending eastwards into India [3, 9, 13]. The distribution of *Culex neavei* is also somewhat controversial, occurring throughout the subtropical and tropical lowlands, either just in southern Africa, Reunion and Madagascar [9], or south of the Sahara [3].

The presence of *Cx. univittatus* in Europe has been the subject of controversy. It was reported for the first time in Portugal, by Ribeiro et al. [14], and in Spain, by Encinas-Grandes [15]. These reports included a thorough morphological analysis of adults of both sexes as well as larvae, and a sound systematic discussion. However, after examination of specimens from southern Europe (Italy and Greece) and Middle-East (Turkey), based on characteristics of the male genitalia and larvae, Harbach [13] found that these appeared to be *Cx. perexiguus,* and concluded that the species within the Univittatus subgroup that “occurs in southern Europe should be regarded as *Cx. perexiguus* rather than *Cx. univittatus*”. Recent molecular studies based on the analysis of mitochondrial *cox*1 gene confirmed the presence of *Cx. perexiguus* in Turkey [16].

Later surveys, usually focused on arboviruses, carried out in the Iberian Peninsula have recorded *Cx. univittatus* both in Portugal and Spain [17–21], by general external morphological identification, based on the findings of either Ribeiro et al. [14] and Encinas-Grandes [15], but without confirmation by the study of male genitalia. Likewise, *Cx. perexiguus* has also been recorded in Portugal [22] and in Spain, [23–27], albeit without any mention of particular morphological analysis or how the material was identified, and often exclusively based on the distribution criteria described by Harbach [3, 13]. Nevertheless, other authors [28–30] identified *Cx. perexiguus* by studying male genitalia, confirming its presence in Spain. The paucity of molecular data concerning these two taxa is also striking. The Barcode of Life Data Systems database (BOLD) [31] does not bear public sequences of either taxon, originating from Europe. Furthermore, the absence of *cox*1 sequences of *Cx. univittatus* in the GenBank database is notable, with only 8 sequences of *Cx. perexiguus* from Turkey and Pakistan.

*Culex univittatus* and *Cx. perexiguus* are considered mainly ornitophilic, although *Cx. univittatus* feeds also on humans, and more frequently than *Cx. perexiguus*, thus presenting a higher potential for arboviruses transmission between birds and humans or other mammals [3]. Furthermore, they also present different breeding place preferences, with *Cx. univittatus* immature stages found only in freshwater natural biotopes, while *Cx. perexiguus* tolerates moderate pollution or salinity and also use artificial containers [3]. In the Iberian Peninsula, *Cx. univittatus* is deemed as mainly ornithophilic, but also mammo- and particularly anthropophilic [14, 15]. However, *Cx. perexiguus* in Spain seems to be primarily ornithophilic and less mammophilic [26, 28] or precisely the opposite [27]. Thus, the body of evidence for each of these species vector competence, bionomic features, vectorial capacity and transmission efficiency, is still lacking particularly in this geographic region. Due to these differences and to the presence of arboviruses with medical importance such as West Nile and/or Usutu in Portugal and Spain [5, 7], the clarification of whether only *Cx. univittatus* or *Cx. perexiguus* or both species are present in the Iberian Peninsula, is imperative for the operation of surveillance programmes in this region. These programmes must accurately identify the presence and relative abundance of every vector species.

Therefore, the purpose of this study was to simultaneously use *cox*1 mtDNA as a molecular marker, coupled with a morphological analysis, including that of male genitalia, to identify specimens of Univittatus subgroup in the Iberian Peninsula, in order to ascertain which of its species is/are present in the extreme of western Europe, contributing to the clarification of earlier conflicting results. In parallel, these specimens were compared with specimens from the highlands of South Africa, also known as the Highveld, where *Cx. univittatus* is the only species of this subgroup known to occur. To the best of our knowledge, the studies that involved the analysis of mosquitoes collected in the Iberian Peninsula have neither used molecular data to corroborate the identification of these two species, nor combined this approach with morphological analysis.

## Methods

### Mosquito selection

**Fig. 1:**
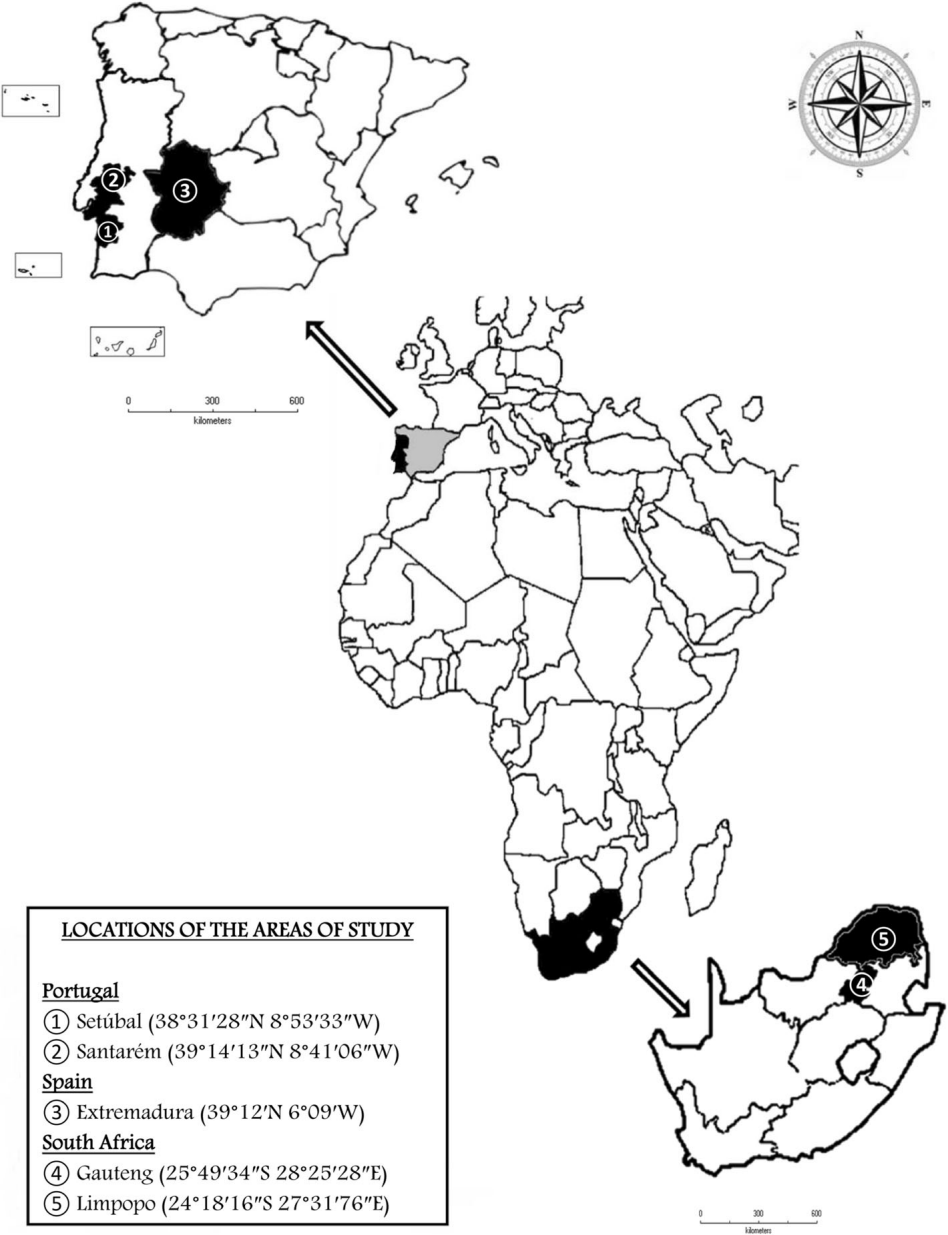
Map with the three main areas of study, Portugal, Spain and South Africa

Mosquitoes (*n* = 80) tentatively identified as *Cx. univittatus*, were collected in Portugal (*n* = 47) (districts of Santarém and Setúbal), between 2010 and 2013; in Spain (*n* = 15) (Extremadura Region) between 2012 and 2013; and in South Africa (*n* = 18) (Gauteng and Limpopo Provinces) in 2014 (Fig. 1), either by CDC miniature light-traps, mechanical hand aspirators (indoor resting mosquitoes), or tent traps (Additional file 2). Captured specimens were initially stored at -20 °C, brought to the respective laboratories and observed under a stereomicroscope and morphologically identified according to keys of Ribeiro & Ramos [32] for Portugal, of Becker et al. [1] for Spain, and of Jupp [33] for South Africa. While females were stored again at -20 °C until DNA extraction to be used for pathogen screening, males (*n* = 22) were kept in silica gel (Additional file 2).

### Morphology study and data analysis

Specimens were observed according to the keys of White [9] for the Univittatus subgroup. However, as many of these mosquitoes had already been used for pathogen screening, or were physically damaged, morphological characterization could only be done in a subsample of all the collected specimens. Critical to observe, included the presence or absence of a pale stripe in mid femur and determination of hind femoral index (R) (percentage of its length taken by the dorso-anterior black stripe) [10].

Male terminalia were sectioned from the abdomen and immersed in Marc André solution [34], for 5 days at room temperature. When clarified, genitalia were dissected under a stereomicroscope, in solidifiable formic acid-PVA mounting medium [34], and mounted between slide and cover slip. Gonocoxites were separated and, in each one, two structures were observed in its subapical lobe: (i) seta ***f***, whose tip was denoted as either thin/unswollen (whether rounded or pointed), or wide/swollen (usually rhomboid and where the tip was much wider than its “neck”, *c.* ≥ 2.5× its “neck”) [10]; (ii) seta ***g***, also known as “the leaflet”, for which R_1_ index was calculated, as the ratio of the greatest width (s) to the length (l), expressed as a percentage (s/l*100) [10].

In the phallosome, the length of the ventral arm (VA) (also known as *outer division* or *spine*), as well as the width of the lateral plate (LP) (also known as *aedeagal* plate), at the point of attachment of the former, were measured. Based on the fact that *Cx. univittatus* has a long spine-like VA, reaching beyond the caudal margin of LP [3], a ratio was calculated, consisting of VA/LP. The ninth abdominal segment was also dissected, mounted and the number of setae on its dorsal or tergal side, recorded.

Slide mounts were observed with Nomarski differential interference contrast under an Olympus microscope (BX51), and photographed with an Olympus SC30 digital camera. Normality of data distribution was assessed with Kolmogorov-Smirnov, and Shapiro-Wilk tests, and homogeneity of variances with Levene’s test. Student’s *t*-test and Mann-Whitney *U*-test, were used to compare means or medians, respectively, whether the data had normal distribution and homogeneity of variances, or not, respectively. Fisher’s exact test compared discontinuous or ordinal variables, such as the frequency of specimens with seta ***f***, whose tip was denoted as either thin/unswollen or wide/swollen. The statistical package SPSS 20.0 [35] was used. Beeswarm graphs (one-dimensional scatter) of seta ***g*** R_1_ ratio and VA/LP ratio, from both groups of specimens were plotted using the Beeswarm R package (version 0.2.3) to the R statistical software (version 3.2.4) [36].

### DNA extraction and amplification of *cox*1 mtDNA

Genomic DNA was extracted using the CTAB (Cetyltrimethylammonium bromide) method, as described by Ferreira et al. [20]. Phenol/chloroform/isoamyl alcohol was used for DNA purification. DNA was ethanol precipitated and suspended in TE buffer (pH 7.0) and stored at -20 °C until use. Negative controls were performed for each extraction procedure.

Amplification of *cox*1 mtDNA from both male and female specimens was performed using LCO1490 and HCO2198 specific primers, described by Folmer et al. [37]. PCR was performed in 20 μl reaction mixture containing GreenGoTaq® Flexi Buffer (Promega), 5 mM of MgCl_2_ (Promega), 0.2 mM of each dNTP (Promega), 0.3 pM of each primer, 0.04 U/μl of GoTaq® DNA Polymerase (Promega) and 1 ng/μl of template DNA. The thermal cycler was set at 95 °C for 5 min, followed by 40 cycles of denaturation for 30 s at 95 °C, annealing for 30 s at 48 °C, extension for 45 s at 72 °C, and a final extension for 5 min at 72 °C. The amplified products of approximately 650 bp were analysed by electrophoresis in 1.5% agarose gels stained with Ethidium bromide and observed under UV light.

### DNA sequencing and sequence analysis

PCR products amplified from each sample were purified with the QIAquick PCR Purification Kit (Qiagen GmbH, Hilden, Germany) and sequenced by GATC Biotech AG or STAB VIDA in forward and reverse senses, using the same primers as for the PCR. Sequences were edited in Chromas Lite 2.1.1 (Technelysium Pty Ltd) and consensus sequences for each forward/reverse pair were created in BioEdit [38], using CLUSTAL-W version 2.0 [39]. The identity at the species level was investigated based on the analysis of the generated *cox*1 sequences, taking into consideration both the higher similarity in the BOLD Systems identification tool (http://www.boldsystems.org/index.php/IDS_OpenIdEngine), and results of homology searches using the sequences deposited at GenBank (http://www.ncbi.nlm.nih.gov/genbank/). All newly-generated sequences were submitted to DNA Data Bank of Japan (DDBJ) database (http://www.ddbj.nig.ac.jp) under accession numbers LC088986–LC088999, LC100115, LC102118–LC102131, LC102134–LC102136, and LC102138–LC102162.

In order to better characterize our sequence dataset using phylogenetic analysis, *cox*1 mtDNA sequences from *Culex* mosquitoes were retrieved from GenBank (accession numbers, origin and other information about those sequences in Additional file 3). All sequences were aligned using the online version of MAFFT (http://mafft.cbrc.jp/alignment/server/index.html), with the G-INS-i interactive refinement method, and taking into account alignment. Confidence score was inferred with Guidance2 Server (http://guidance.tau.ac.il/ver2/). Only regions or sequences with a score higher than 90% were considered to posterior analysis. A region of 637 bp common to all sequences (as well as a smaller 287 bp internal fragment of the latter) were used for further analysis. MEGA 6 software [40] was used to identify variable sites in the alignment.

### Phylogenetic analysis

MEGA 6 software [40] was used to infer the best DNA substitution model for phylogenetic analysis. Maximum Likelihood trees were produced based on the Tamura 3-parameter formula [41], with heuristic searches based on initial trees obtained automatically from Neighbor-Joining to a matrix of pairwise distances estimated using the Maximum Composite Likelihood approach. Bootstrap coefficients were calculated for 10,000 replicates. Estimates of evolutionary pairwise divergence between all sequences, between and within the defined groups, were estimated using the Tamura 3-parameter model [41].

Phylogenetic reconstruction (consensus tree) following a Bayesian approach was also conducted using MrBayes v3.0b4 [42], using the GTR + Γ + I model (GTR-General Time Reversal; Γ-Gamma distribution; I-proportion of invariable sites) and default priors. This analysis consisted of 5 × 10^7^ generations starting from a random tree and four Markov chains with default heating values, sampled every 100th generation. Two separate runs were conducted for each analysis, and the first 10% sampled trees discarded as 'burn-in’. Maximum Clade Credibility trees were constructed using BEASTv1.7.5 [43], using the GTR + Γ + I model, and as coalescent priors a constant population size and a strict molecular clock. These analyses were run for 1 × 10^8^ generations starting from a random tree with sampling at every 5,000th generation. The results of two separate runs were combined using LogCombiner (available at http://beast.bio.ed.ac.uk/logcombiner), and the first 10% discarded as 'burn-in'. For each case, convergence was monitored with Tracer v1.6 (available from http://beast.bio.ed.ac.uk/tracer), ensuring that ESS values were above 200. The obtained phylogenetic trees were manipulated for display using FigTree v.1.4.2. (available at http://tree.bio.ed.ac.uk/software/figtree/).

## Results

### Morphological study

All specimens in this study had been tentatively morphologically identified as *Cx. univittatus* according the keys of White [9] and Jupp [10]. Curiously, upon observation of 49 specimens, 62.5% (20/32) from Portugal and Spain (PT & SP), and 58.8% (10/17) from South Africa (SA) displayed a clear continuous pale stripe in mid femur, while 21.9% (7/32) PT & SP, and 35.3% (6/17) from SA had an interrupted but clear line, while 15.6% (5/32) from PT & SP, and 5.9% (1/17) from SA had no line at all (Additional file 2). The presence and form of this character was therefore not significantly different between these two samples, Fisher’s exact test = 1.52, *P* = 0.414, two-sided exact significance.

As to the R hind-femoral index (*n* = 31), specimens from PT & SP had a mean of 82.7% (95% CI: 77.8–87.6, range 60–98, *n* = 16), and specimens from SA had a mean of 81.96% (95% CI: 76.7–87.2, range 66–95, *n* = 15), hence not significantly different (Student’s *t* = 0.22, *df* = 29, *P* = 0.83, 95% CI for the difference: -6.15–7.61).

**Fig. 2:**
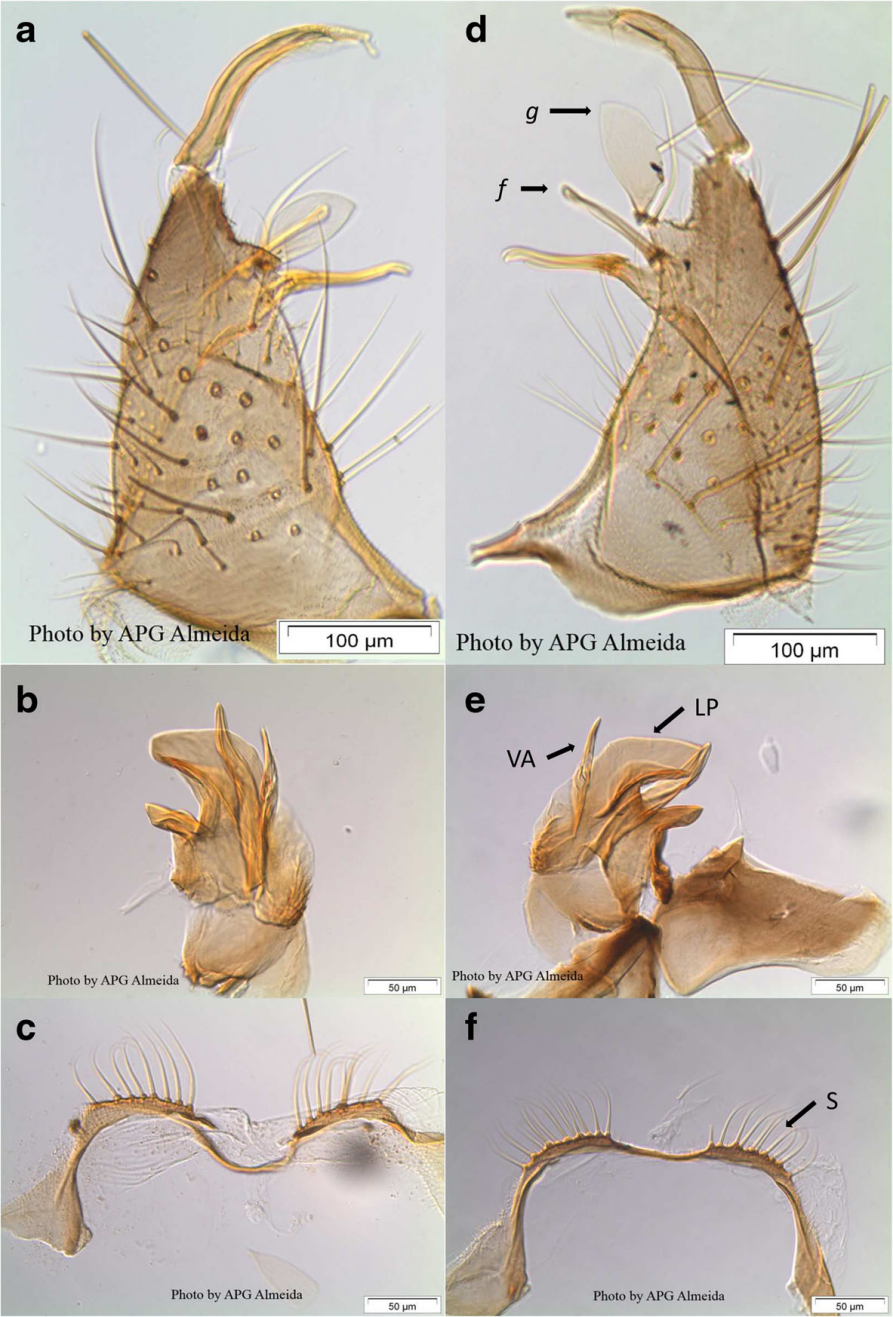
Dissected and mounted genitalia of male *Cx. univittatus* from South Africa (**a**-**c**; specimen SAfr-GAU-117-w3) and *Cx. univittatus* from Portugal (**d-f**; specimen Port-2630.69/3435). Gonocoxites with ***g*** and ***f*** setae (**a**, **d**; magnification 100×), phalosome (**b**, **e**; magnification 200×) with ventral arm (VA) and lateral arm (LA), and ninth segment tergum (**c**, **f**; magnification 200×) with setae (**S**). *Scale-bars*: **a**, **d**, 100 μm; **b**, **c**, **e**, **f**, 50 μm

Dissected genitalia from 22 males, 11 from Portugal and 11 from South Africa (the sample from Spain did not include males), were analysed (Fig. 2; full collection of photographs in Additional file 4). The tip of seta *f* was thin in 89.5% and swollen in 10.5% of Portuguese specimens *versus* thin in 75% and swollen in 25% of South African specimens, hence not significantly different, Fisher’s exact test 2-sided *P* = 0.407, *n* = 39 (see Additional file 5 for all data).

**Fig. 3:**
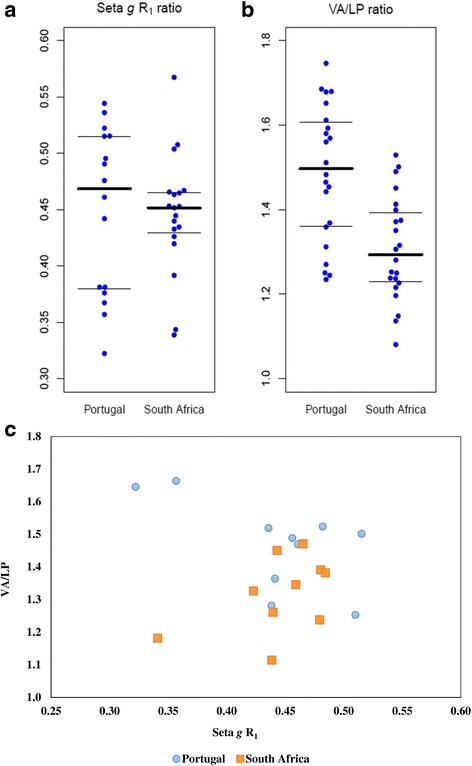
Beeswarm scatter plots (**a**, **b**), and Scatter diagram (**c**). Gonocoxite’s leaflet or seta ***g*** R_1_ ratio [10] (**a**) and phallosome’s ratio of the length of the ventral arm (VA) over the width of the lateral plate (LP), at the point of attachment of the former, VA/LP, from Portuguese and South African mosquitoes (**b**). All ratios for individual structures are plotted. The horizontal lines show the 25, 50 and 75% quartiles. Scatter diagram of ***g*** R_1_ ratio *versus* VA/LP ratio of mean values for each specimen (**c**)

Leaflet or seta ***g*** R_1_ ratio [10] varied between 32 and 54%, mean 45% (± 0.07 SD), for Portuguese specimens, and 34–57%, mean 45% (± 0.05 SD), for South African specimens (Fig. 3a), hence not significantly different between these two populations; Student’s *t* = 0.15, *df* = 33, *P* = 0.8 (see Additional file 5 for all data).

For the phallosome, the ratio of the length of the ventral arm (VA) over the width of the lateral plate (LP) at the point of attachment of the former, VA/LP, varied between 1.235–1.746, mean 1.451 (± 0.17 standard deviation, SD) for Portuguese specimens, and from 1.081–1.529, mean 1.321 (± 0.13 SD) for the South African specimens (Fig. 3b). This difference was shown to be statistically significant by Student’s *t-*test [*t* = 4.18, *df* = 42, *P* < 0.001, 95% CI of the difference: 0.09–0.27 (data with normal distribution and homogeneity of variances)] (see Additional file 5 for all data). A scatter diagram of paired values of seta ***g*** R_1_ index and VA/LP ratio is plotted in Fig. 3c, showing an overlap of these compound ratios for the two population samples.

The ninth tergal lobe had 8–11 (median 9) setae for Portuguese specimens, and 7–15 (median 10) setae for South African specimens (Fig. 2). This difference, however, was not statistically significant, using the Mann-Whitney *U*-test (U = 262, *P* = 0.286) (see Additional file 5 for all data).

### *cox*1 mtDNA amplification and sequence analysis

From all 80 specimens, it was only possible to have good *cox*1 mtDNA amplification for 56 samples (Additional file 2). From these specimens, consensus sequences were obtained and examined in BLASTn and BOLD System. Their analysis with BLASTn revealed homologies ranging between 90 and 96% with *cox*1 sequences of *Cx. perexiguus* (accession numbers: KJ012105.1, KF406802.1, KJ012109.1), worthy of being noted the absence of *cox*1 sequences of *Cx. univittatus* in the GenBank database (the only sequence found for *Cx. univittatus* in GenBank was a 375 bp mtDNA sequence from 12 NADH dehydrogenase subunit 4 gene, accession number: EF030093.1). Therefore, BLASTn could not allow an accurate identification of our samples, as even with the MegaBlast option implemented, sequence homology values of 96% do not allow for clear-cut species identification.

When submitted to the BOLD System identification tool, all the sequences from South Africa presented more than 98.46% similarity with *Cx. univittatus* sequences, though for the great majority of them (all but 3), sequence similarity values with *Cx. univittatus* was equal or higher than 99.23%. Thus, all samples from South Africa were considered as *Cx. univittatus* by the BOLD system platform (Additional file 2). Sequences from the Iberian Peninsula, in BOLD System platform revealed similarities with *Cx. univittatus* that varied from 97.22 to 98.01%. After these higher similarities, in 21 samples, the next matches corresponded to sequences of *Cx. univittatus*, and then, to *Cx. perexiguus* (in this order) (Additional file 6). However, in 7 samples from Portugal, as well as in all samples from Spain, this order was inverted, with higher similarity corresponding to *Cx. univittatus*, immediately followed by similarities (< 97%) with *Cx. perexiguus* (Additional file 2), and thereafter to *Cx. univittatus* again. Therefore, it was not possible to clearly identify the Iberian specimens as *Cx. univittatus* or *Cx. perexiguus*. However, in phylogenetic trees obtained through BOLD Systems identification, our sequences appear clustering with *Cx. univittatus* sequences, and in a different clade of *Cx. perexiguus* sequences, including 4 sequences from Spain (Additional file 6).

### Phylogenetic analysis

Additionally, a 637 bp region of the alignment obtained was analysed, leading to the finding of 76 polymorphic sites in sequences from the Univittatus subgroup (Additional file 7). Those polymorphisms revealed the existence of substantial differences between sequences from the Iberian Peninsula (Portugal and Spain), with respect to other species from this subgroup, including *Cx. univittatus* from South Africa and *Cx. perexiguus* from Turkey and Pakistan. From all 56 DNA sequences, only 44 were considered for phylogenetic analysis, since all sequences with less than 648 bp were excluded. All the aligned positions with a Guidance score lower than 90% were also excluded (Additional file 2).

**Fig. 4:**
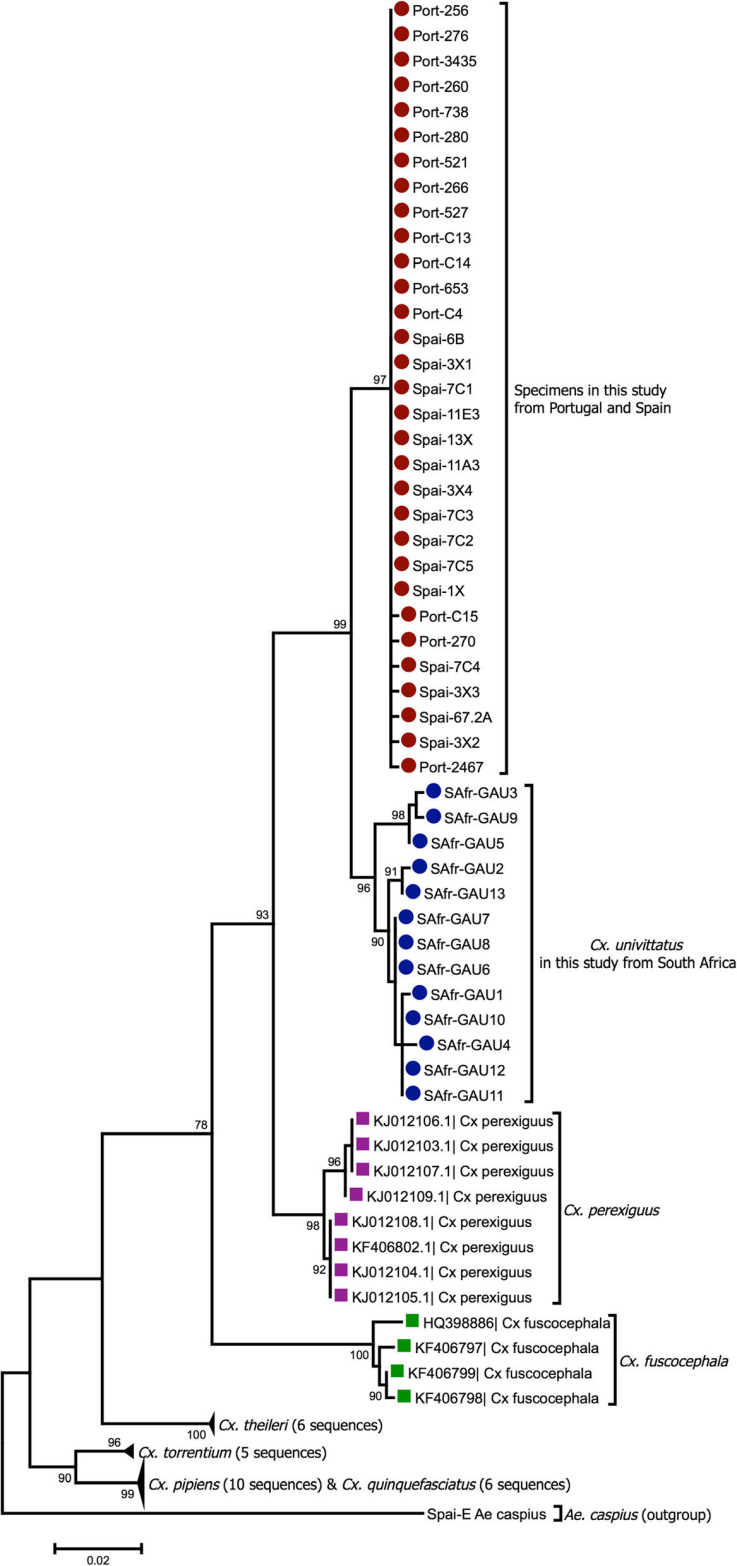
Phylogenetic analysis by Maximum Likelihood. The tree with the highest log likelihood (-2167.4061) is shown. The size bar indicates 0.02 substitutions per site. The analysis involved 84 nucleotide sequences, with a total of 637 positions in the final dataset. The tree has been rooted using *Ae.* (*Och*) *caspius* as the outgroup. *Abbreviations*: SAfr, South Africa; Port, Portugal; Spai, Spain

By phylogenetic analysis (Fig. 4) it was perceptible that sequences from Portugal and Spain are closer to each other, forming a joint clade that groups the Iberian specimens together (bootstrap value, BS = 97), which is a sister clade of another one which clustered the South African sequences, considered as *bona fide Cx. univittatus* (BS = 99). This monophyletic clade that reveals common ancestry for the Iberian and South African sequences is only subsequently joined with that defining *Cx. perexiguus* (BS = 93). Therefore, the analysis of *cox*1 coding sequences here performed strongly suggests that Portuguese and Spanish specimens are closer to the South African *Cx. univittatus,* than to *Cx. perexiguus* from Turkey and Pakistan (Fig. 4). Furthermore, phylogenetic analyses carried out under a Bayesian framework, as implemented in MrBayes (consensus tree with default priors) or Beast (strict molecular clock and a constant population size) confirmed the topology of the ML tree. Both trees present identical topologies, for which only the consensus tree is shown as Additional file 8.

Our phylogenetic analysis also provides a clear differentiation between all our sequences and those from other *Culex* taxa, namely *Cx. fuscocephala*, another member of the Univittatus subgroup. On the other hand, no sequences of *Cx. neavei* were included as none were available in GenBank. A second phylogenetic analysis, including sequences of lesser quality, and from a smaller part of the alignment (287 bp), but including more male specimens, in relation to Fig. 4, with combined morphological genitalia analysis, corroborated these findings, with only a slight decrease in bootstrap values and maintaining the same tree topology (Additional file 9).

The analysis of pairwise distance values also indicates that *cox*1 mtDNA sequences from Portugal and Spain are very similar to one another, some comparisons revealing sequence identity (genetic distance of 0), which is in accordance with results from determination of polymorphic sites presented in the previous section. Estimates of average evolutionary divergence over sequence pairs within groups (Additional file 10) allowed the confirmation of low divergences within samples from Portugal and Spain (0.001; standard error, SE 0.0), supporting our decision to perform the analysis grouping sequences from both countries. Estimates of evolutionary divergence over sequence pairs between groups showed that sequences from Portugal and Spain have a distance of 0.022 (SE 0.005) with sequences of *Cx. univittatus* from South Africa and of 0.042 (SE 0.008) with *Cx. perexiguus* from Turkey and Pakistan (Table 1).

**Table 1:** Estimates of evolutionary divergence of *cox*1 over sequence pairs between groups. The analysis involved 84 nucleotide sequences. Codon positions included were 1st + 2nd + 3rd + Noncoding. All positions containing gaps and missing data were eliminated. There were a total of 637 positions in the final dataset. Data are presented as the mean followed by standard error estimates in parentheses

		Portugal and Spain	1	2	3	4	5	6	7
1	*Cx. univittatus*	0.022 (0.005)							
2	*Cx. perexiguus*	0.042 (0.008)	0.044 (0.008)						
3	*Cx. fuscocephala*	0.077 (0.011)	0.079 (0.012)	0.067 (0.010)					
4	*Cx. pipiens*	0.084 (0.012)	0.087 (0.012)	0.087 (0.012)	0.076 (0.011)				
5	*Cx. quinquefasciatus*	0.085 (0.012)	0.087 (0.012)	0.089 (0.012)	0.078 (0.011)	0.002 (0.002)			
6	*Cx. torrentium*	0.085 (0.012)	0.088 (0.012)	0.088 (0.012)	0.077 (0.011)	0.028 (0.006)	0.031 (0.007)		
7	*Cx. theileri*	0.075 (0.011)	0.078 (0.011)	0.072 (0.011)	0.093 (0.012)	0.065 (0.011)	0.068 (0.011)	0.059 (0.010)	
8	*Ae. caspius* (outgroup)	0.148 (0.017)	0.138 (0.016)	0.140 (0.015)	0.142 (0.016)	0.126 (0.015)	0.125 (0.015)	0.125 (0.014)	0.134 (0.015)

## Discussion

The Univittatus subgroup of *Culex* spp. mosquitoes includes *Cx. univittatus* and *Cx. perexiguus*, which are vectors of arboviruses such as West Nile, Sindbis or Usutu. These viruses are responsible for various febrile or neurological syndromes, either in humans or other animals, in several countries of the northern and southern hemisphere especially in Europe and Africa [4–8]. However, controversy has existed regarding which species of this subgroup is/are present in Europe. In the Iberian Peninsula, the presence of *Cx. univittatus* has been documented [14, 15], while specimens from Italy, Greece and Turkey were stated to be *Cx. perexiguus* [13, 16]. In the context of arboviral circulation [5–7], the correct identification of infected/infectious species is mandatory since all cascade of bionomical and epidemiological studies, as well as control strategies that might be applied, depend on this knowledge.

In this study, comparative morphological and molecular analyses were carried out, based on the study of specimens putatively identified as *Cx. univittatus* from Portugal and Spain, as well as others previously identified as *Cx. univittatus*, from a region where this is the only species of the Univittatus subgroup known to be present (the Highveld region of South Africa) [10].

The morphological analysis revealed no significant differences in the proportion of mosquitoes from either population that possessed a continuous pale line on the mid femur. Although this character is one of those used to separate *Cx. univittatus* from both *Cx. perexiguus* and *Cx. neavei* [9], contrary to what might be expected, *Cx. univittatus* from the Highveld South Africa often have an interrupted discontinuous pale line, possibly indicating that this feature should not be considered a determinant or reliable character for the identification of this species. The hind femoral R index which allows the differentiation between *Cx. univittatus* and *Cx. neavei* [10], was not different between these mosquito populations of South Africa and Iberia Peninsula, either. Obviously, the observation of both these characters is strongly conditioned by the conservation status of the captured specimens, which in CDC traps is often poor, hence should be evaluated with caution.

In the males, the study of the genitalia, allowed us the confirmation that the shape of the tip of seta ***f***, the seta ***g*** R_1_ ratio, and the number of setae in the ninth tergal lobe, are also similar between the Portuguese and the South African specimens. In the phalosome, although the ventral arm seemed to be longer in Portuguese specimens than in South African ones, the range of variation was overlapping, and in any case would point further in the direction of *Cx. univittatus*, rather than to *Cx. perexiguus* which has a short ventral arm [3, 9]. Altogether, these morphological characters, particularly those from the genitalia, seem to indicate that the Iberian specimens should be identified as *Cx. univittatus*, confirming previous studies [14, 15].

Identification of our samples through the BOLD Systems platform clearly assigned the South African specimens as *Cx. univittatus*. Likewise, the sequences of Turkish *Cx. perexiguus* from GenBank [16] were clearly identified as *Cx. perexiguus*. For the Iberian specimens, no sequence was clearly identified as either species, although their higher similarity was always closer to *Cx. univittatus*. Furthermore, trees generated through the BOLD System identification tool showed a differentiation of the sequences of Portuguese and Spanish origin with respect to four sequences of *Cx. perexiguus* from Spain. Although the latter were not available for further sequence inspection, the separation between *Cx. univittatus* and *Cx. perexiguus* was further reinforced by phylogenetic analysis. However, in the Barcode of Life Data Systems database [31], 184 sequences of *Cx. perexiguus* were referred with origins such as Jordan, Tanzania, Turkey, Kenya, India and Pakistan, with no mention to the existence of those *Cx. perexiguus* sequences from Spain that appeared in the trees (http://www.boldsystems.org/index.php/Taxbrowser_Taxonpage?taxon=culex+perexiguus&searchTax= accessed on 28-6-2016).

Likewise, in BOLD Systems platform there are only 21 sequences of *Cx. univittatus* from Tanzania, 1 from South Africa and 1 from Kenya. Therefore it is not surprising that Iberian samples did not reach maximum similarity or clear identification (http://boldsystems.org/index.php/Taxbrowser_Taxonpage?taxon=culex+univittatus&searchTax=, accessed on the 28-06-2016). These results show one of the limitations of barcoding identification that is the dependence on available similar sequences for evaluation. Other authors have also reported some difficulties in distinguishing some mosquito close related species by barcoding [44, 45]. Thus, the phylogenetic analysis based on the larger region of *cox*1 mtDNA performed in this survey was essential to clarify the identification of Iberian specimens.

In order to perform a rigorous phylogenetic analysis (637 bp *cox*1 fragment), some of the specimens were excluded from the dataset due to a low score in the alignment, or because of their reduced size. This latter reason led to the absence from this so-called main-tree of some sequences obtained from male specimens with morphological analysis associated. For that reason, a second tree corresponding to the analysis of a smaller fragment size (287 bp) was generated. While the number of species differs in both datasets (e.g. *Cx. fuscocephala* is absent from one of them) the resulting sequence-clustering pattern (tree topology) remained generally congruent with that of the main-tree. Altogether, the phylogenetic analyses here presented allowed the distinction between all the known species within the Univittatus subgoup (*Cx. univittatus*, *Cx. perexiguus* and *Cx. fuscocephala*) except for *Cx. neavei*, for which *cox*1 sequences could not be found in the public databases. Fortunately, the BOLD Systems database does include *Cx. neavei* sequences (8 from Kenya and Nigeria, albeit not public), therefore their absence from the BOLD Systems automatic sequence identification result lists clearly shows that the specimens analysed in this report are not closely related to this species. We therefore consider their absence in our phylogenetic analysis not paramount to the study. The phylogenetic analysis hereby reported confirms that *cox*1 sequences amplified from mosquitoes from Portugal and Spain cluster together and are closer to *Cx. univittatus* from South Africa than to *Cx. perexiguus* from Turkey and Pakistan, in accordance with the BOLD system analysis.

Although the obtained Bayesian trees were based on a smaller sequence dataset than that previously used for the ML phylogenetic reconstruction, both of them showed a clear separation of the *Cx. perexiguus* and *Cx. univittatus* clusters, and a clear-cut segregation of the South African *Cx. univittatus* away from the Iberian *Cx. univittatus*/*perexiguus* sequences.

The differentiation observed between the Iberian and the South African cluster is in accordance with the genitalia VA/LP ratio values, which although overlapping, were already statistically different. Such differences are naturally expected in such geographically distant populations, and it would be interesting to study the crossbreeding success of these populations. Nevertheless, both the morphological and molecular analysis were coherent in supporting the identification of these Iberian specimens as *Cx. univittatus*.

## Conclusions

This study represents, to the best of our knowledge, the first molecular and phylogenetic analysis of *Cx. univittatus*/*perexiguus*. Although not exhaustive, either geographically or taxonomically, our results clearly confirm the presence of *Cx. univittatus* in the Iberian Peninsula, as previous morphological studies have shown [14, 15]. Thus, it can be concluded that *Cx. perexiguus* is not the only species of the Univittatus subgroup existing in Europe. It was also possible to see that these *Cx. univittatus* specimens are genetically different from those from South Africa, and it would be interesting to study the evolutionary track of the Univittatus subgroup, including its other members and other origins, to clarify their evolutionary relationships. Considering the vector role for arboviruses of both these taxa and the lack of updated characterization of their respective vectorial capacity and transmission efficiency, further bionomic characterization subsequent to correct species identification is needed for a sounder knowledge of arbovirus receptivity in this geographic region.

## Additional files

Additional file 1: Characters used to distinguish *Cx. univittatus* and *Cx. perexiguus.* (PDF 34 kb)
Additional file 2: Samples analysed with respective morphological and molecular data. (XLSX 226 kb)
Additional file 3:
*cox*1 mtDNA sequences retrieved from the GenBank database for sequence and phylogenetic analysis. (PDF 89 kb)
Additional file 4: Photos of male genitalia of *Cx. univittatus* from South Africa and Portuguese specimens. (PDF 3361 kb)
Additional file 5: Data for the structures of male genitalia results of statistical analyses. (XLSX 252 kb)
Additional file 6: Example of some results obtained in BOLD Systems identification tool. Percentage of similarity results and phylogenetic trees are shown. (PDF 10649 kb)
Additional file 7: Variable sites found in a 637 bp region of *cox*1 mtDNA alignment of Univittatus subgroup. *Abbreviations*: SAfr, *Culex univittatus* from South Africa; Port, specimens from Portugal; Spai, specimens from Spain. (PDF 86 kb)
Additional file 8: Bayesian phylogenetic analysis (consensus tree) based on *cox*1 mosquito sequences. At specific branch nodes posterior probabilities ≥ 0.90 are indicated. The scale-bar indicates the number of nucleotide substitutions per site. The tree was rooted with a *cox*1 sequence from *Ae.* (*Och.*) *caspius*. (TIF 2231 kb)
Additional file 9: Molecular phylogenetic analysis of a small fragment of the *cox*1 alignment, with a higher number of male sequences, by Maximum Likelihood. The tree with the highest log likelihood (-883.6486) is shown. The percentage of trees in which the associated taxa clustered together is shown next to the branches. The scale-bar indicates 0.02 substitutions per site. The analysis involved 90 nucleotide sequences. There were a total of 287 positions in the final dataset. (TIFF 240 kb)
Additional file 10: Estimates of average evolutionary divergence in *cox*1 over sequence pairs within groups. The numbers of base substitutions per site from averaging over all sequence pairs within each group are shown. Standard error estimates are shown in the last column. The analysis involved 84 nucleotide sequences. Codon positions included were 1st + 2nd + 3rd + Noncoding. All positions containing gaps and missing data were eliminated. There were a total of 637 positions in the final dataset. (PDF 59 kb)

## Abbreviations

*cox*1: Cytochrome *c* oxidade subunit 1 mtDNA
mtDNA: Mitochondrial DNA
USU: Usutu Virus
WNV: West Nile Virus
