# Supplementary material for: Comparative morphological and molecular analysis confirms the presence of the West Nile virus mosquito vector, Culex univittatus, in the Iberian Peninsula
Source: Parasit Vectors. 2016 Nov 25;9:601. doi: 10.1186/s13071-016-1877-7 (PMC5123335; doi:10.1186/s13071-016-1877-7)
Supplement: Additional file 1: — Characters used to distinguish Cx. univittatus and Cx. perexiguus. (PDF 34 kb) [file 13071_2016_1877_MOESM1_ESM.pdf]

Adicional file 1 - Morphological characters for distinction between *Culex univitattus* and *Cx. perexiguus*, adapted from Jupp (1971), White (1975), and Harbach (1985, 1988).

	Species	
**Character**	*Culex univittatus* Theobald, 1901	*Culex perexiguus* Theobald, 1903
I. Ventral surface of proboscis	• pale in middle	• pale except at base<br>• weakly pale on distal 0.25
II. Postspiracular area	• tendency for scales to cover less than dorsal 0.5	• tendency for scale to cover more than dorsal 0.5
III. Forefemur	• sometimes with indistinct anterior pale stripe	• usually with indistinct anterior pale stripe
IV. Midfemur	• with complete distinct or indistinct anterior pale stripe	• with or without incomplete faint or distinct anterior pale stripe
V. Hindfemur	proportion of the length of femur occupied by the dorso-anterior black stripe or "femoral index" (R)	
	• R ≥ 80%	• R ≤ 70%
VI. Hindtibia	• with distinct anterior and posterior pale stripes on proximal 0.8, separated ventrally by complete dark stripe;<br>• with distinct apical pale spot	• with distinct anterior and posterior pale stripes on proximal 0.8, partly separated on proximal 0.5 or less by weak ventral dark stripe;<br>• with distinct apical pale spot
VII. Wing		
a. Costa	• with short line of pale scales at base	• with short line of pale scales at base
b. Vein 2A	• female usually with line of scales	• female occasionally with few scales
VIII. Male genitalia		
a. Seta *g*	• narrow, "leaflet index" R1 = width/length*100 ≤ 50%	• broad, "leaflet index" R1 = width/length*100 ≥ 50%
b. Seta *f*	• tip narrow/unswollen	• tip narrow/unswollen
c. Ventral arm	• longer than width of lateral plate at point of attachment	• shorter than width of lateral plate at point of attachment
