# Supplementary material for: Comparative morphological and molecular analysis confirms the presence of the West Nile virus mosquito vector, Culex univittatus, in the Iberian Peninsula
Source: Parasit Vectors. 2016 Nov 25;9:601. doi: 10.1186/s13071-016-1877-7 (PMC5123335; doi:10.1186/s13071-016-1877-7)
Supplement: Additional file 3: — cox1 mtDNA sequences retrieved from the GenBank database for sequence and phylogenetic analysis. (PDF 89 kb) [file 13071_2016_1877_MOESM3_ESM.pdf]

Additional file 3: *cox*1 mtDNA sequences retrieved from the GenBank database for sequence and phylogenetic analysis

Sequence Code	Accesion Number	Fragment size (bp)	Species	Country
KJ012109.1| Cx perexiguus	KJ012109.1	658	*Cx. perexiguus*	Turkey
KJ012107.1| Cx perexiguus	KJ012107.1	658	*Cx. perexiguus*	Turkey
KJ012106.1| Cx perexiguus	KJ012106.1	658	*Cx. perexiguus*	Turkey
KJ012103.1| Cx perexiguus	KJ012103.1	658	*Cx. perexiguus*	Turkey
KJ012108.1| Cx perexiguus	KJ012108.1	658	*Cx. perexiguus*	Turkey
KF406802.1| Cx perexiguus	KF406802.1	658	*Cx. perexiguus*	Turkey
KJ012104.1| Cx perexiguus	KJ012104.1	658	*Cx. perexiguus*	Turkey
KJ012105.1| Cx perexiguus	KJ012105.1	658	*Cx. perexiguus*	Turkey
HQ398886| Cx fuscocephala	HQ398886	658	*Cx. fuscocephala*	Vietnam
KF406799| Cx fuscocephala	KF406799	658	*Cx. fuscocephala*	Pakistan
KF406798| Cx fuscocephala	KF406798	658	*Cx. fuscocephala*	Pakistan
KF406797| Cx fuscocephala	KF406797	658	*Cx. fuscocephala*	Pakistan
KJ012148.1| Cx pipiens	KJ012148.1	658	*Cx. pipiens*	Turkey
KJ012143.1| Cx pipiens	KJ012143.1	658	*Cx. pipiens*	Turkey
HG793546.1| Cx pipiens	HG793546.1	658	*Cx. pipiens*	Germany
HF562800.1| Cx pipiens	HF562800.1	658	*Cx. pipiens*	Germany
HF562725.1| Cx pipiens	HF562725.1	658	*Cx. pipiens*	Germany
KM258199.1| Cx pipiens	KM258199.1	658	*Cx. pipiens*	Belgium
KJ012161.1| Cx pipiens mol	KJ012161.1	658	*Cx. pipiens*	Turkey
JQ958372.1| Cx pipiens	JQ958372.1	709	*Cx. pipiens*	Tehran
HE997153.1| Cx pipiens	HE997153.1	673	*Cx. pipiens*	Germany
FN395179.1| Cx pipiens mol	FN395179.1	1542	*Cx. pipiens*	Europe
FN395204.1| Cx quinquefasciatus	FN395204.1	1542	*Cx. quinquefasciatus*	Pakistan
KJ012173.1| Cx quinquefasciatus	KJ012173.1	658	*Cx. quinquefasciatus*	Turkey
KC970297.1| Cx quinquefasciatus	KC970297.1	658	*Cx. quinquefasciatus*	India
KF407827.1| Cx quinquefasciatus	KF407827.1	658	*Cx. quinquefasciatus*	Pakistan
KF407713.1| Cx quinquefasciatus	KF407713.1	658	*Cx. quinquefasciatus*	Pakistan
KF407091.1| Cx quinquefasciatus	KF407091.1	658	*Cx. quinquefasciatus*	Pakistan
HE997083| Cx torrentium	HE997083	658	*Cx. torrentium*	Germany
HE997096| Cx torrentium	HE997096	658	*Cx. torrentium*	Germany
HE997101| Cx torrentium	HE997101	658	*Cx. torrentium*	Germany
HE997142| Cx torrentium	HE997142	658	*Cx. torrentium*	Germany
HG793632| Cx torrentium	HG793632	658	*Cx. torrentium*	Germany
KJ012204.1| Cx theileri	KJ012204.1	658	*Cx. theileri*	Turkey
FJ210899.1| Cx theileri	FJ210899.1	658	*Cx. theileri*	Iran
HE610460| Cx theileri	HE610460	688	*Cx. theileri*	Portugal
HE610457| Cx theileri	HE610457	650	*Cx. theileri*	Portugal
Port-3427 Cx theileri	LC102116	756	*Cx. theileri*	Portugal
Port-3430 Cx theileri	LC102115	740	*Cx. theileri*	Portugal
Spai-E_Ae caspius	LC090050*	648	*Ae. caspius*	Spain

* This sequence was used as outgroup.
