# Supplementary material for: Comparative morphological and molecular analysis confirms the presence of the West Nile virus mosquito vector, Culex univittatus, in the Iberian Peninsula
Source: Parasit Vectors. 2016 Nov 25;9:601. doi: 10.1186/s13071-016-1877-7 (PMC5123335; doi:10.1186/s13071-016-1877-7)

Culex (Culex) univittatus

From Portugal and South Africa, Gauteng Province.

Dissected genitalia microscope photos.

Port-2.1/C6R; Gonocoxite, X100

•

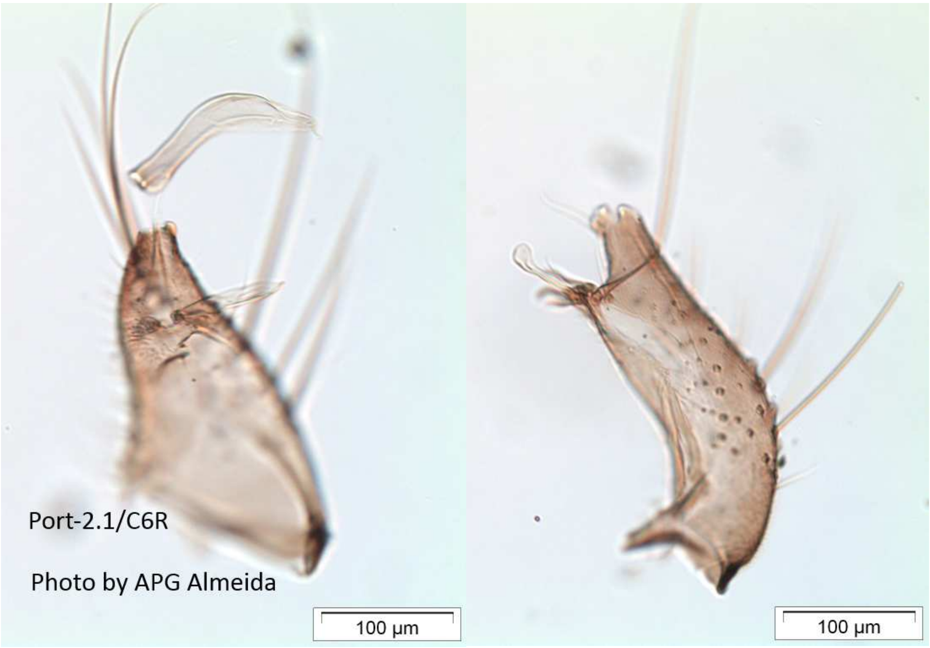

Port-2.1/C6R; Phalosome, X200

•

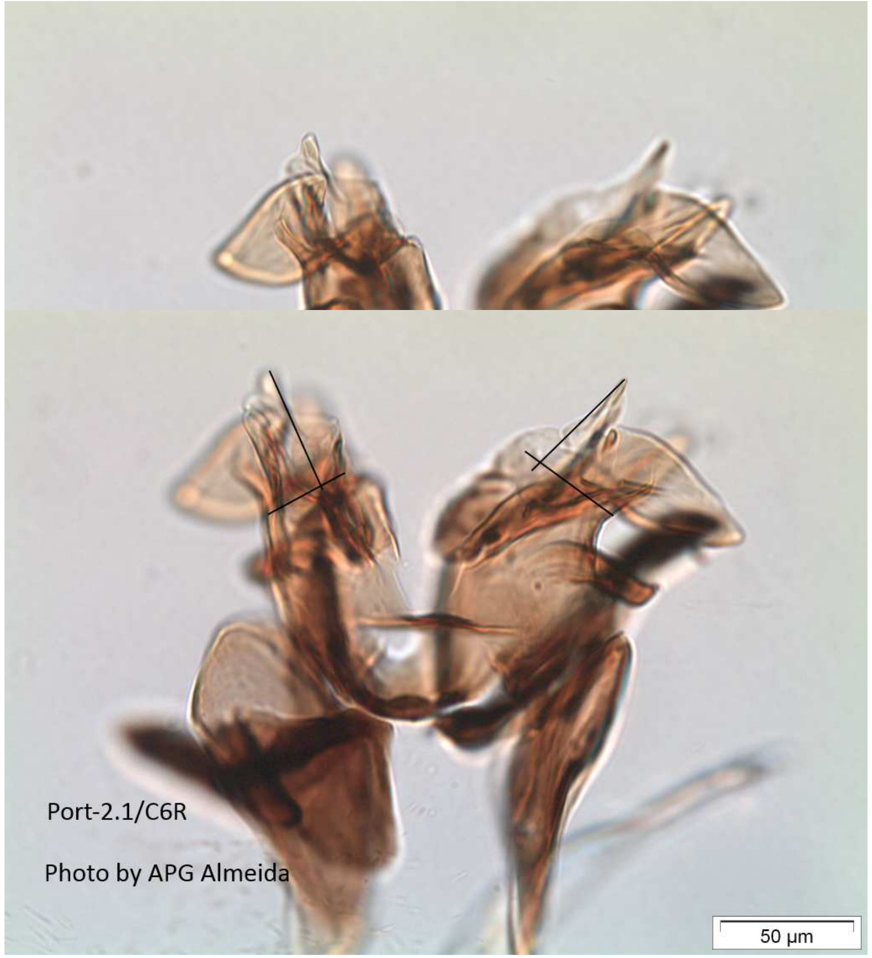

Port-2.1/C6R; Phalosome, X200

- (remounted)

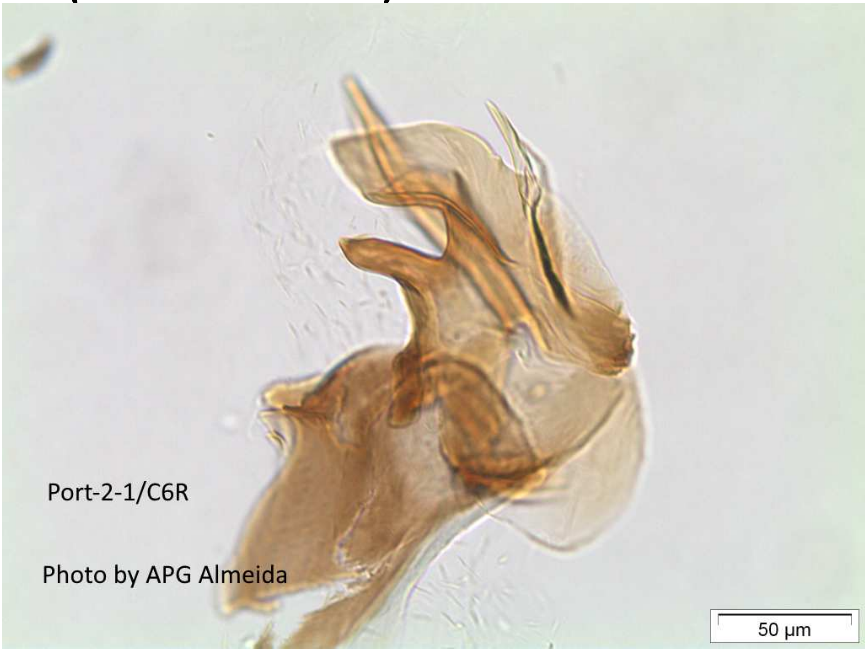

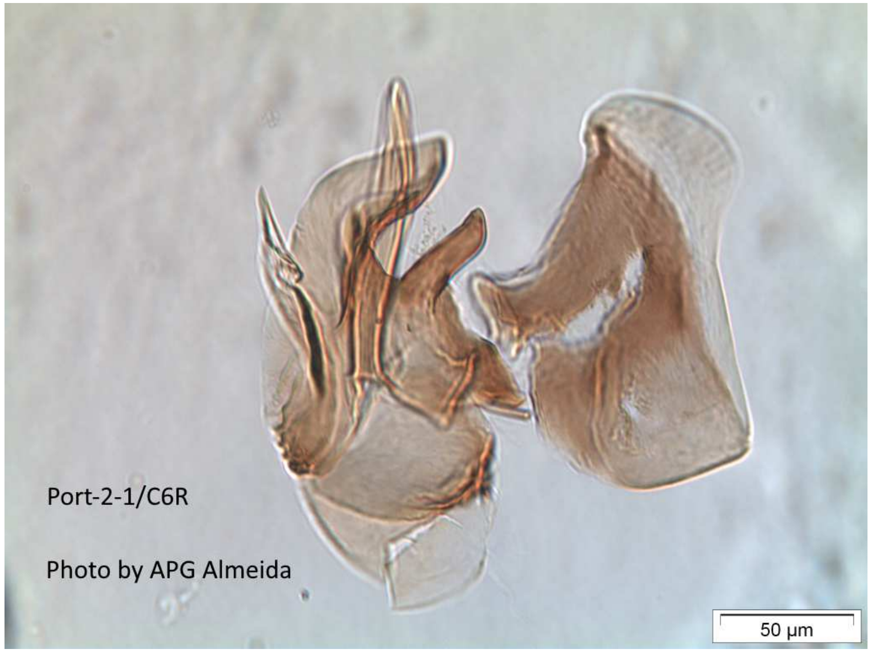

Port-2.2/C7; Gonocoxite, X100

- 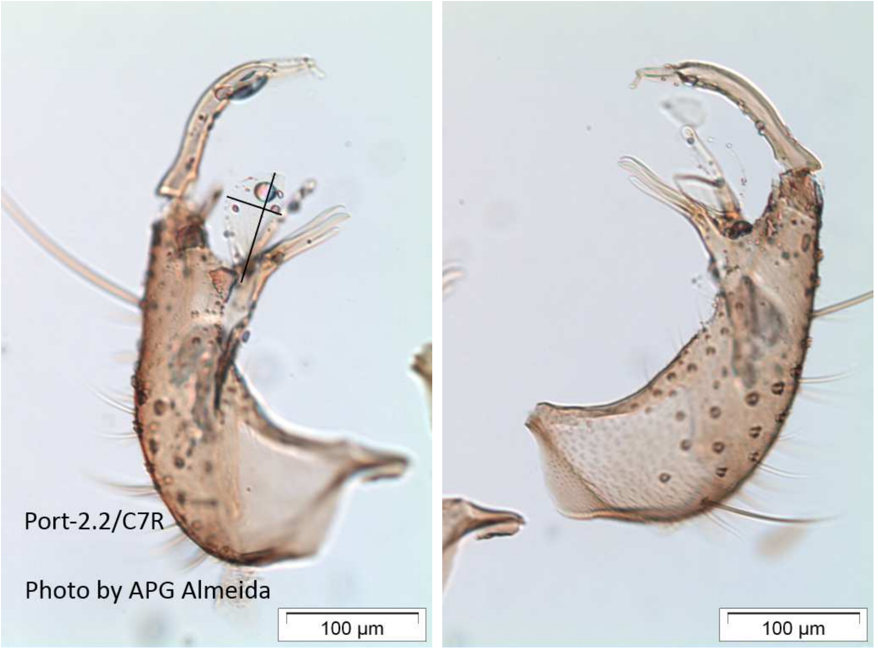

Port-2.2/C7; Phalosome, X200

•

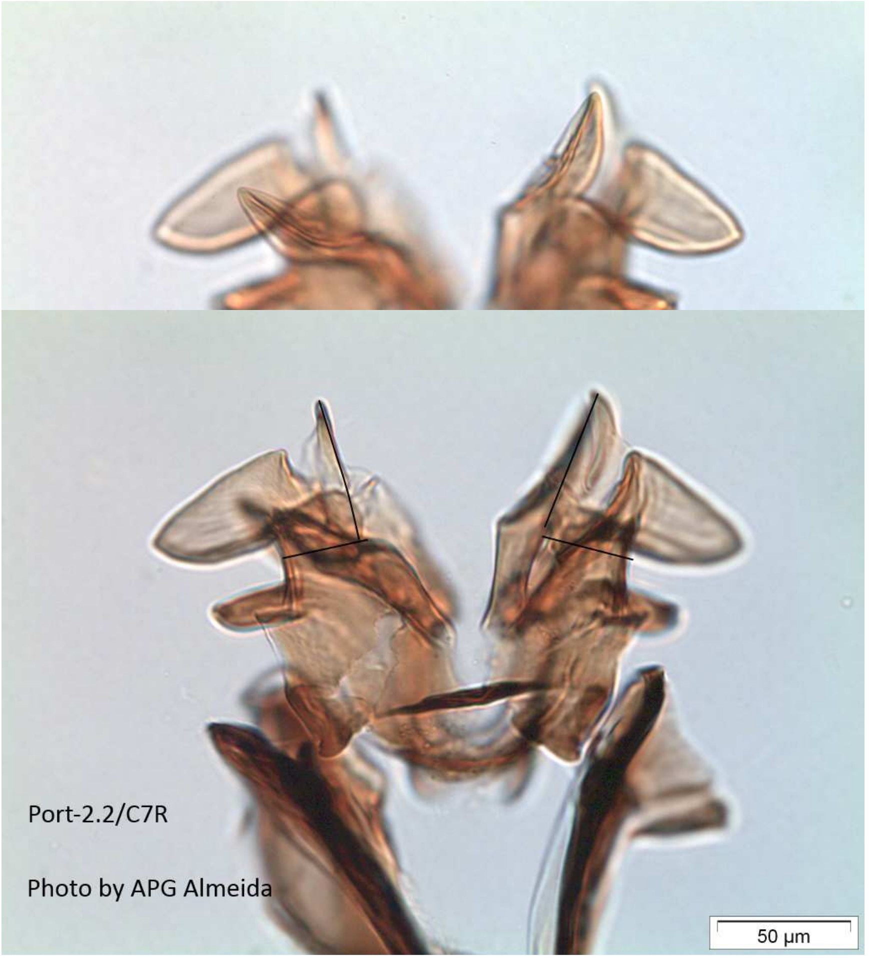

Port-2.2/C7; Phalosome, X200

- (remounted)

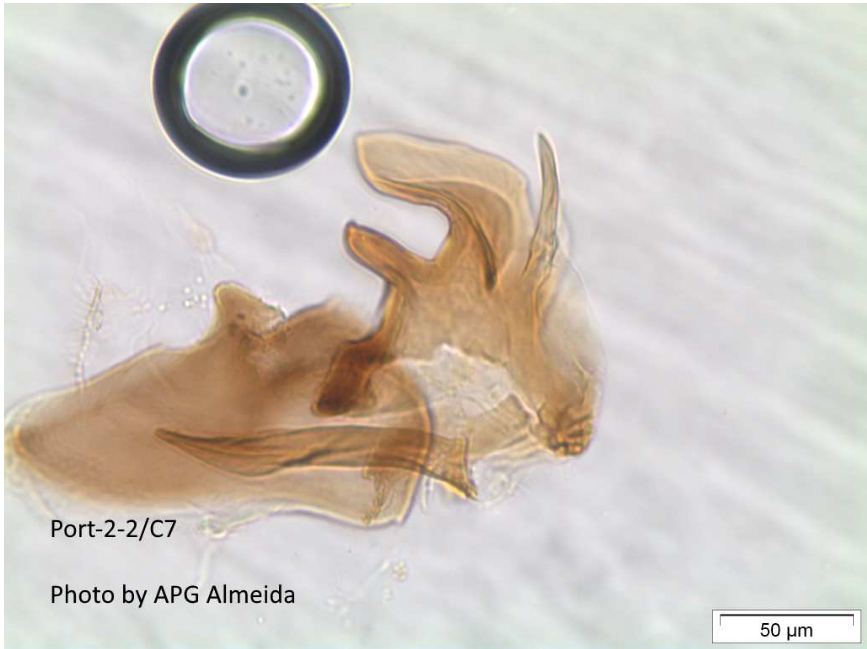

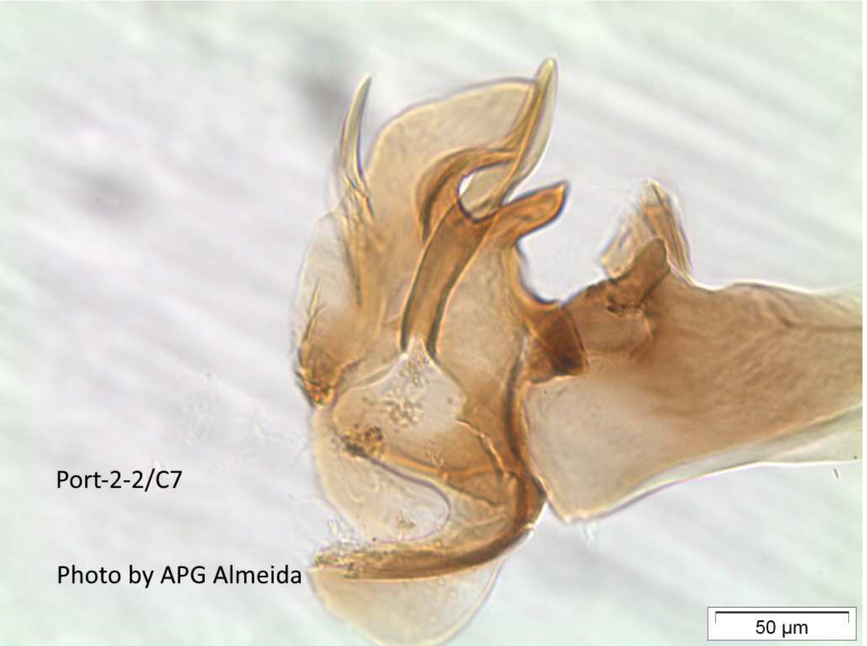

Port-2448.1/C1; Gonocoxite, X200

- 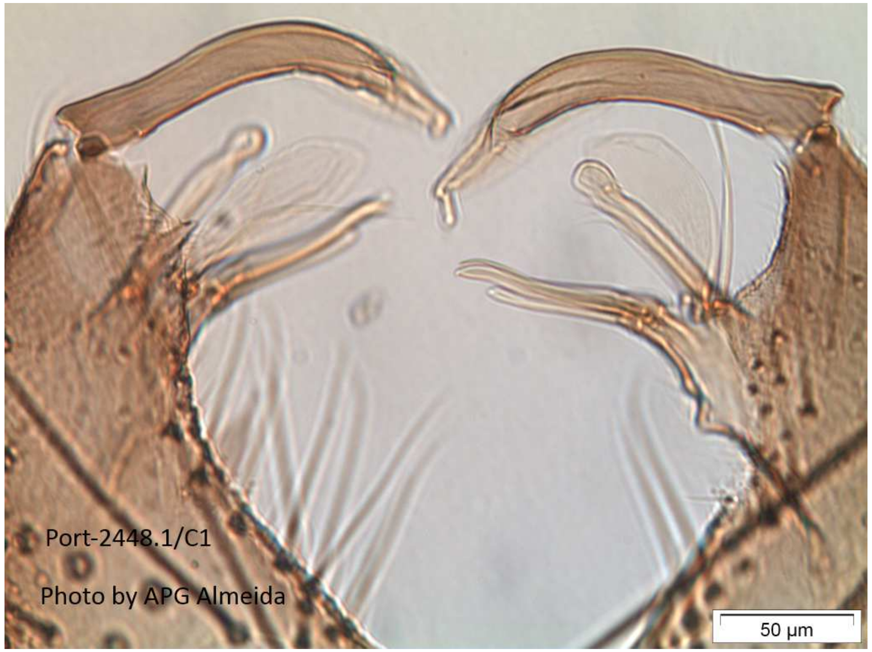

Port-2448.1/C1; Phalosome, X200

- 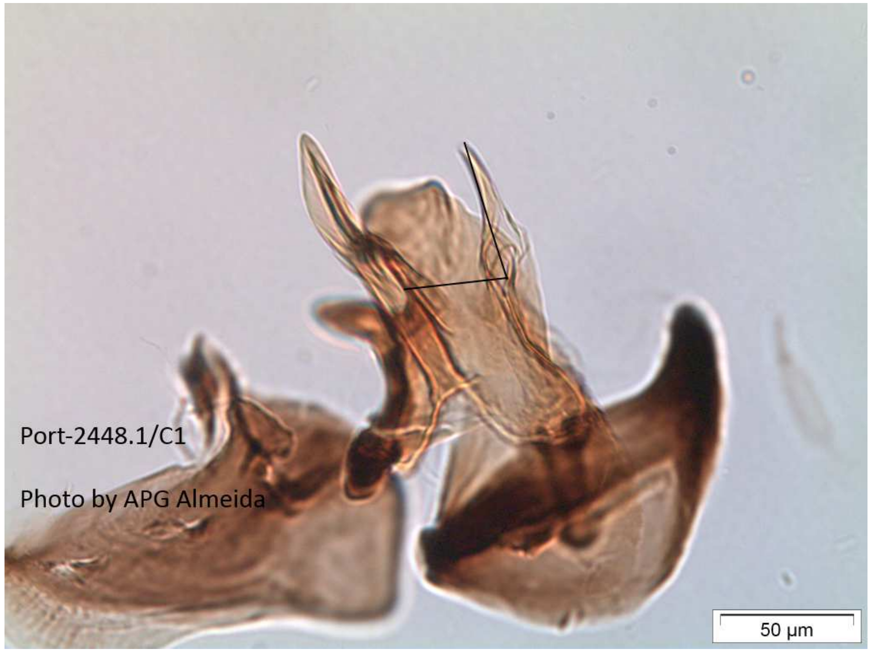

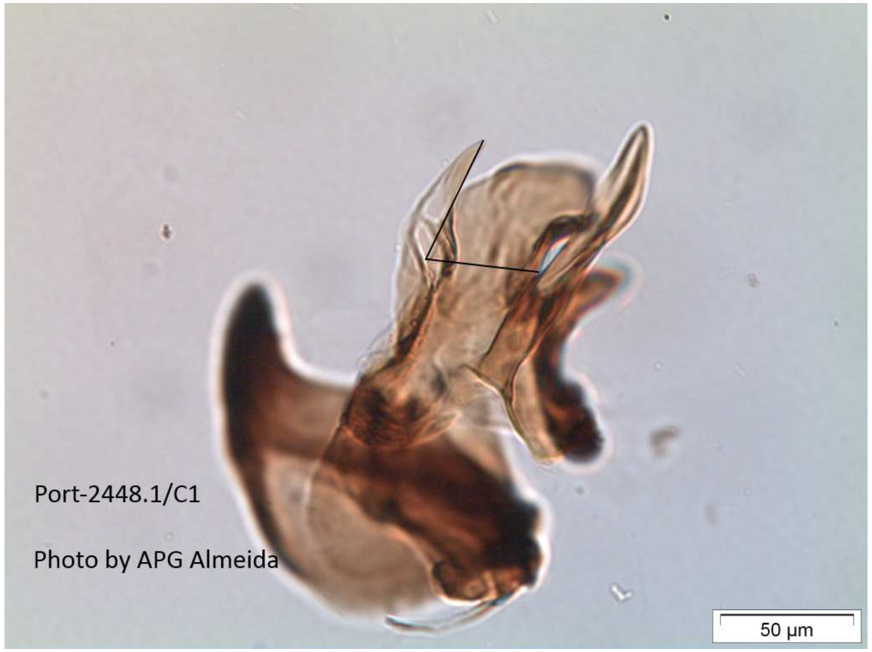

Port-2449.1; Gonocoxite, X100

- 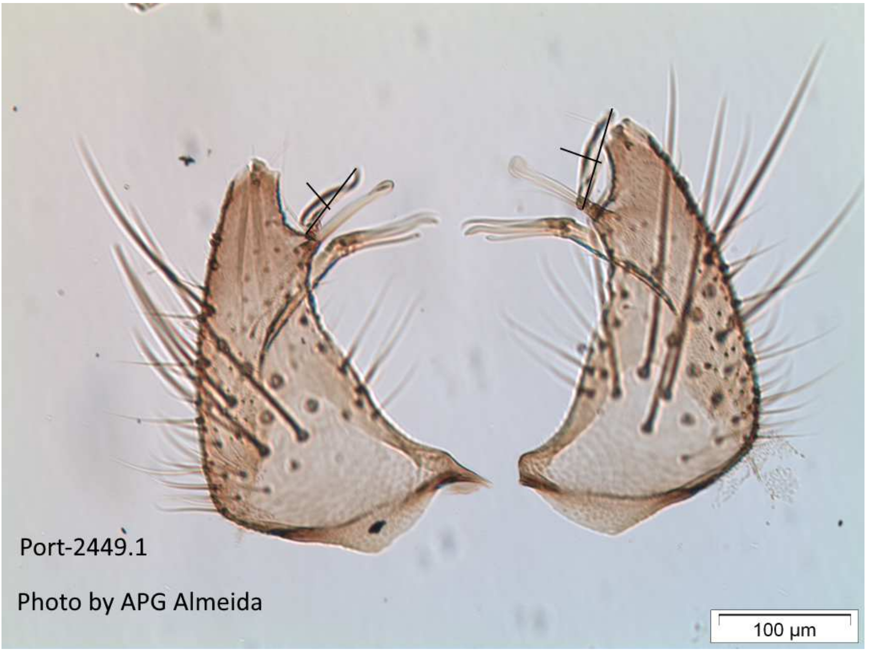

Port-2449.1; Phalosome, X200

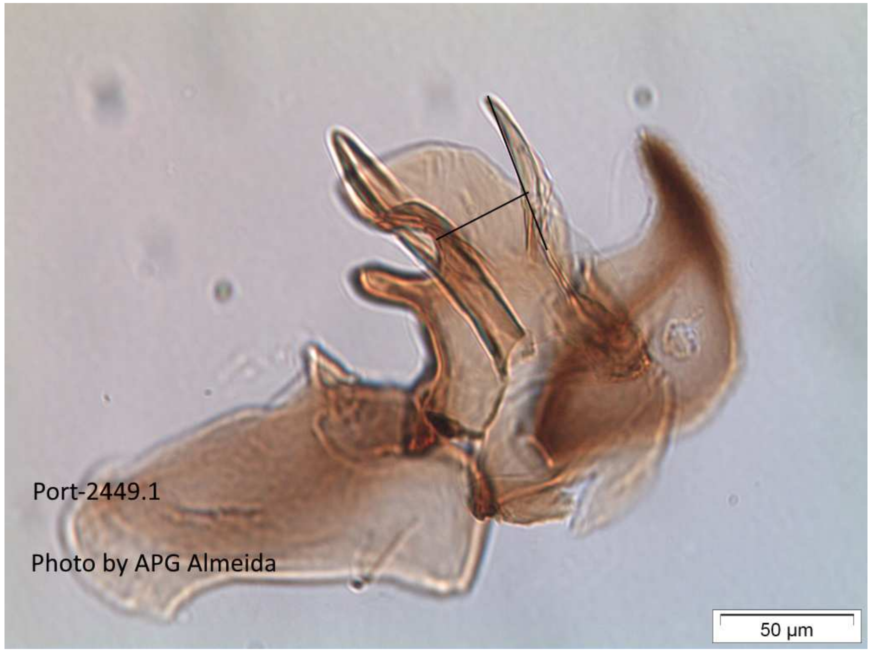

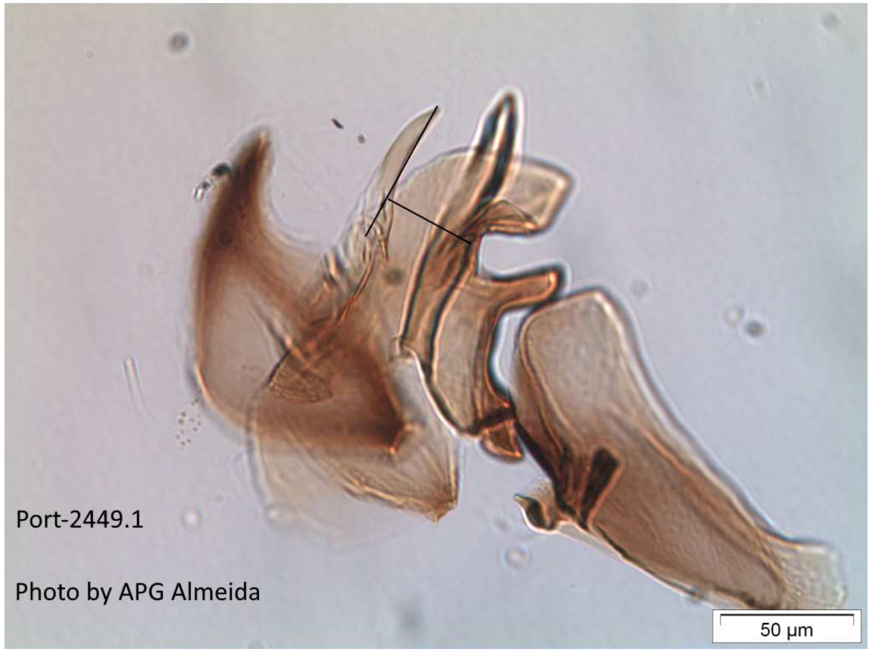

Port-2460.1; Gonocoxite, X100

- 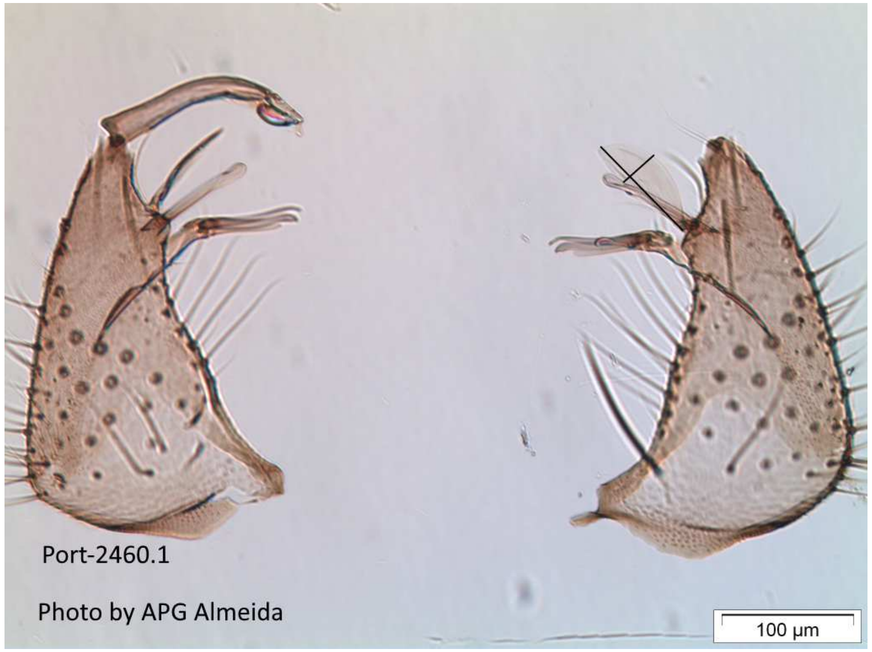

Port-2460.1; Phalosome, X200

•

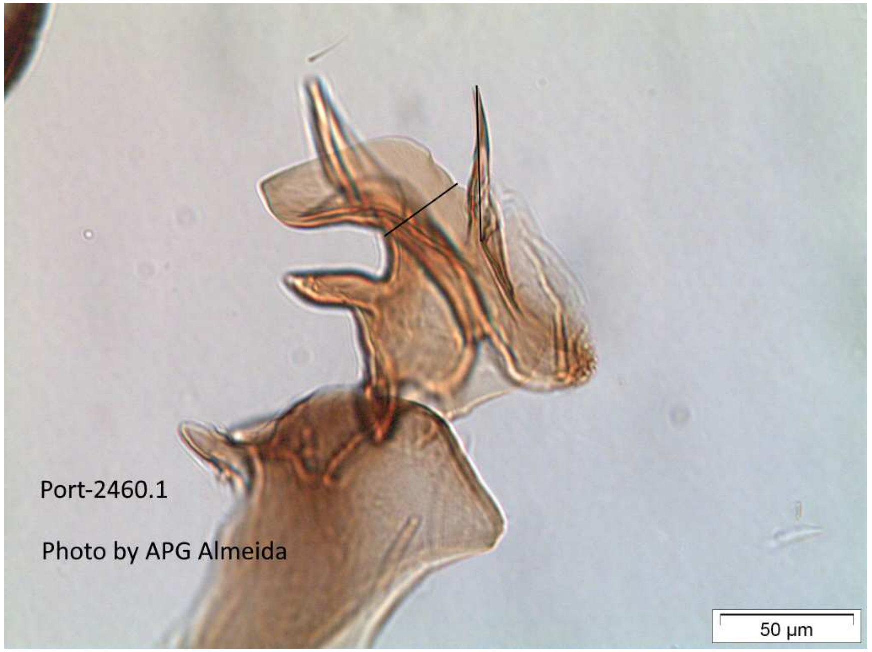

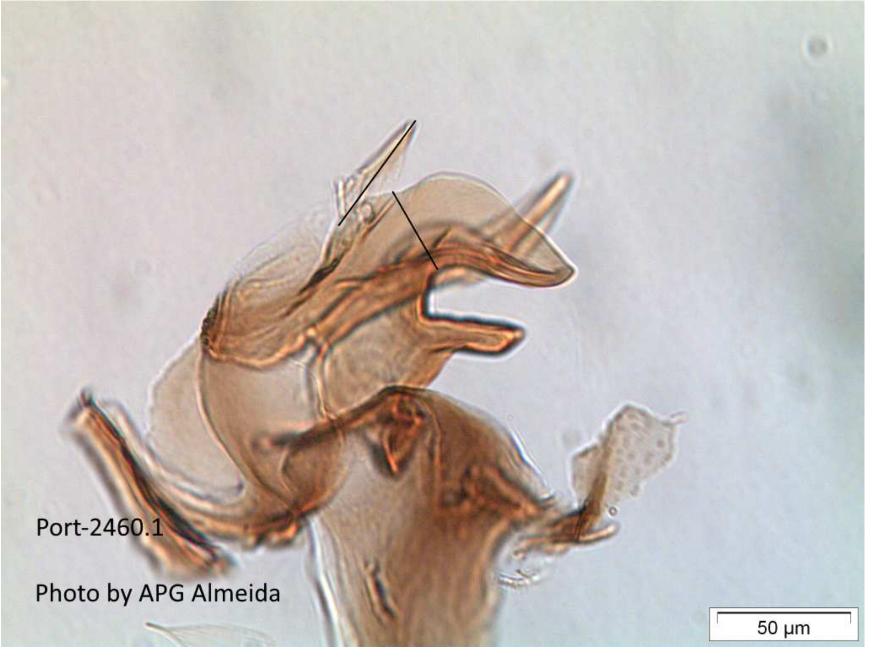

Port-2465.1/C4; Gonocoxite, X100

•

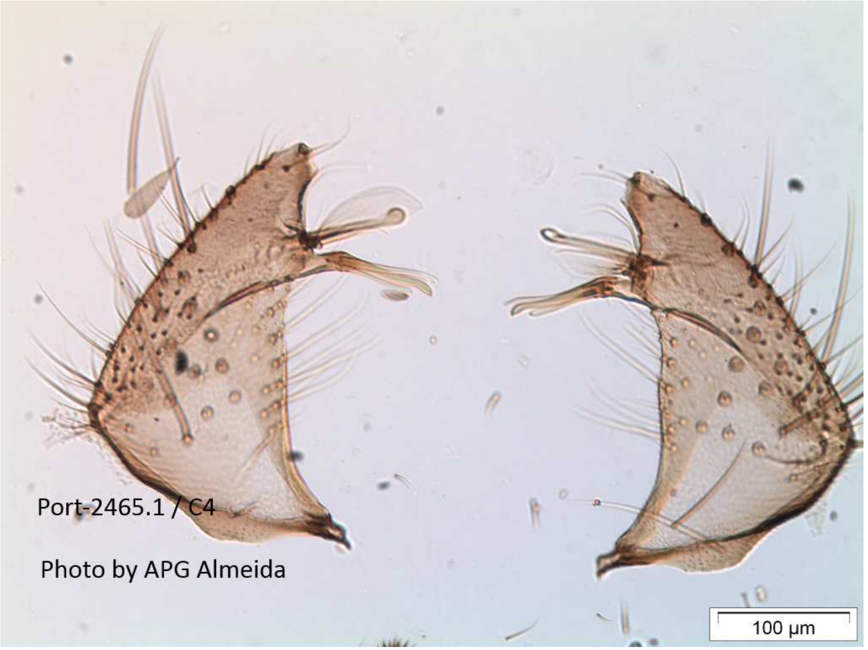

Port-2465.1/C4; Phalosome, X200

- 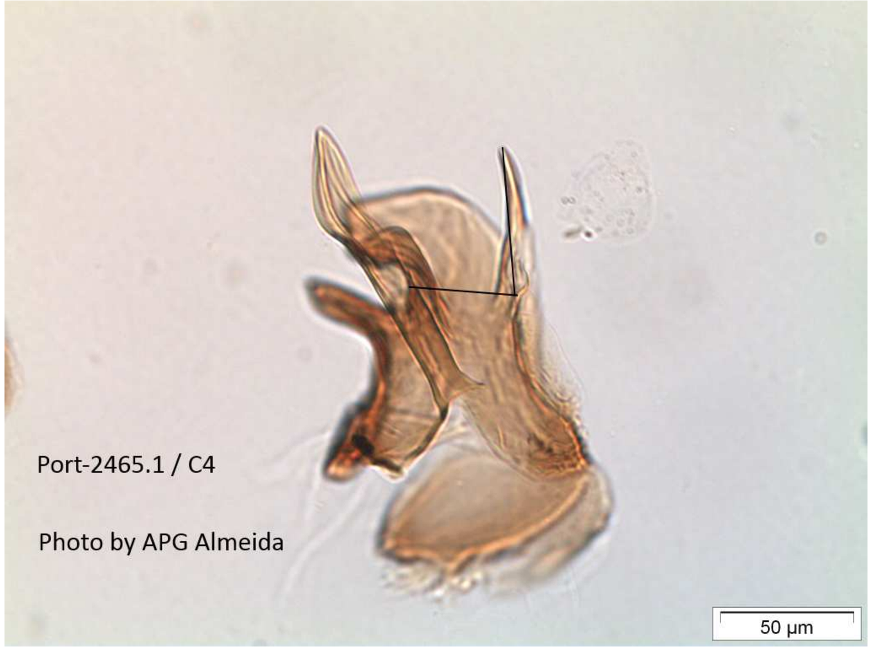

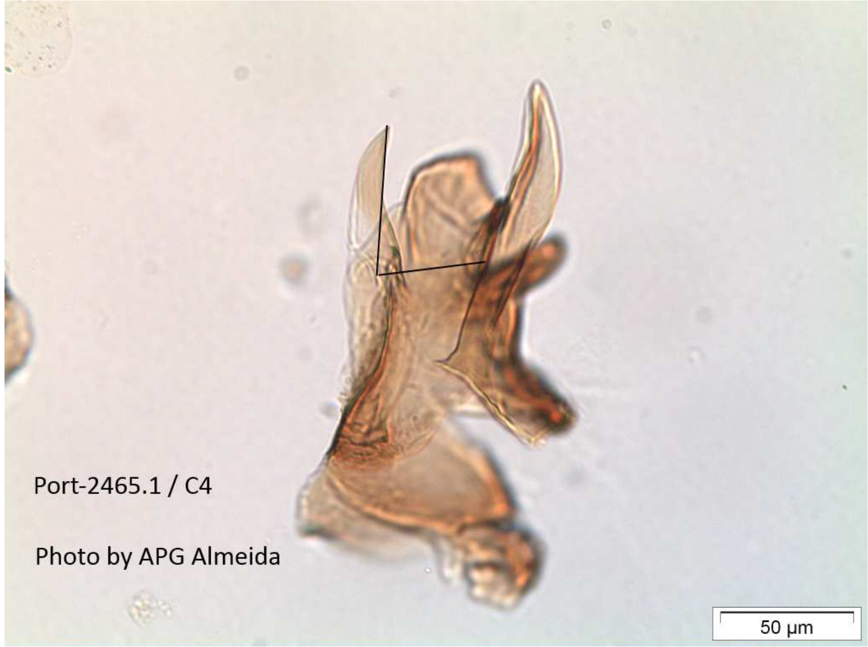

Port-2474.2; Gonocoxite, X100

- a marcação na foto está errada, é 2

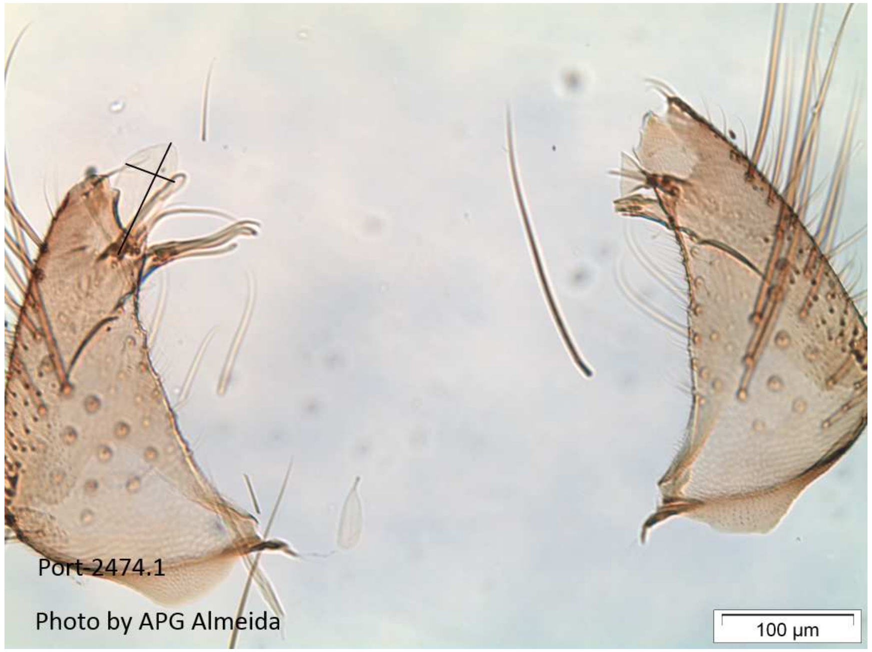

Port-2474.2; Phalosome, X200

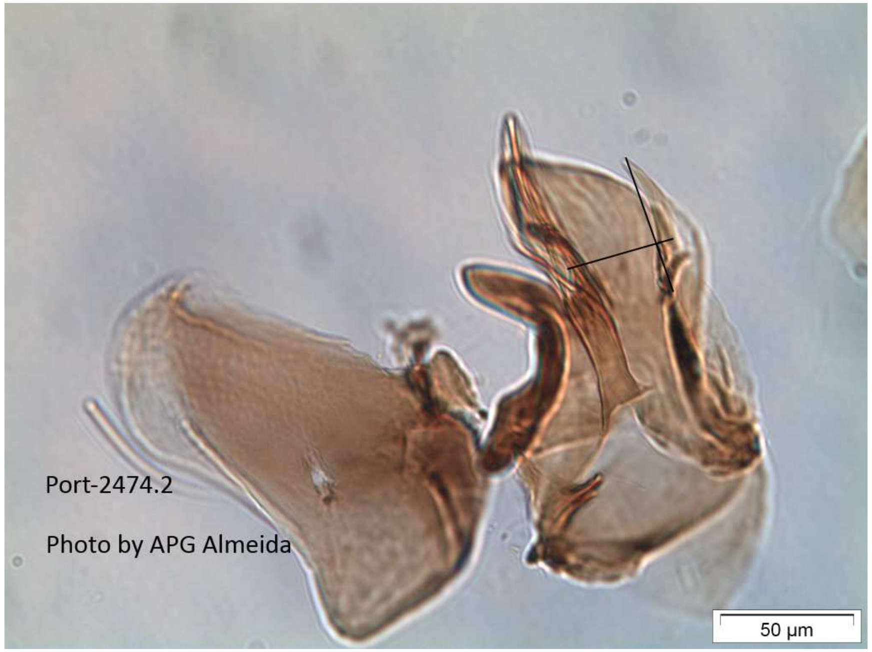

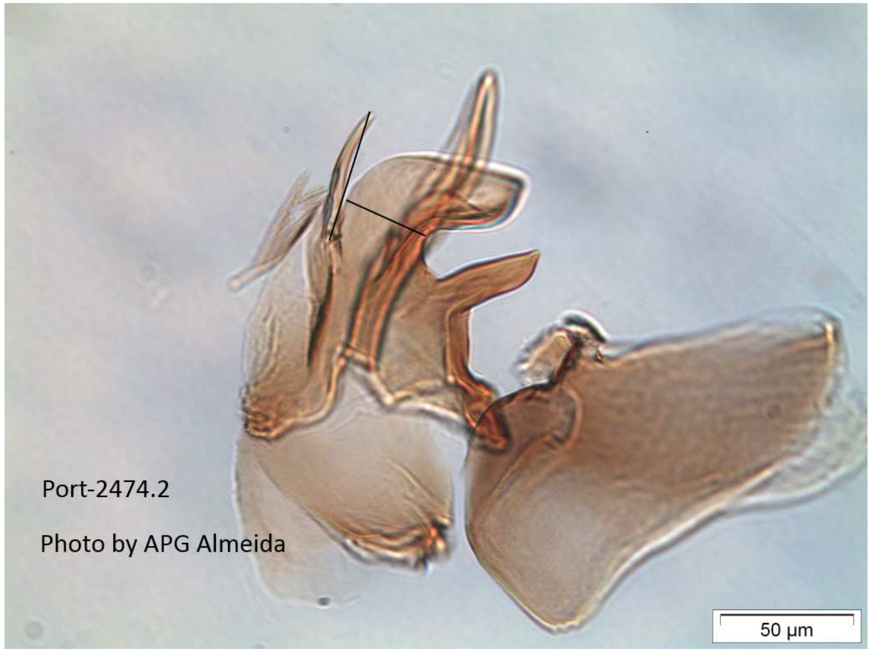

Port-2630.69/3435; Gonocoxite, X100

- (first and second mounting)

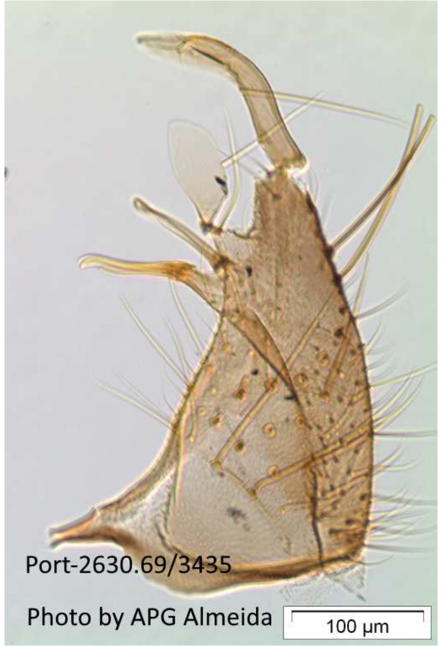

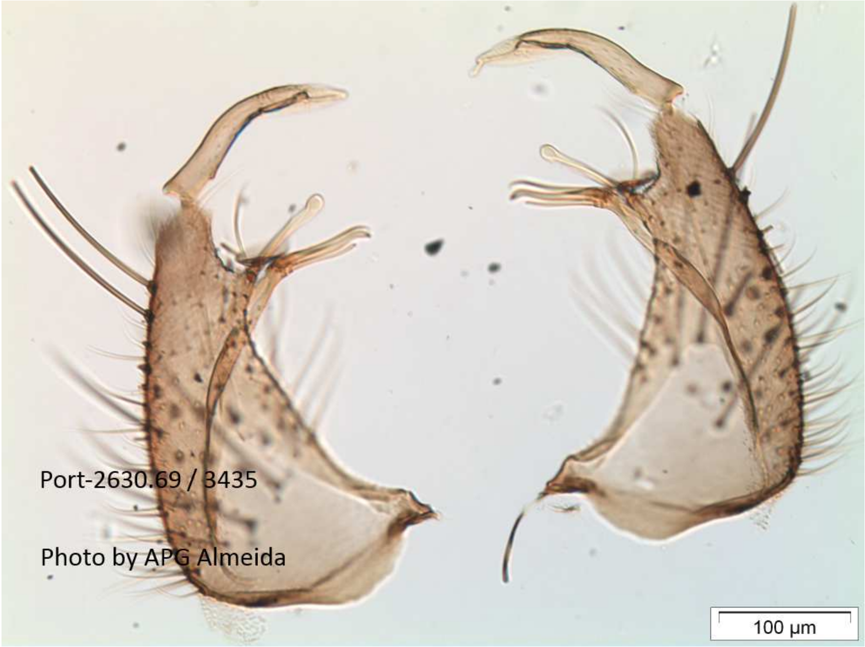

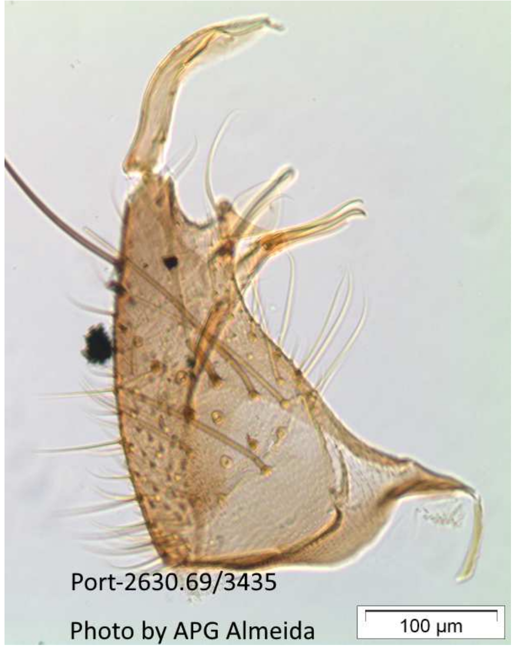

Port-2630.69/3435; Phalosome, X200

•

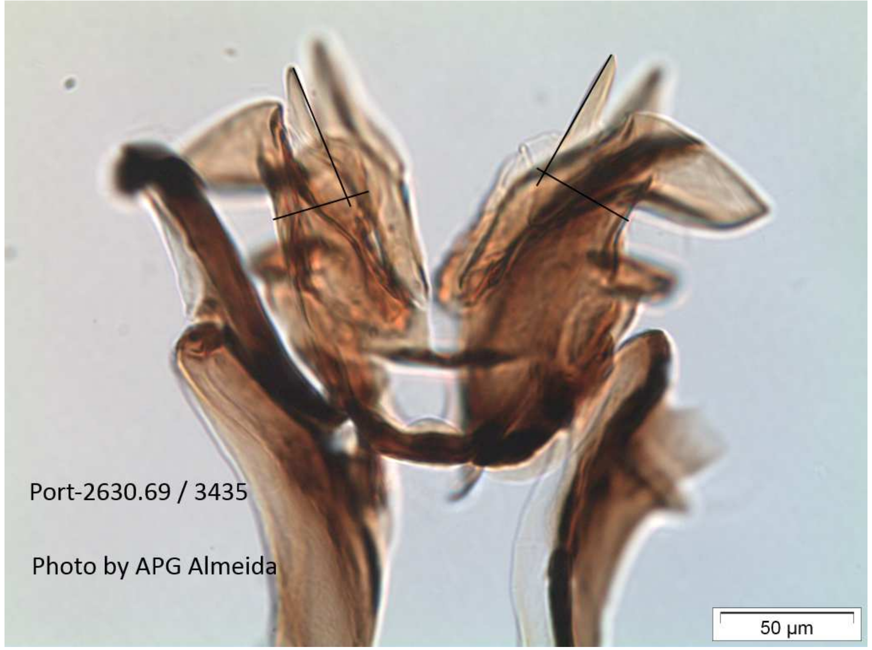

Port-2630.69/3435; Phalosome, X200

- (remounted)

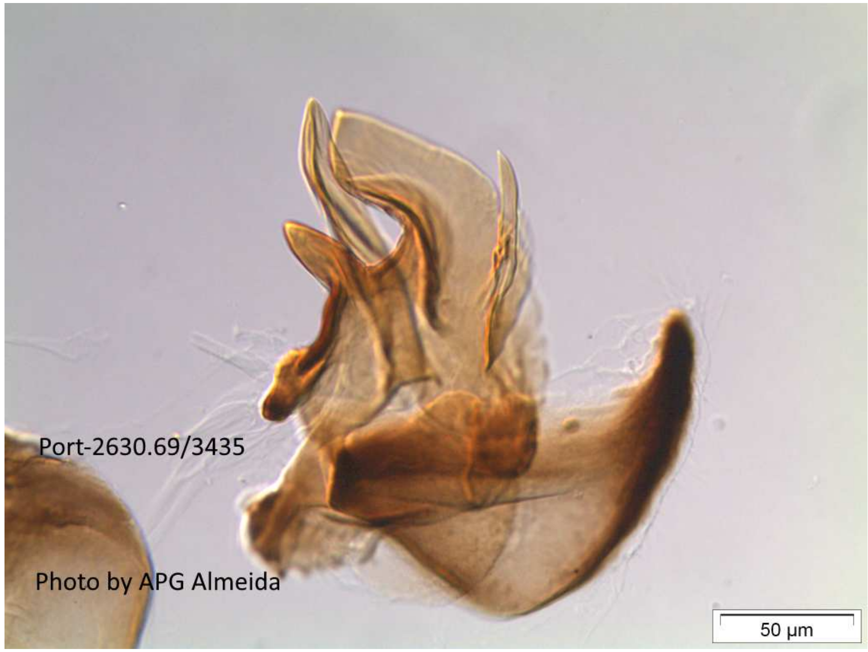

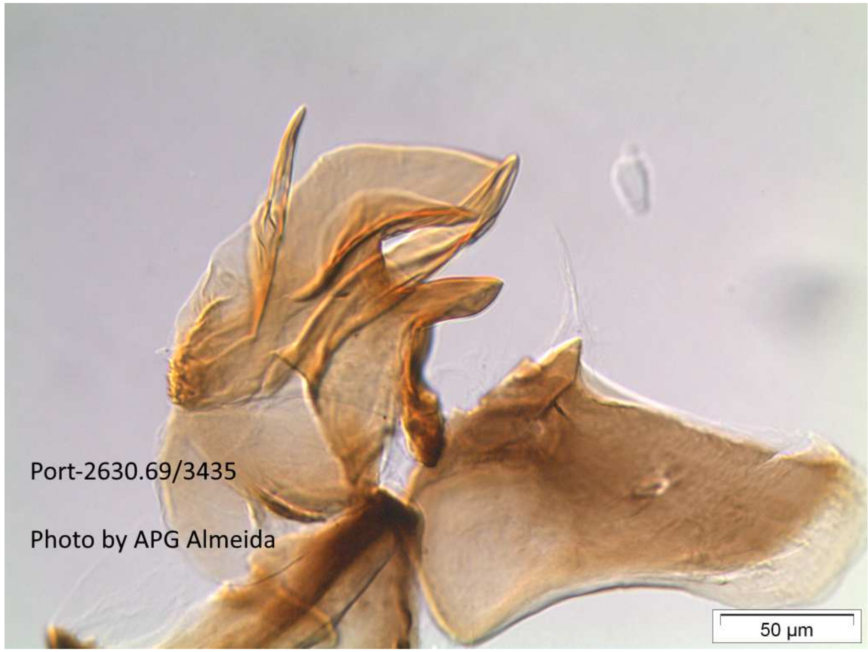

Port-3437; Gonocoxite, X200

- 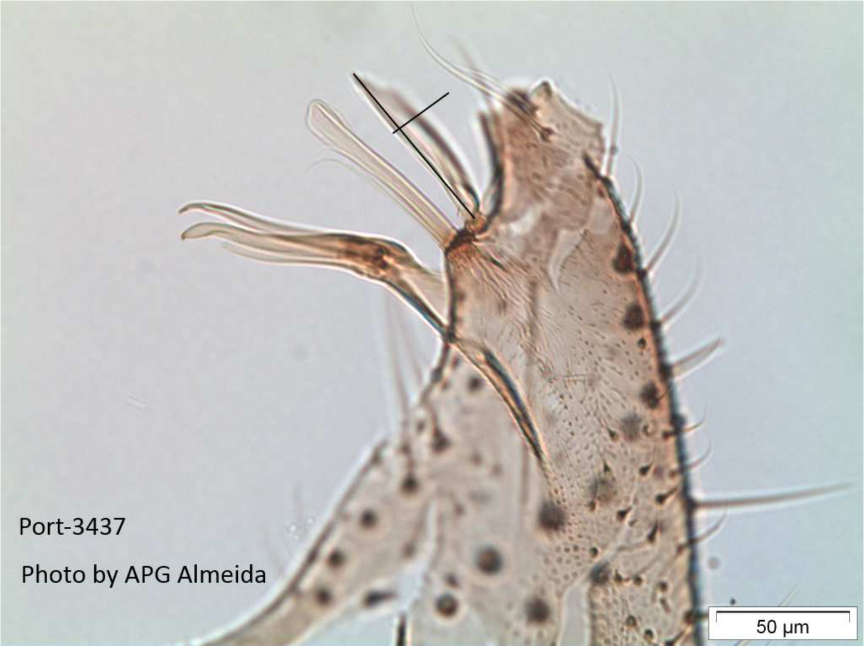

Port-3437; Phalosome, X200

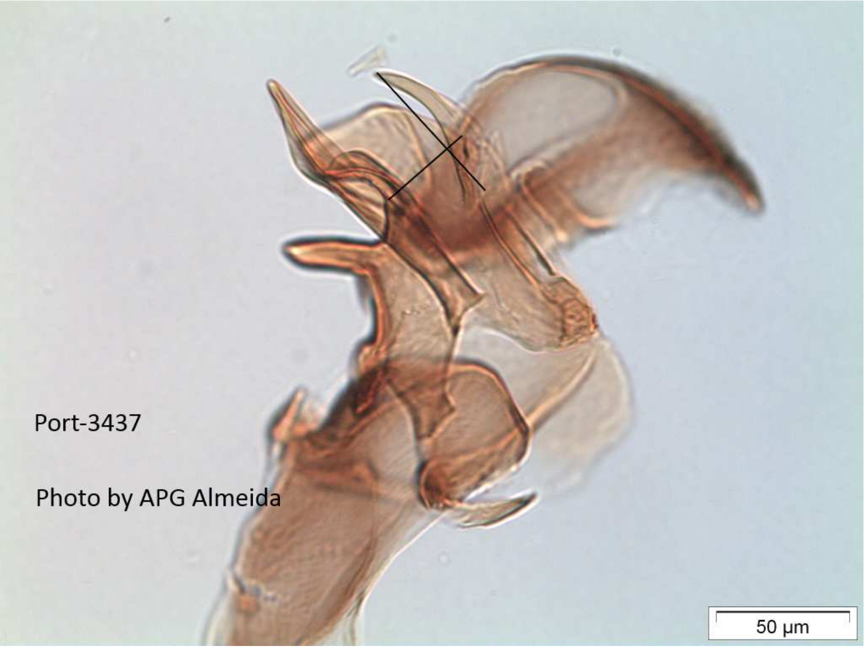

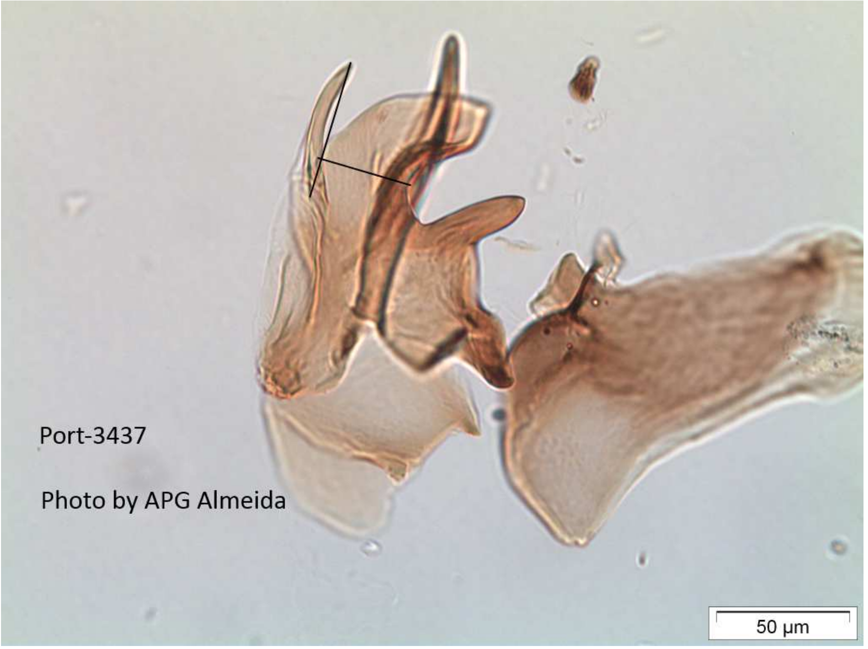

Port-3438; Gonocoxite, X100

•

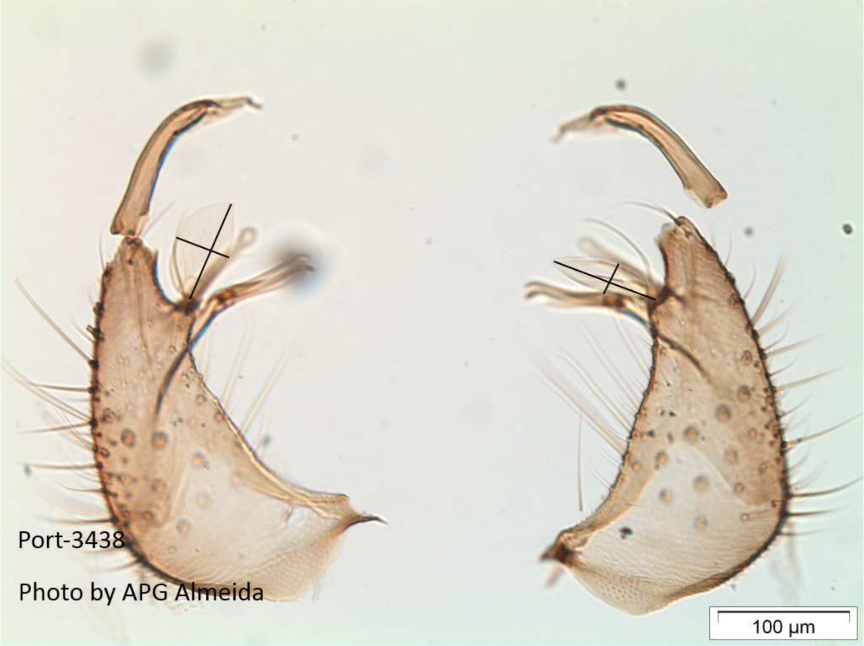

Port-3438; Phalosome, X200

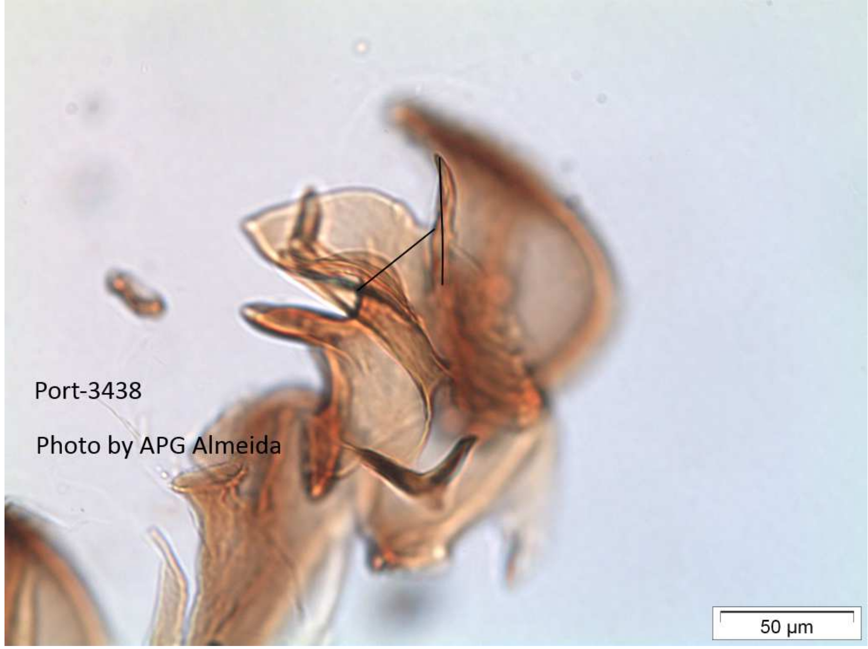

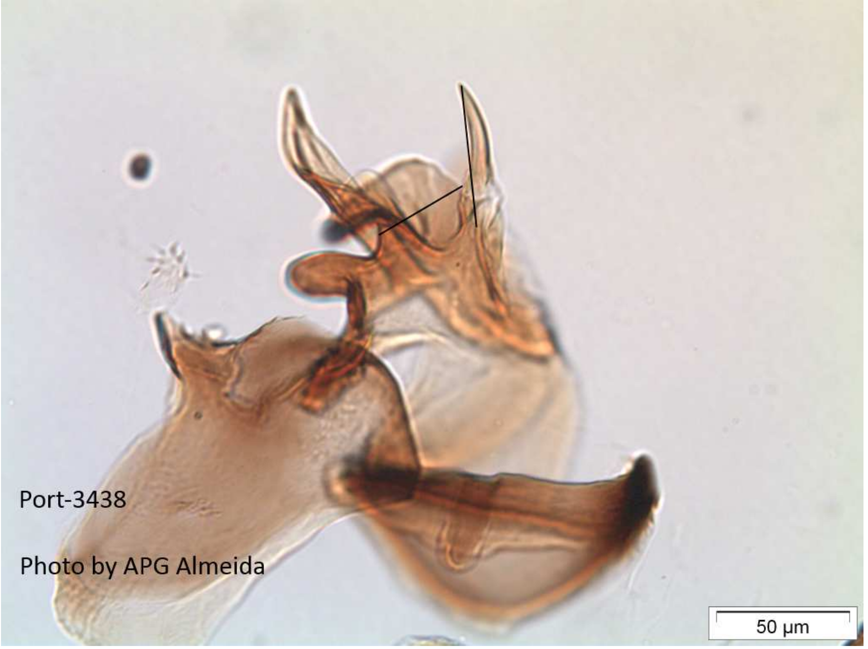

Port-3439; Gonocoxite, X100

•

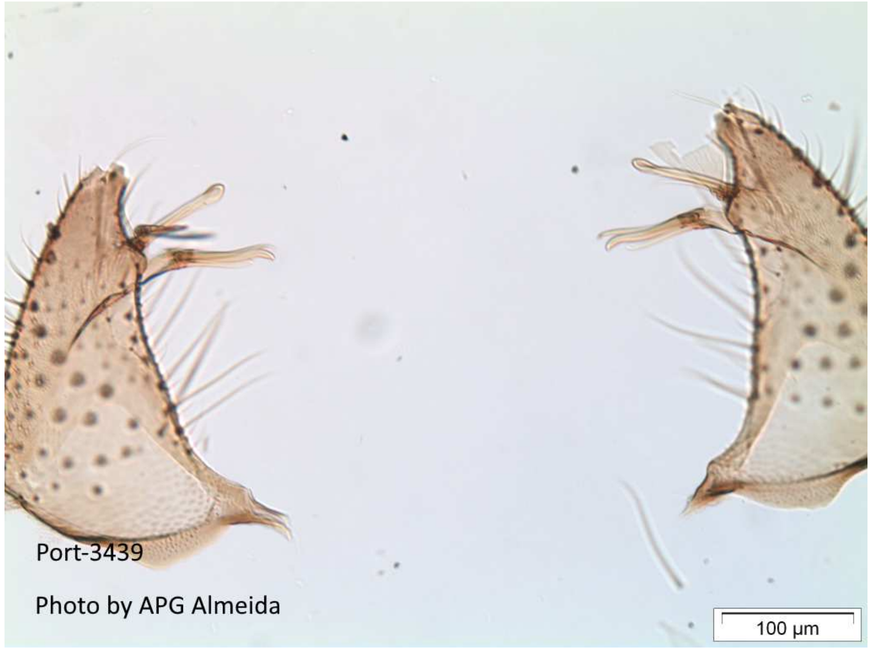

Port-3439; Phalosome, X200

- 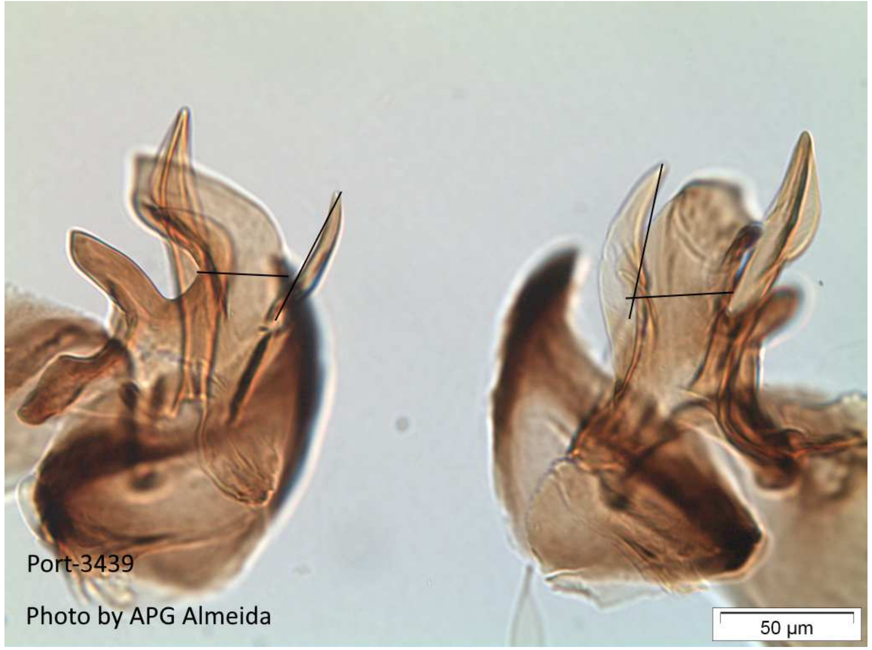

SAfr-GAU86E1/GAU3; Gonocoxite, X100

- 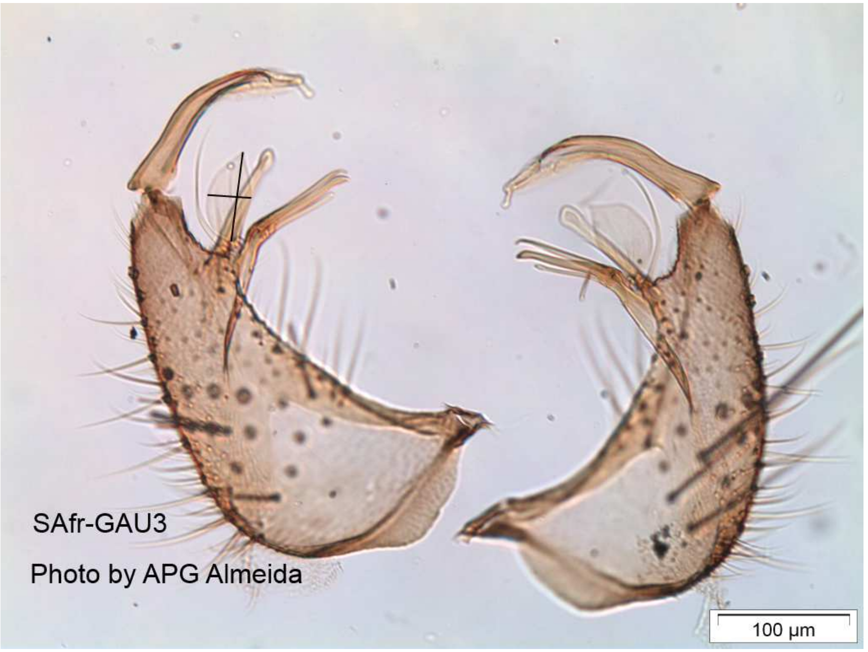

SAfr-GAU86E1/GAU3; Phalosome, X200

- 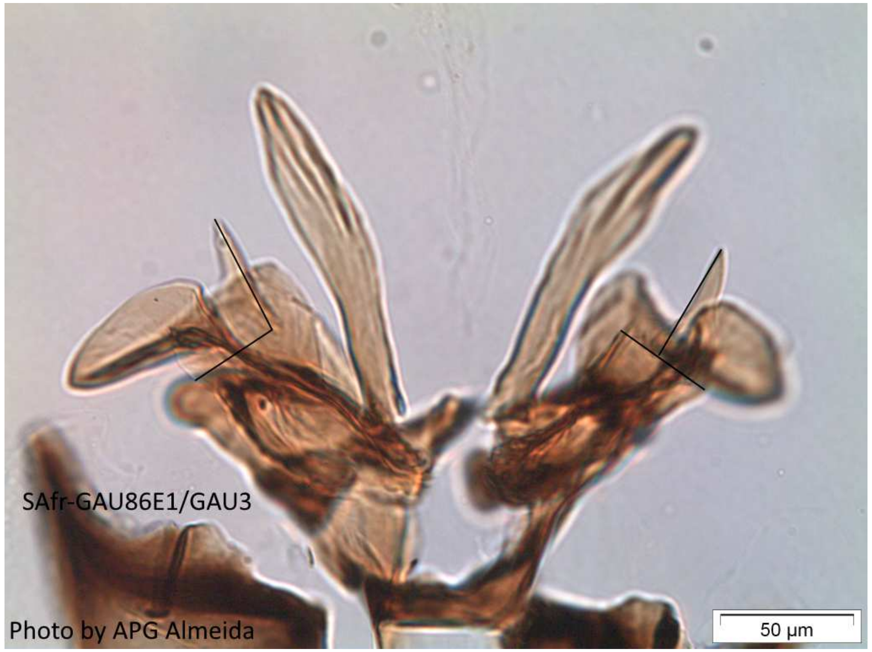

SAfr-GAU86E1/GAU3; Phalosome, X200

- (remounted)

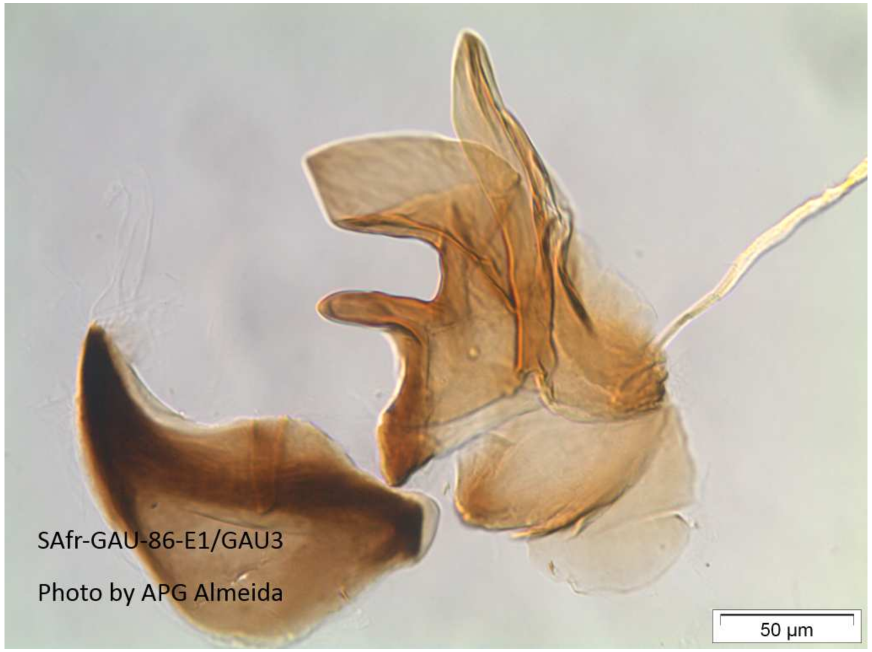

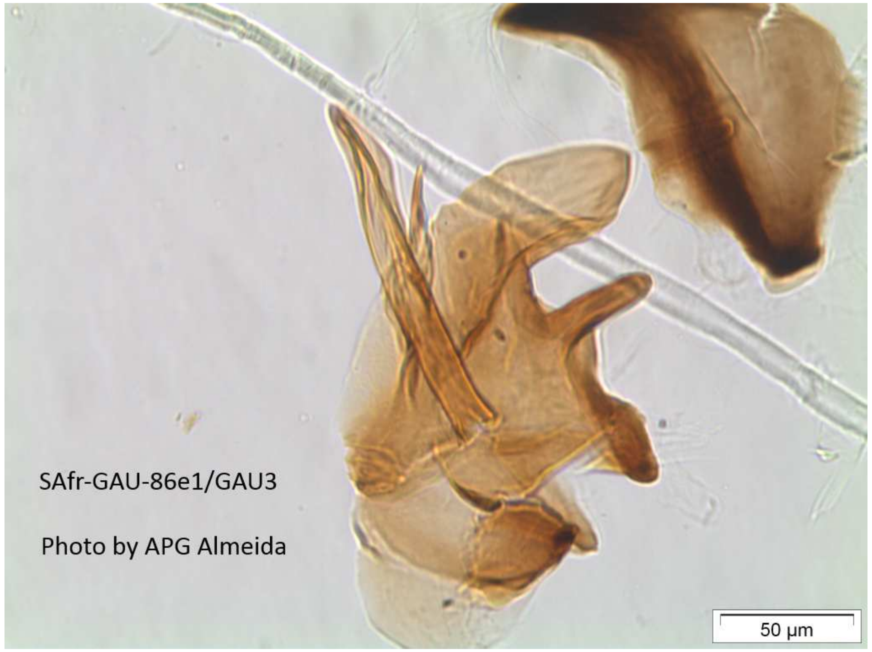

SAfr-GAU86E2/GAU4; Gonocoxite, X100

- 1st mounting

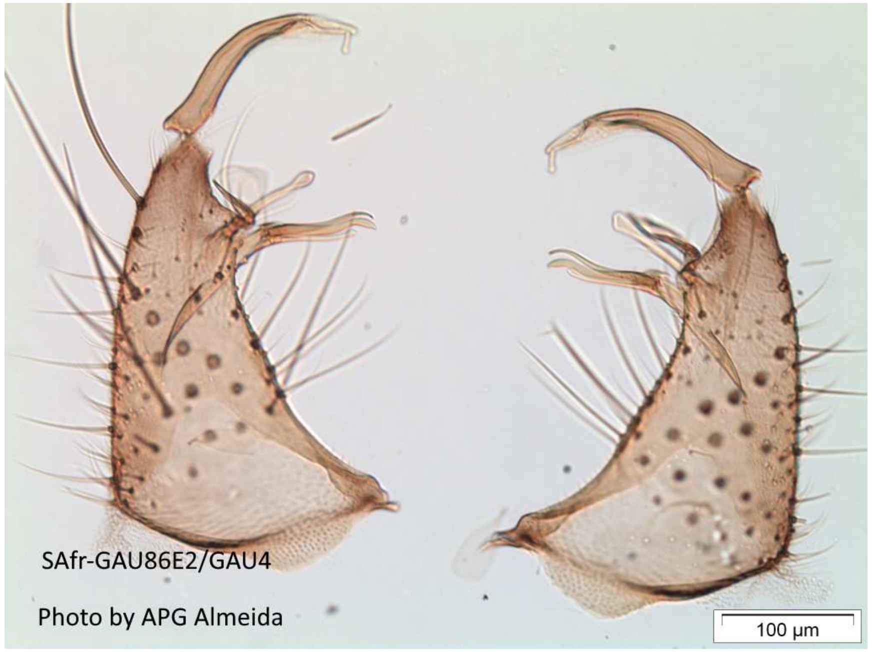

2nd mounting

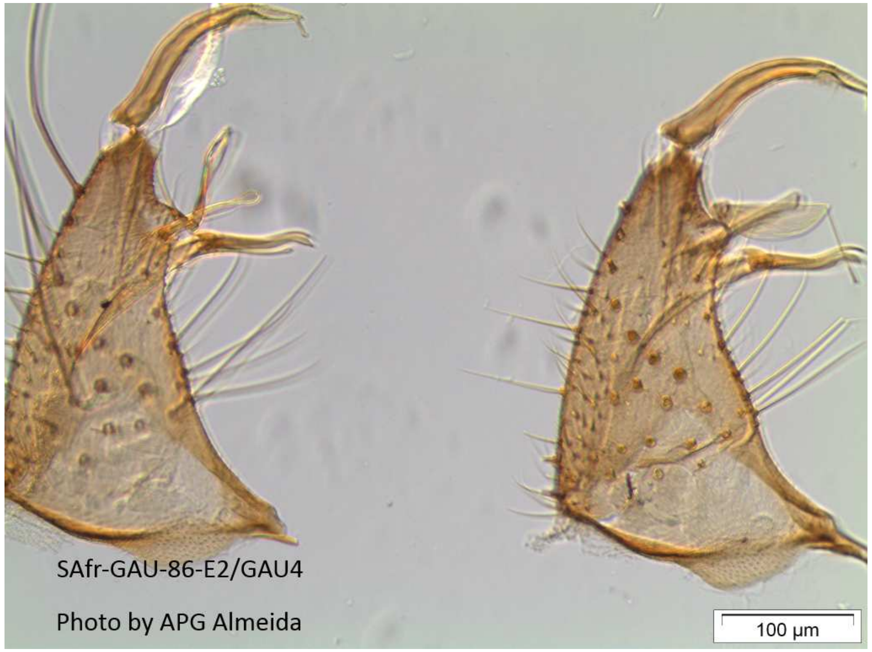

SAfr-GAU86E2/GAU4; Phalosome, X200

-

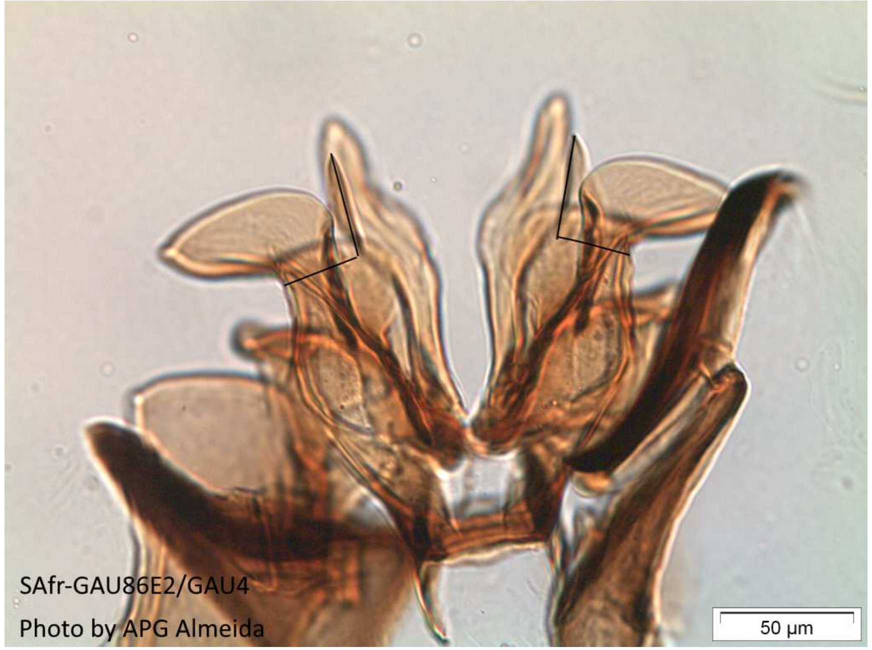

SAfr-GAU86E2/GAU4; Phalosome, X200

- (remounted)

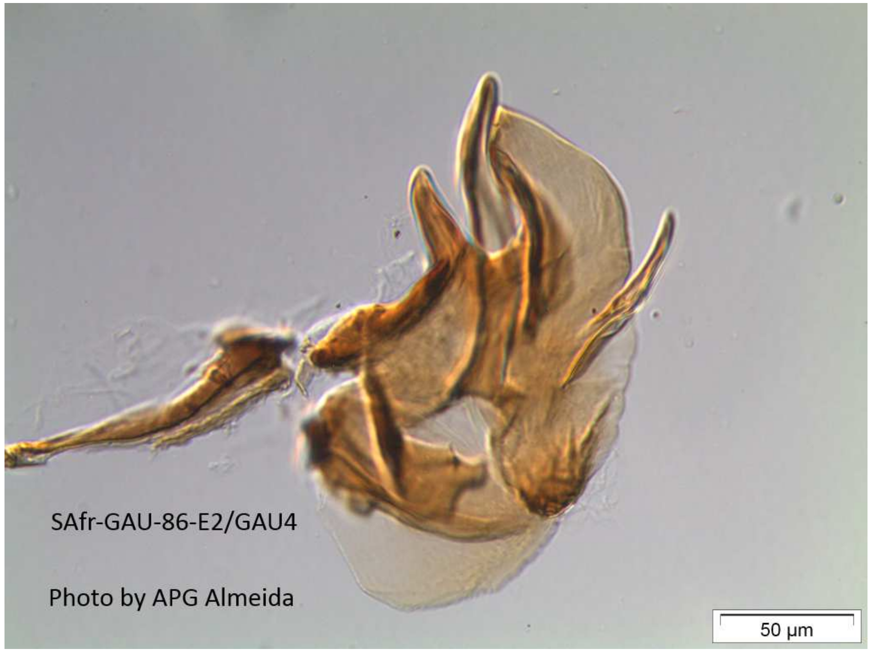

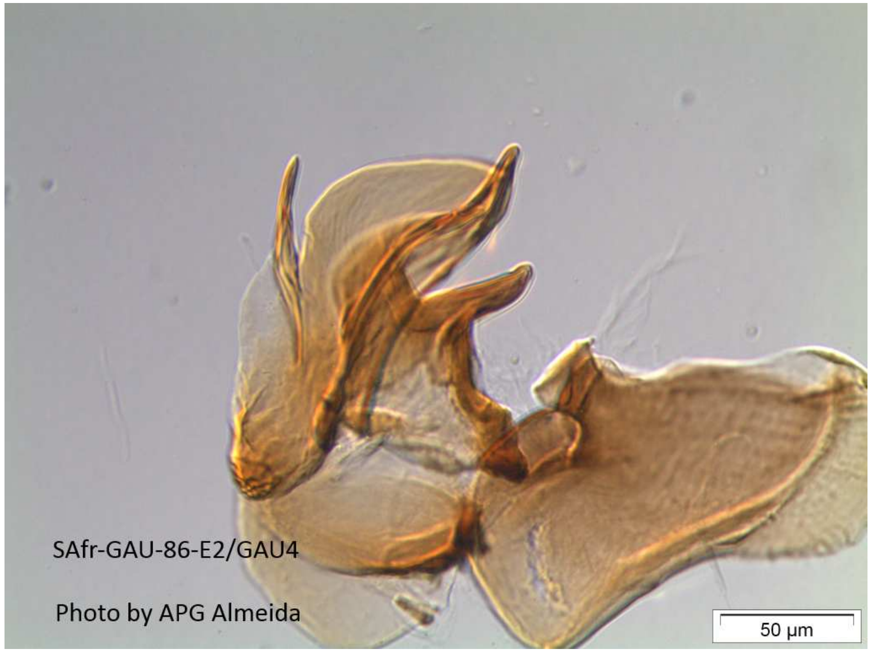

SAfr-GAU109K/GAU9; Phalosome, X200

- (Gonocoxite has been lost)

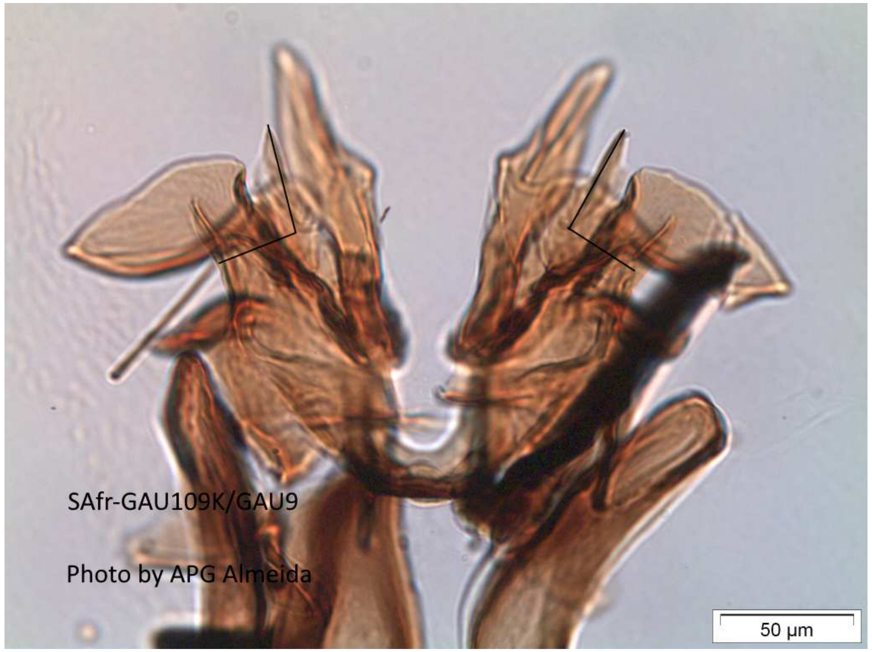

SAfr-GAU109K/GAU9; Phalosome, X200

- (remounted)

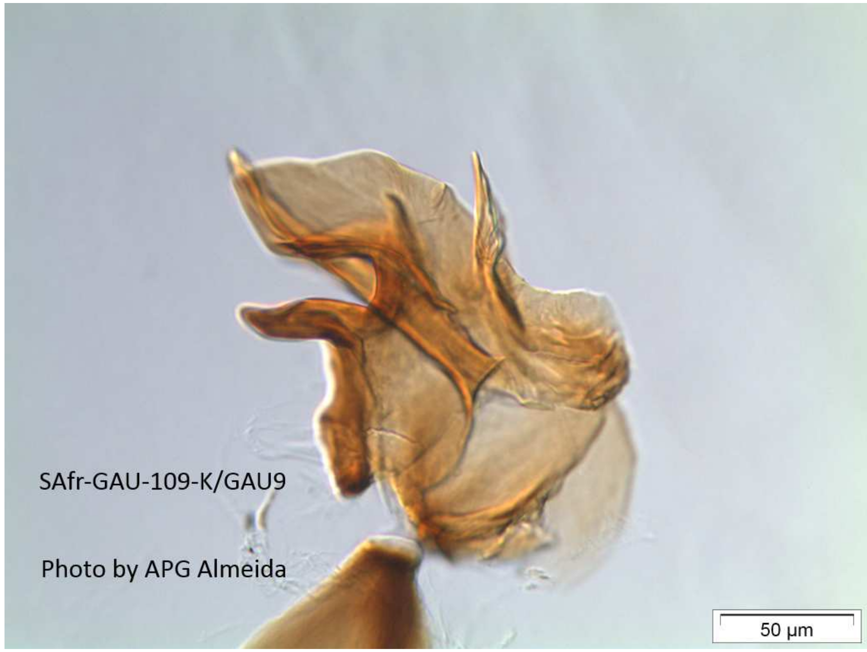

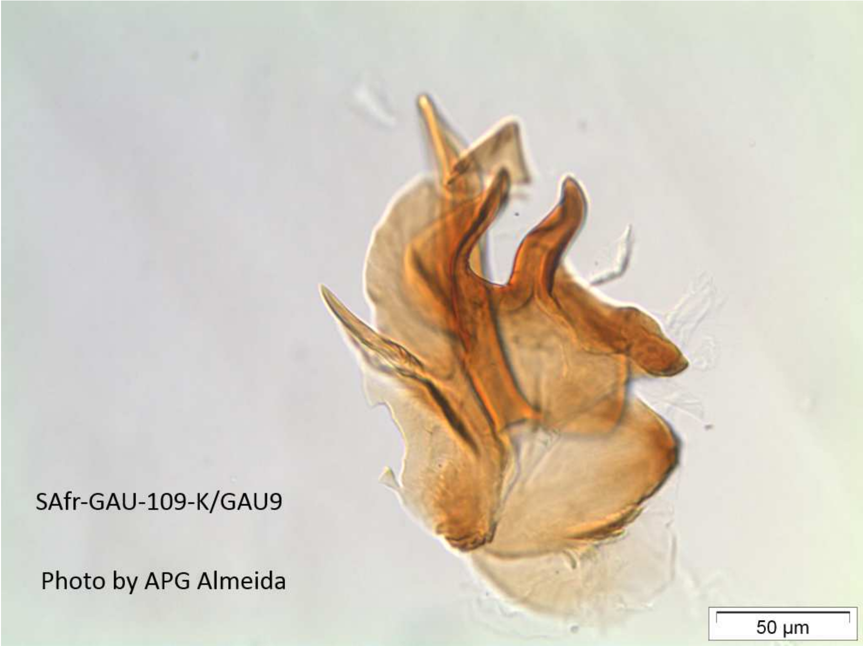

SAfr-GAU113H/GAU10; Gonocoxite, X100

•

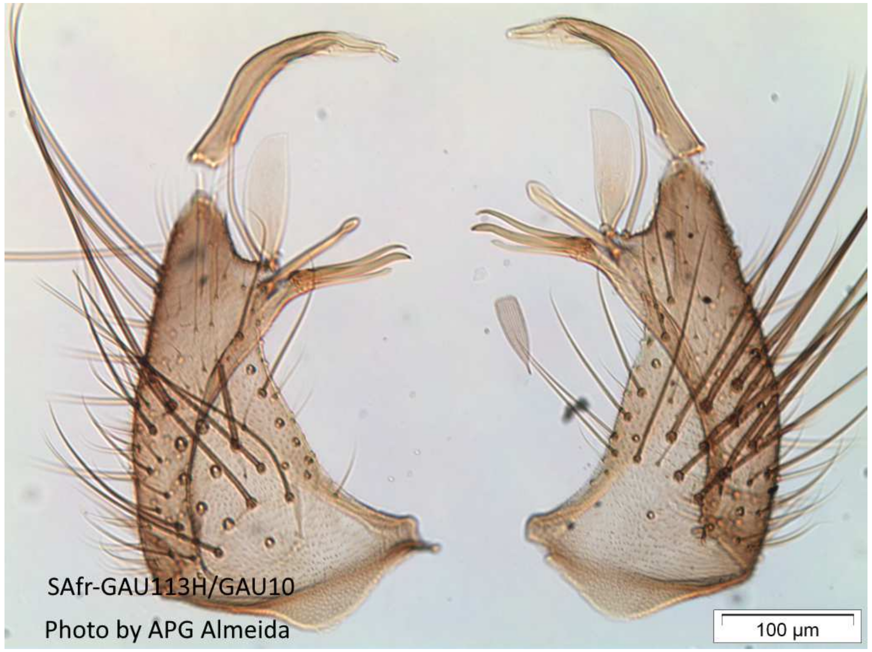

SAfr-GAU113H/GAU10; Phalosome, X200

•

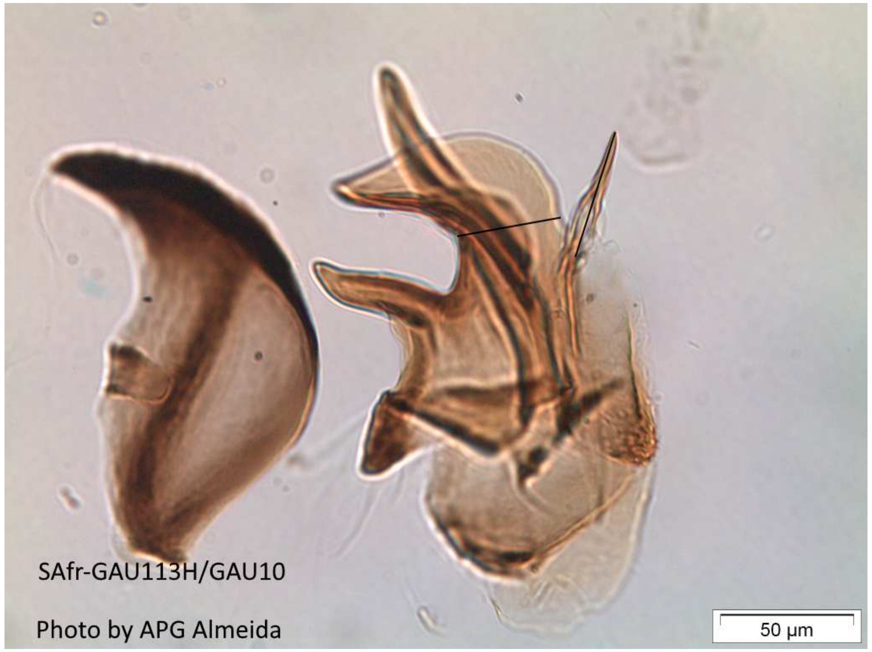

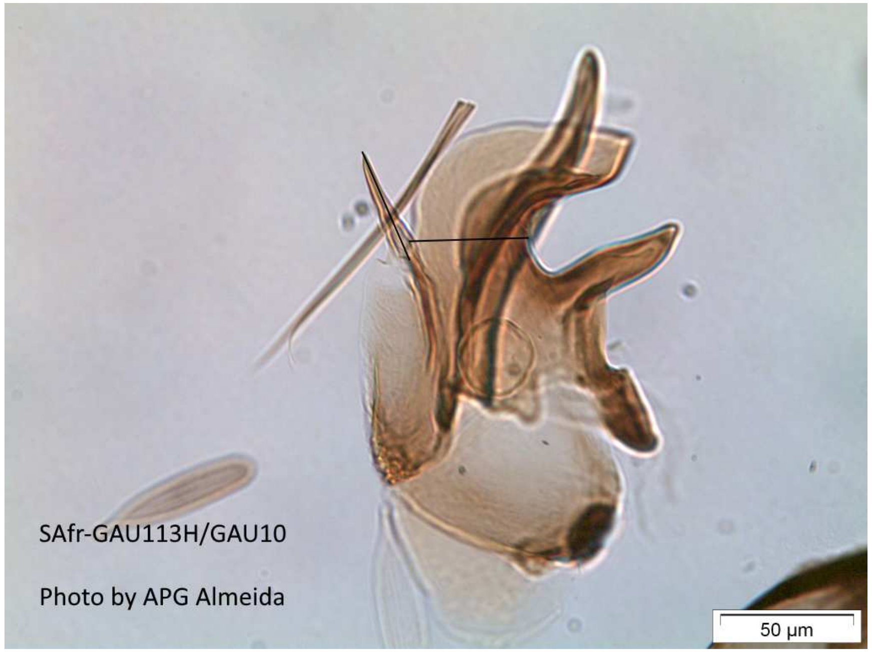

SAfr-GAU118M/GAU12; Gonocoxite, X100

-

(X200)

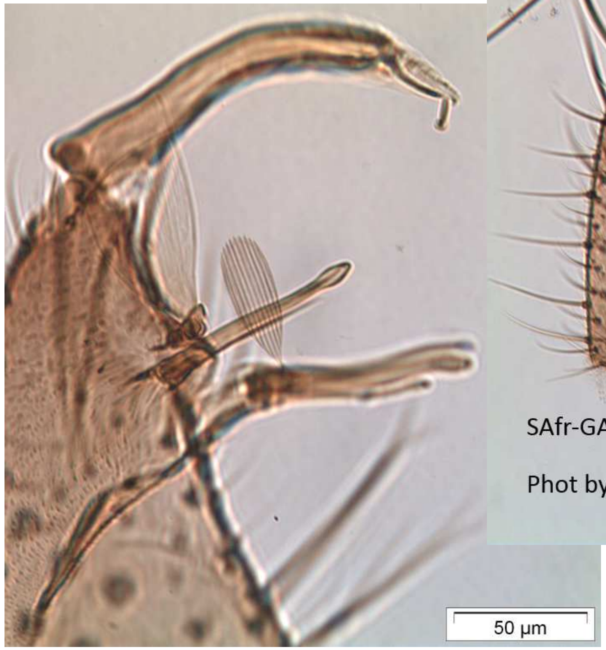

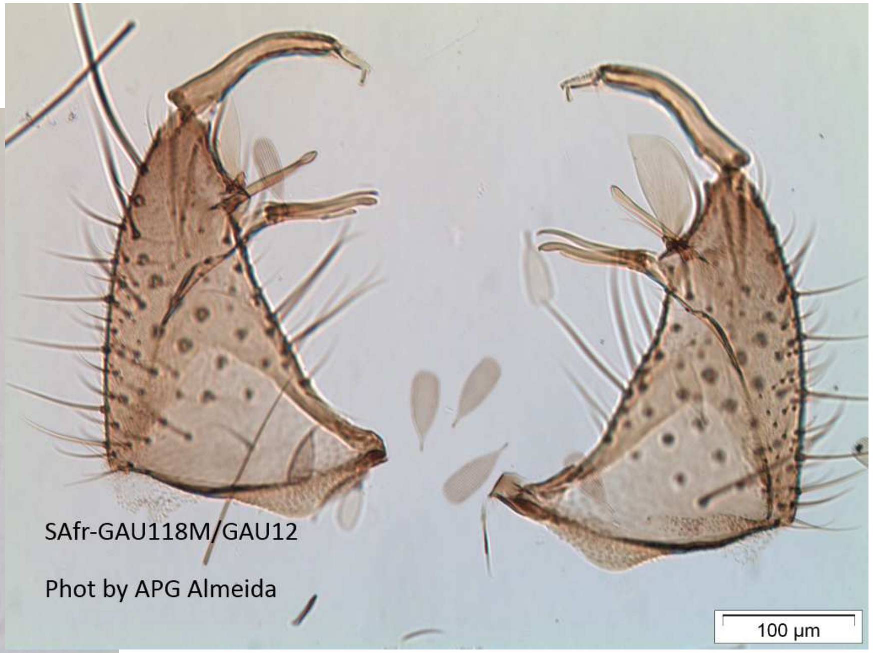

SAfr-GAU118M/GAU12; Phalosome, X200

•

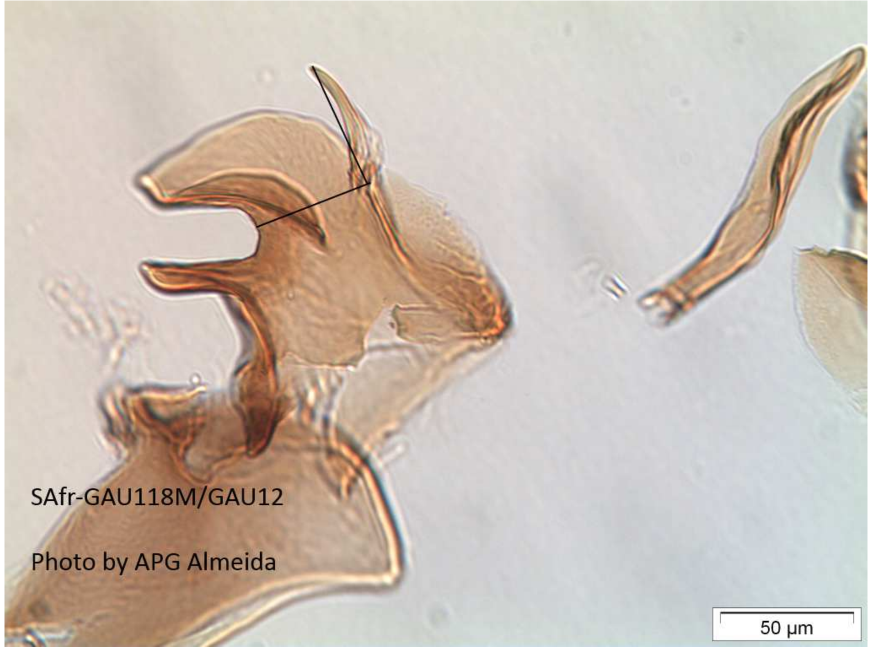

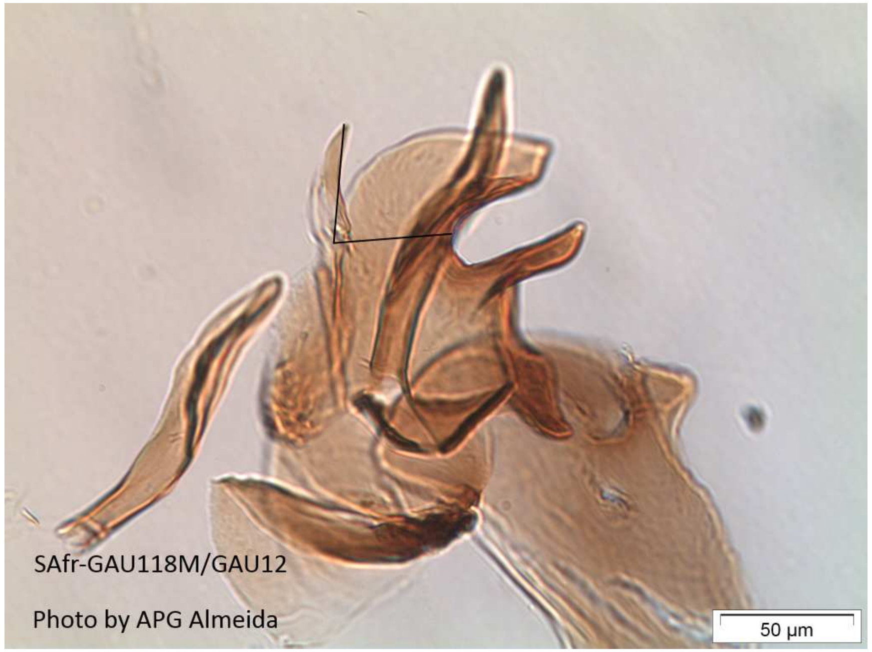

SAfr-GAU122H/GAU13; Gonocoxite, X100

- 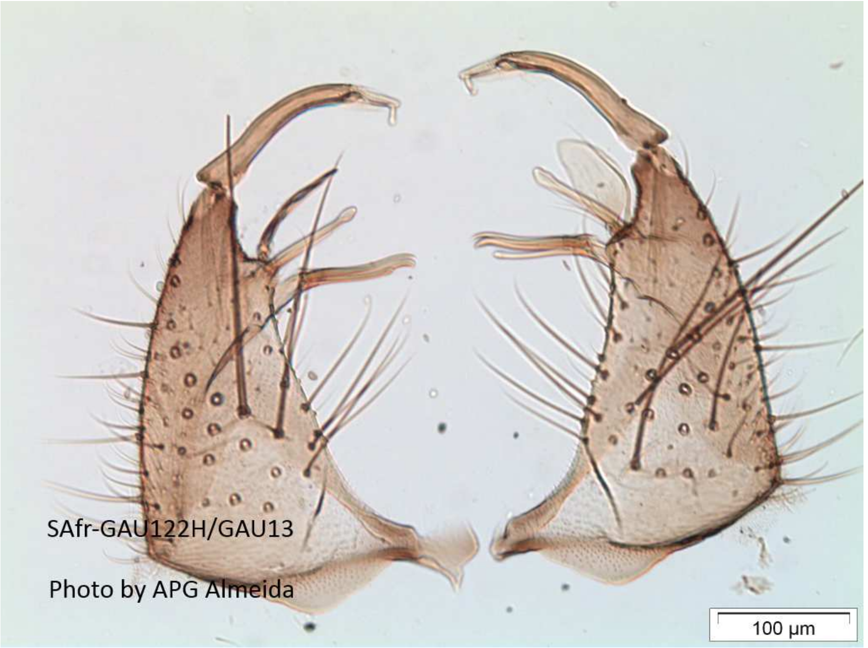

SAfr-GAU122H/GAU13; Phalosome, X200

- 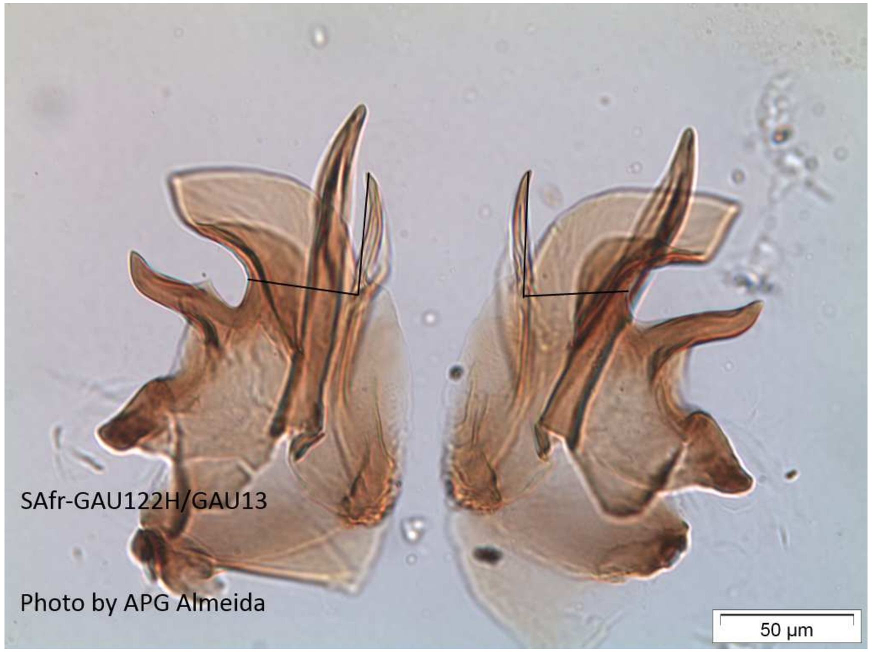

SAfr-GAU-117w1; Gonocoxite, X100

-

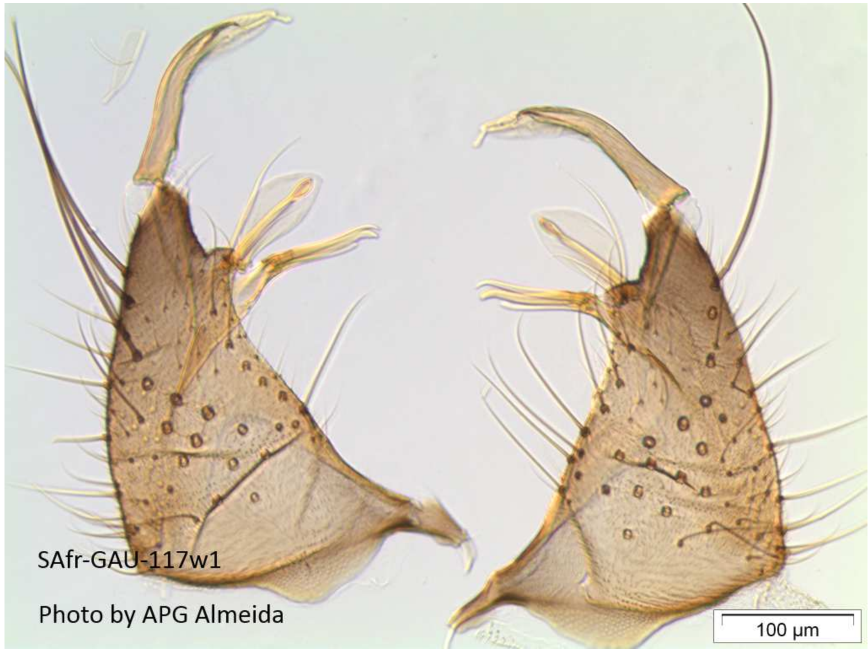

SAfr-GAU117w1; Phalosome, X200

•

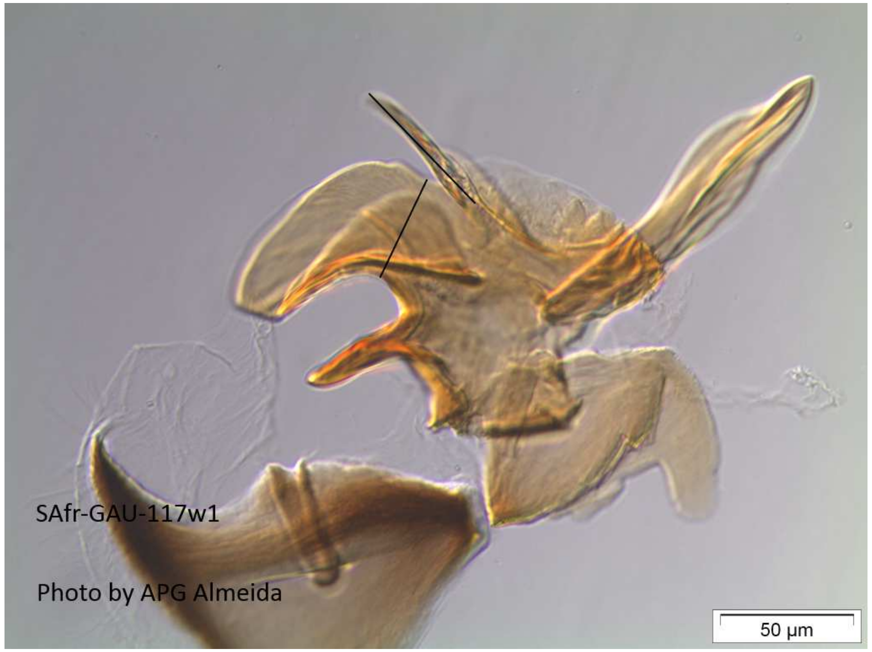

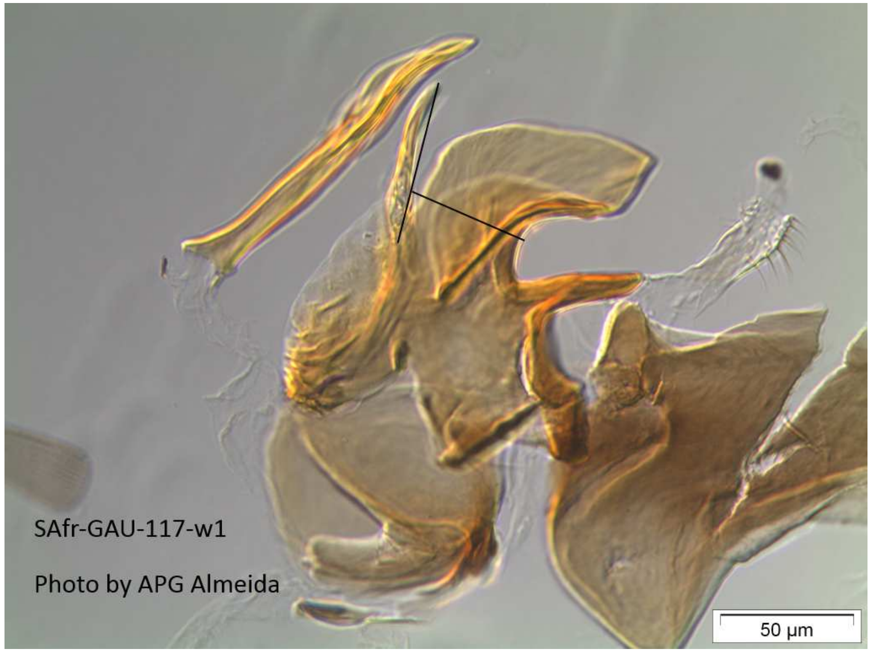

SAfr-GAU-117w2; Gonocoxite, X100

-

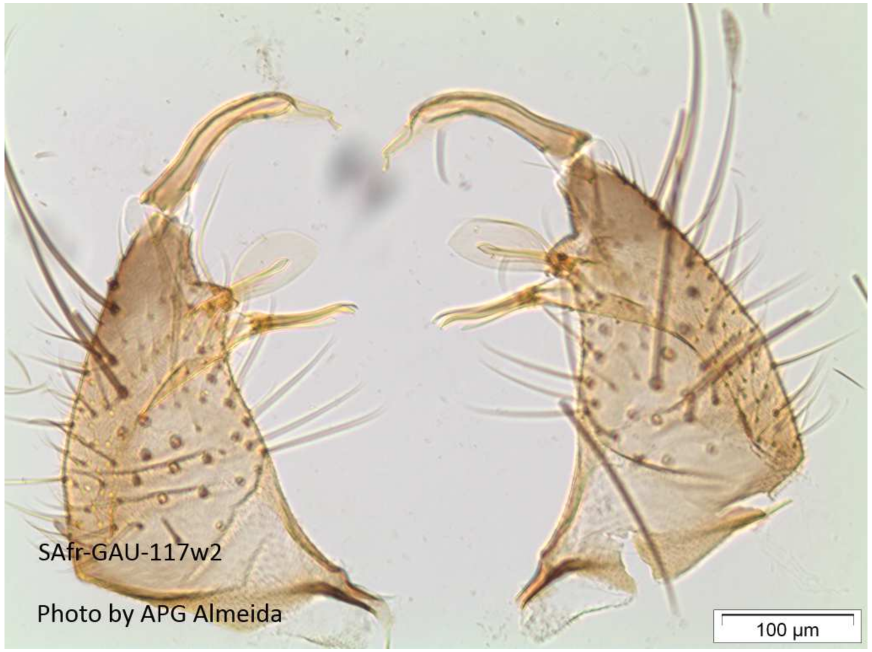

SAfr-GAU-117w2; Phalosome, X200

•

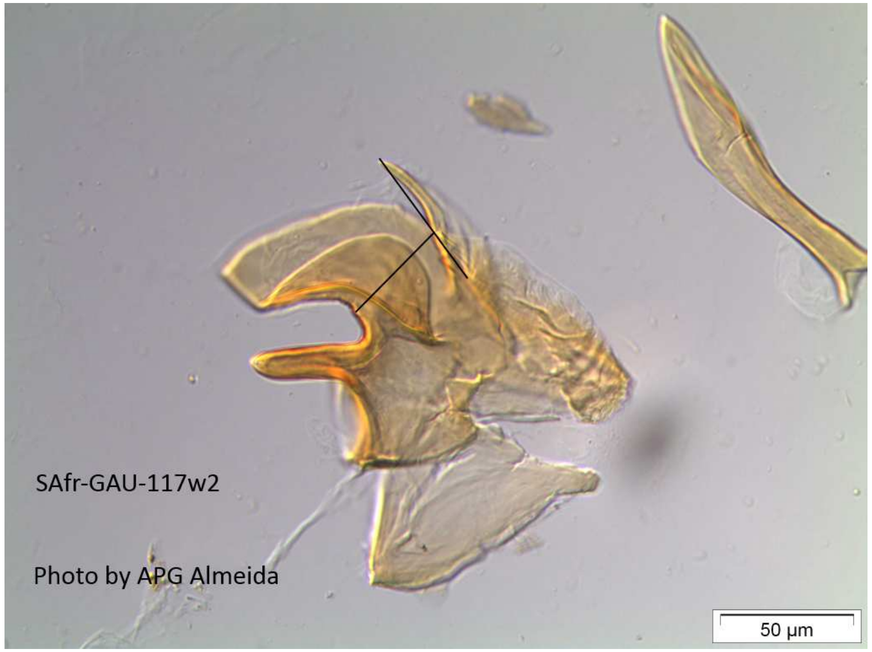

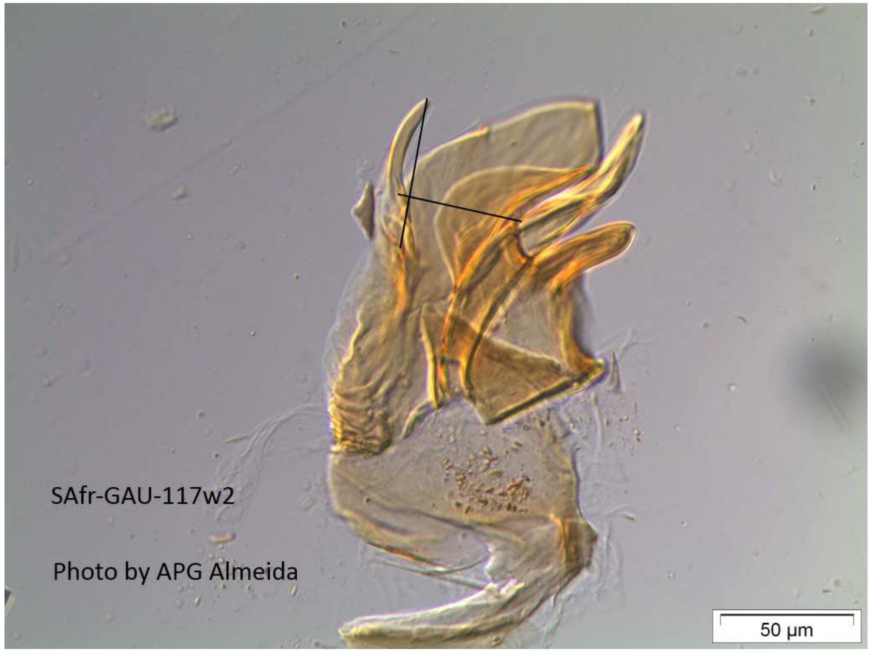

SAfr-GAU-117w3; Gonocoxite, X100

- 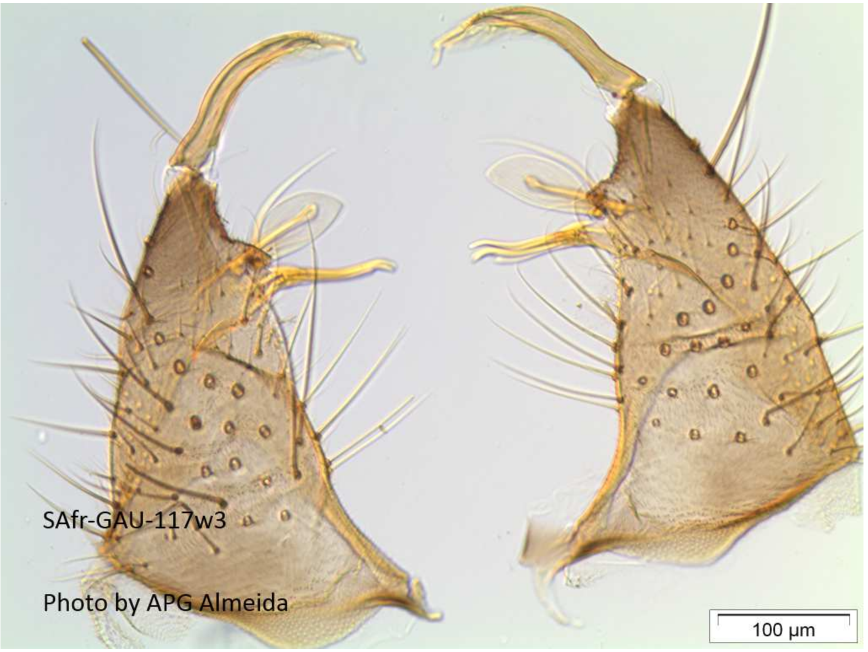

SAfr-GAU-117w3; Phalosome, X200

•

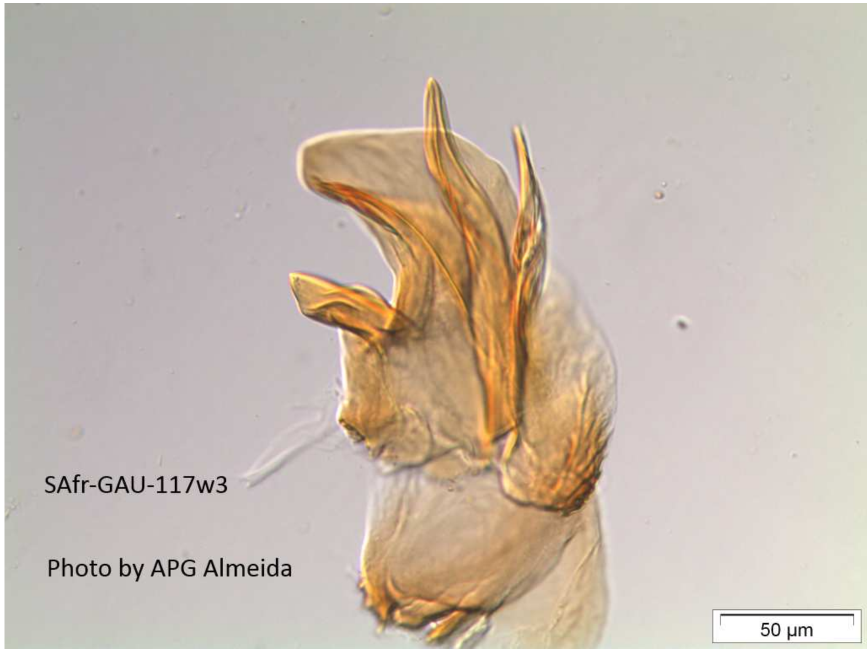

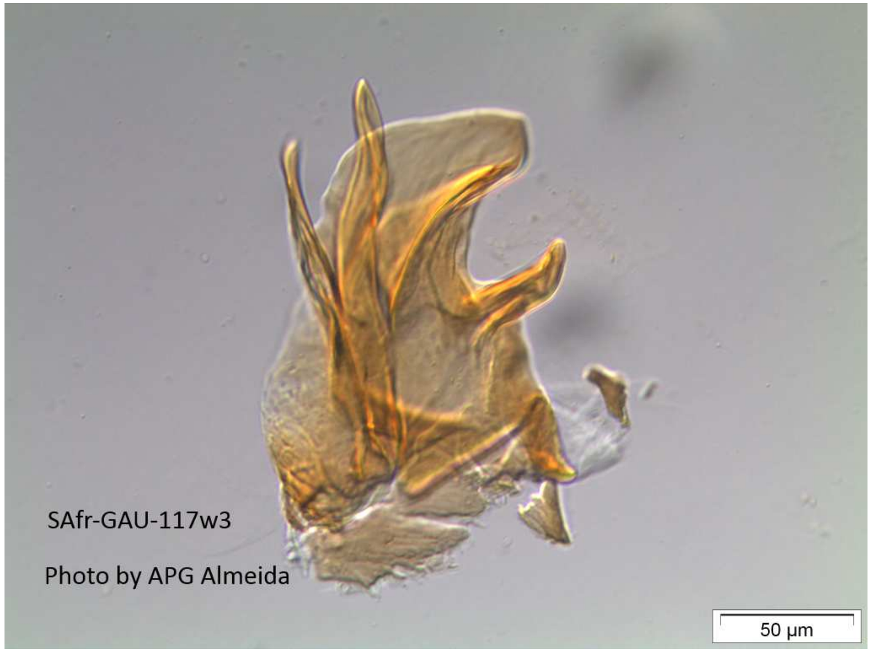

SAfr-MAR-218i; Gonocoxite, X100

- 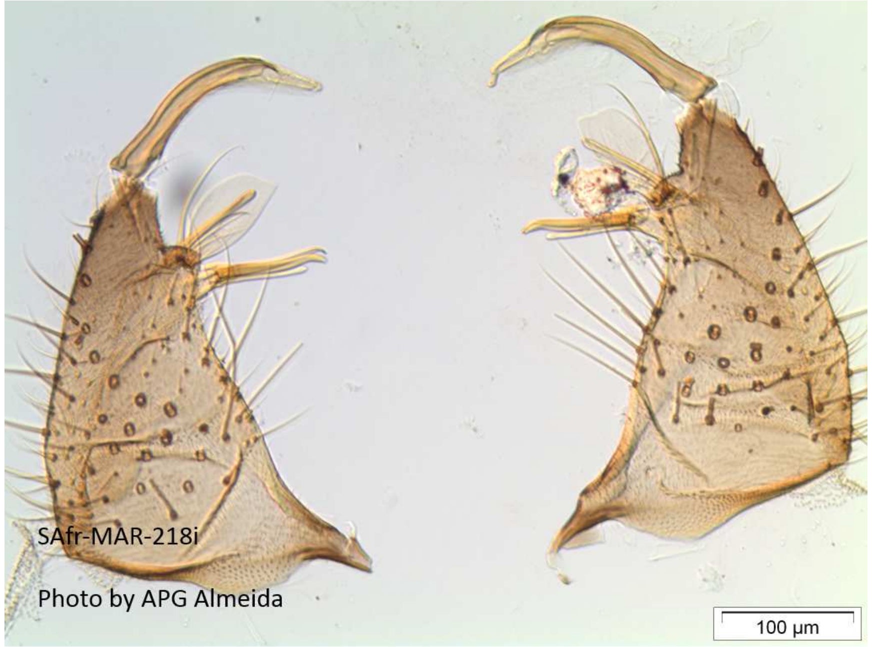

SAfr-MAR-218i; Phalosome, X200

-

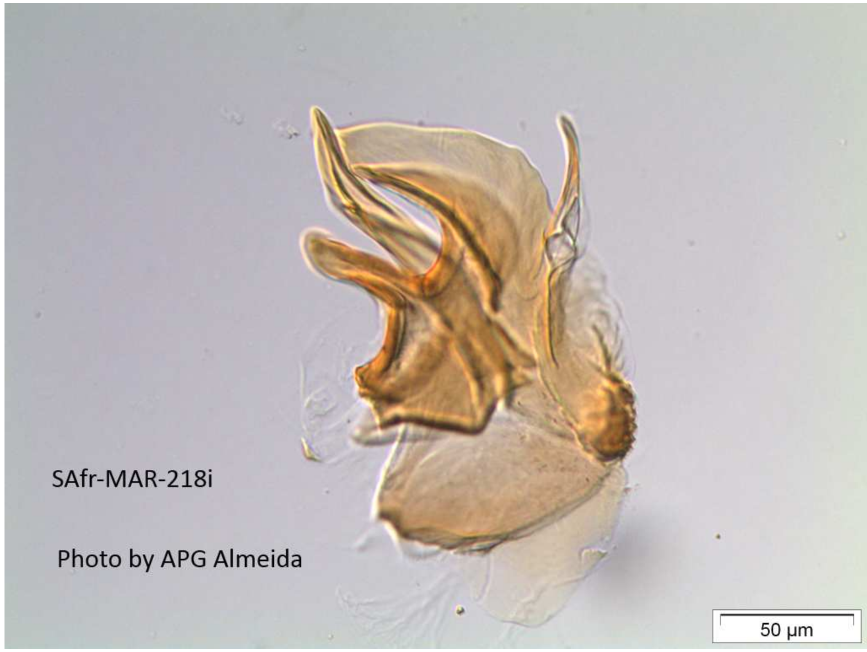

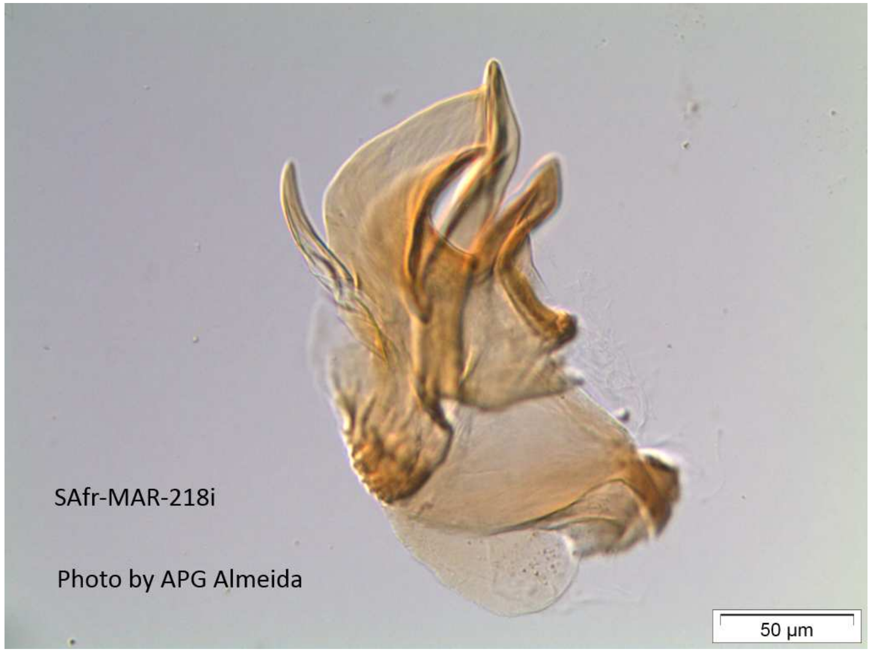

SAfr-MAR-264k; Gonocoxite, X100

- 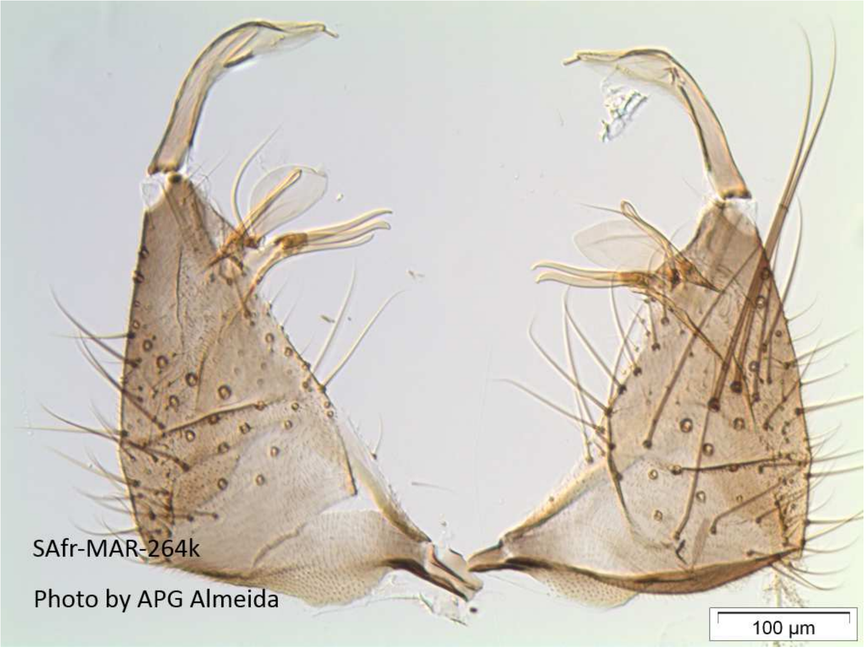

SAfr-MAR-264k; Phalosome, X200

-

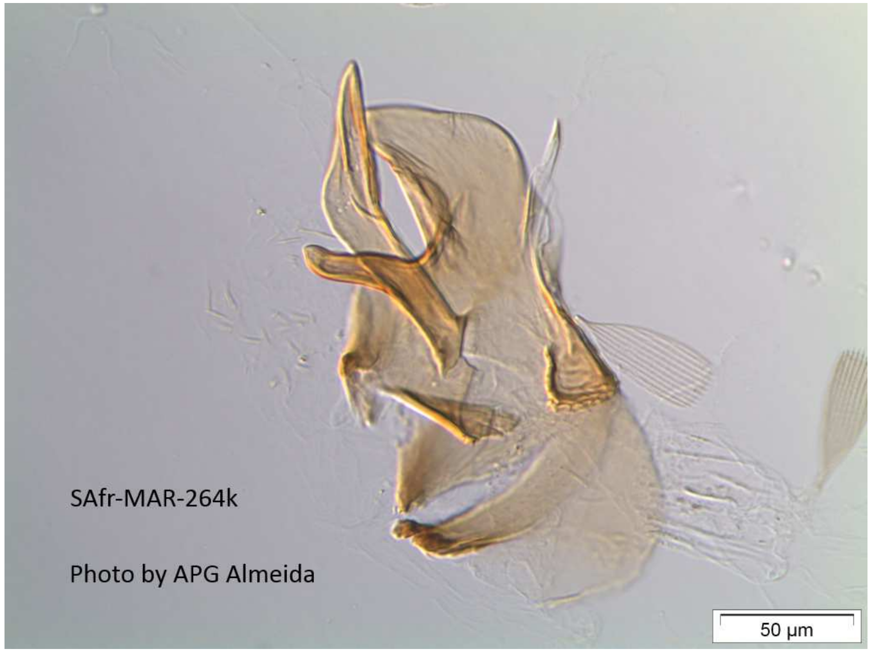

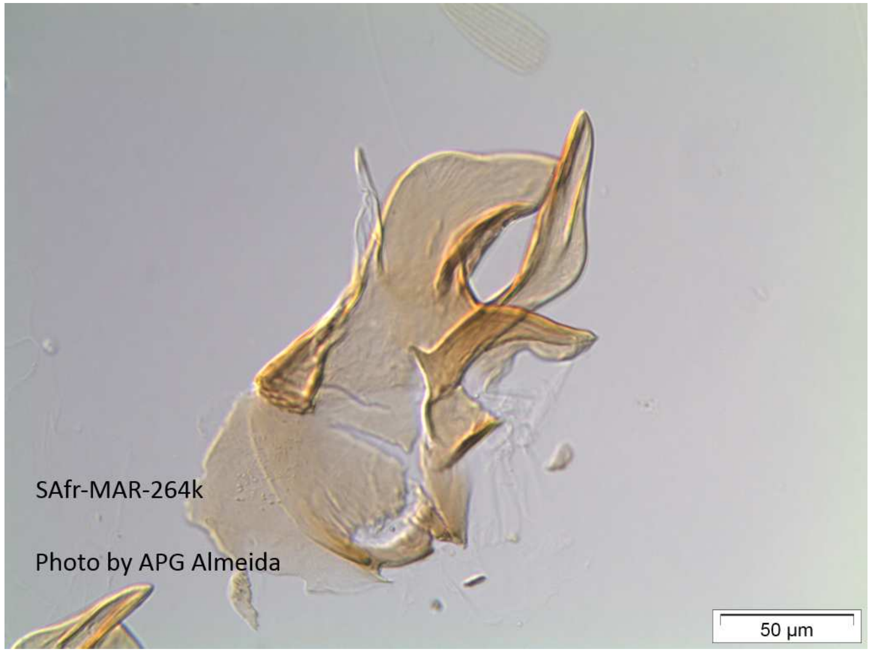

Supplement: Additional file 4: — Photos of male genitalia of Cx. univittatus from South Africa and Portuguese specimens. (PDF 3361 kb) [file 13071_2016_1877_MOESM4_ESM.pdf]
