# Supplementary material for: Comparative morphological and molecular analysis confirms the presence of the West Nile virus mosquito vector, Culex univittatus, in the Iberian Peninsula
Source: Parasit Vectors. 2016 Nov 25;9:601. doi: 10.1186/s13071-016-1877-7 (PMC5123335; doi:10.1186/s13071-016-1877-7)

BOLD Systems Identification results

Similarity

- Specimen from Iberian Peninsula (Port-256)

Specimen Identification Request

Print

Query: 256Consensus **Top Hit: Arthropoda - Diptera - Culex univittatus (98.01%)**

Search Result:

A species level match could not be made. The nearest match is with *Culex univittatus*.

A species page is available for this taxon: Species Page

Closest matching BIN (within 3%): BIN Page

For a hierarchical placement - a neighbor-joining tree is provided: Tree Based Identification

---

Identification Summary:

Taxonomic Level	Taxon Assignment	Probability of Placement (%)
Phylum	Arthropoda	100
Class	Insecta	100
Order	Diptera	100
Family	Culicidae	100
Genus	Culex	98

Similarity Scores of Top 99 Matches:

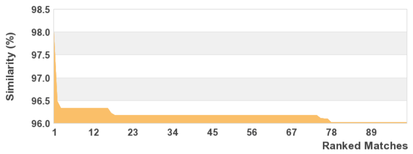

TOP 20 Matches :

Display option: Top 20

Phylum	Class	Order	Family	Genus	Species	Similarity (%)	Status
Arthropoda	Insecta	Diptera	Culicidae	Culex	*univittatus*	98.01	Early-Release
Arthropoda	Insecta	Diptera	Culicidae	Culex	*perexiguus*	96.48	Private
Arthropoda	Insecta	Diptera	Culicidae	Culex	*univittatus*	96.33	Private
Arthropoda	Insecta	Diptera	Culicidae	Culex	*univittatus*	96.33	Private
Arthropoda	Insecta	Diptera	Culicidae	Culex	*univittatus*	96.33	Private
Arthropoda	Insecta	Diptera	Culicidae	Culex	*univittatus*	96.33	Private
Arthropoda	Insecta	Diptera	Culicidae	Culex	*univittatus*	96.33	Private
Arthropoda	Insecta	Diptera	Culicidae	Culex	*univittatus*	96.33	Private
Arthropoda	Insecta	Diptera	Culicidae	Culex	*univittatus*	96.33	Private
Arthropoda	Insecta	Diptera	Culicidae	Culex	*univittatus*	96.33	Private
Arthropoda	Insecta	Diptera	Culicidae	Culex	*univittatus*	96.33	Private
Arthropoda	Insecta	Diptera	Culicidae	Culex	*univittatus*	96.33	Private
Arthropoda	Insecta	Diptera	Culicidae	Culex	*univittatus*	96.33	Private
Arthropoda	Insecta	Diptera	Culicidae	Culex	*univittatus*	96.33	Private
Arthropoda	Insecta	Diptera	Culicidae	Culex	*perexiguus*	96.33	Private
Arthropoda	Insecta	Diptera	Culicidae	Culex	*perexiguus*	96.33	Private
Arthropoda	Insecta	Diptera	Culicidae	Culex	*univittatus*	96.22	Private
Arthropoda	Insecta	Diptera	Culicidae	Culex	*perexiguus*	96.18	Private
Arthropoda	Insecta	Diptera	Culicidae	Culex	*perexiguus*	96.18	Private
Arthropoda	Insecta	Diptera	Culicidae	Culex	*perexiguus*	96.18	Private

Sampling Sites For Top Hits (>98% Match):

Similarity

➢ Specimen from Iberian Peninsula (Spai-7C5)

Specimen Identification Request

Print

Query: Spai-7C5 — Top Hit: Arthropoda - Diptera - Culex univittatus (98.11%)

Search Result:

A species level match could not be made. The nearest match is with *Culex univittatus*.

A species page is available for this taxon: Species Page

Closest matching BIN (within 3%): BIN Page

For a hierarchical placement - a neighbor-joining tree is provided: Tree Based Identification

Identification Summary:

Taxonomic Level	Taxon Assignment	Probability of Placement (%)
Phylum	Arthropoda	100
Class	Insecta	100
Order	Diptera	100
Family	Culicidae	100
Genus	Culex	98.1

Similarity Scores of Top 99 Matches:

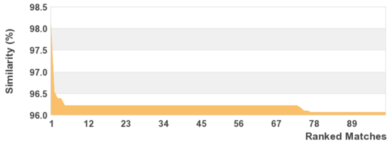

TOP 20 Matches :

Display option: Top 20

Phylum	Class	Order	Family	Genus	Species	Similarity (%)	Status
Arthropoda	Insecta	Diptera	Culicidae	Culex	*univittatus*	98.11	Early-Release
Arthropoda	Insecta	Diptera	Culicidae	Culex	*perexiguus*	96.54	Private
Arthropoda	Insecta	Diptera	Culicidae	Culex	*perexiguus*	96.38	Private
Arthropoda	Insecta	Diptera	Culicidae	Culex	*perexiguus*	96.38	Private
Arthropoda	Insecta	Diptera	Culicidae	Culex	*perexiguus*	96.23	Private
Arthropoda	Insecta	Diptera	Culicidae	Culex	*perexiguus*	96.23	Private
Arthropoda	Insecta	Diptera	Culicidae	Culex	*perexiguus*	96.23	Private
Arthropoda	Insecta	Diptera	Culicidae	Culex	*perexiguus*	96.23	Private
Arthropoda	Insecta	Diptera	Culicidae	Culex	*perexiguus*	96.23	Private
Arthropoda	Insecta	Diptera	Culicidae	Culex	*perexiguus*	96.23	Private
Arthropoda	Insecta	Diptera	Culicidae	Culex	*perexiguus*	96.23	Private
Arthropoda	Insecta	Diptera	Culicidae	Culex	*perexiguus*	96.23	Published
Arthropoda	Insecta	Diptera	Culicidae	Culex	*univittatus*	96.23	Private
Arthropoda	Insecta	Diptera	Culicidae	Culex	*univittatus*	96.23	Private
Arthropoda	Insecta	Diptera	Culicidae	Culex	*univittatus*	96.23	Private
Arthropoda	Insecta	Diptera	Culicidae	Culex	*univittatus*	96.23	Private
Arthropoda	Insecta	Diptera	Culicidae	Culex	*univittatus*	96.23	Private
Arthropoda	Insecta	Diptera	Culicidae	Culex	*univittatus*	96.23	Private
Arthropoda	Insecta	Diptera	Culicidae	Culex	*univittatus*	96.23	Private
Arthropoda	Insecta	Diptera	Culicidae	Culex	*univittatus*	96.23	Private

Sampling Sites For Top Hits (>98% Match):

Similarity

➢ Specimen from South Africa (GAU1)

Specimen Identification Request

Print

Query: GAU1Consensus — **Top Hit: Arthropoda - Diptera - Culex univittatus (99.54%)**

Search Result:

The submitted sequence has been matched to *Culex univittatus*. This identification is solid unless there is a very closely allied congeneric species that has not yet been analyzed. Such cases are rare.

A species page is available for this taxon: Species Page

Closest matching BIN (within 3%): BIN Page

For a hierarchical placement - a neighbor-joining tree is provided: Tree Based Identification

---

Identification Summary:

Taxonomic Level	Taxon Assignment	Probability of Placement (%)
Phylum	Arthropoda	100
Class	Insecta	100
Order	Diptera	100
Family	Culicidae	100
Genus	Culex	100
Species	Culex univittatus	99.5

Similarity Scores of Top 99 Matches:

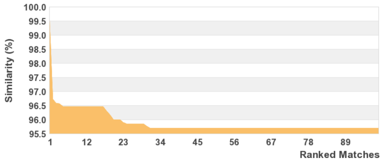

TOP 20 Matches :

Display option: Top 20

Phylum	Class	Order	Family	Genus	Species	Similarity (%)	Status
Arthropoda	Insecta	Diptera	Culicidae	Culex	*univittatus*	99.54	Early-Release
Arthropoda	Insecta	Diptera	Culicidae	Culex	*univittatus*	96.74	Private
Arthropoda	Insecta	Diptera	Culicidae	Culex	*sp. KHH5*	96.58	Early-Release
Arthropoda	Insecta	Diptera	Culicidae	Culex	*univittatus*	96.57	Private
Arthropoda	Insecta	Diptera	Culicidae	Culex	*univittatus*	96.47	Private
Arthropoda	Insecta	Diptera	Culicidae	Culex	*univittatus*	96.47	Private
Arthropoda	Insecta	Diptera	Culicidae	Culex	*univittatus*	96.47	Private
Arthropoda	Insecta	Diptera	Culicidae	Culex	*univittatus*	96.47	Private
Arthropoda	Insecta	Diptera	Culicidae	Culex	*univittatus*	96.47	Private
Arthropoda	Insecta	Diptera	Culicidae	Culex	*univittatus*	96.47	Private
Arthropoda	Insecta	Diptera	Culicidae	Culex	*univittatus*	96.47	Private
Arthropoda	Insecta	Diptera	Culicidae	Culex	*univittatus*	96.47	Private
Arthropoda	Insecta	Diptera	Culicidae	Culex	*univittatus*	96.47	Private
Arthropoda	Insecta	Diptera	Culicidae	Culex	*univittatus*	96.47	Private
Arthropoda	Insecta	Diptera	Culicidae	Culex	*univittatus*	96.47	Private
Arthropoda	Insecta	Diptera	Culicidae	Culex	*univittatus*	96.47	Private
Arthropoda	Insecta	Diptera	Culicidae	Culex	*univittatus*	96.47	Private
Arthropoda	Insecta	Diptera	Culicidae	Culex	*univittatus*	96.31	Private
Arthropoda	Insecta	Diptera	Culicidae	Culex	*univittatus*	96.17	Private
Arthropoda	Insecta	Diptera	Culicidae	Culex	*perexiguus*	96.01	Private

Sampling Sites For Top Hits (>98% Match):

Similarity

- Specimen from Turkey (accession number: KJ012109.1)

Specimen Identification Request

Print

Query: gi|586946611|gb|KJ012109.1| — Top Hit: Arthropoda - Diptera - Culex perexiguus (100%)

Search Result:

The submitted sequence has been matched to *Culex perexiguus*. This identification is solid unless there is a very closely allied congeneric species that has not yet been analyzed. Such cases are rare.

A species page is available for this taxon: Species Page

Closest matching BIN (within 3%): BIN Page

For a hierarchical placement - a neighbor-joining tree is provided: Tree Based Identification

Identification Summary:

Taxonomic Level	Taxon Assignment	Probability of Placement (%)
Phylum	Arthropoda	100
Class	Insecta	100
Order	Diptera	100
Family	Culicidae	100
Genus	Culex	100
Species	Culex perexiguus	100

Similarity Scores of Top 99 Matches:

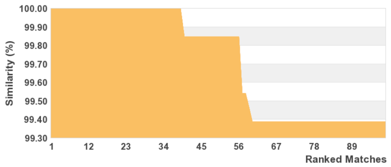

TOP 20 Matches :

Display option: Top 20

Phylum	Class	Order	Family	Genus	Species	Similarity (%)	Status
Arthropoda	Insecta	Diptera	Culicidae	Culex	*perexiguus*	100	Private
Arthropoda	Insecta	Diptera	Culicidae	Culex	*perexiguus*	100	Private
Arthropoda	Insecta	Diptera	Culicidae	Culex	*perexiguus*	100	Private
Arthropoda	Insecta	Diptera	Culicidae	Culex	*perexiguus*	100	Private
Arthropoda	Insecta	Diptera	Culicidae	Culex	*perexiguus*	100	Private
Arthropoda	Insecta	Diptera	Culicidae	Culex	*perexiguus*	100	Private
Arthropoda	Insecta	Diptera	Culicidae	Culex	*perexiguus*	100	Private
Arthropoda	Insecta	Diptera	Culicidae	Culex	*perexiguus*	100	Private
Arthropoda	Insecta	Diptera	Culicidae	Culex	*perexiguus*	100	Private
Arthropoda	Insecta	Diptera	Culicidae	Culex	*perexiguus*	100	Private
Arthropoda	Insecta	Diptera	Culicidae	Culex	*perexiguus*	100	Private
Arthropoda	Insecta	Diptera	Culicidae	Culex	*perexiguus*	100	Private
Arthropoda	Insecta	Diptera	Culicidae	Culex	*perexiguus*	100	Private
Arthropoda	Insecta	Diptera	Culicidae	Culex	*perexiguus*	100	Private
Arthropoda	Insecta	Diptera	Culicidae	Culex	*perexiguus*	100	Private
Arthropoda	Insecta	Diptera	Culicidae	Culex	*perexiguus*	100	Private
Arthropoda	Insecta	Diptera	Culicidae	Culex	*perexiguus*	100	Private
Arthropoda	Insecta	Diptera	Culicidae	Culex	*perexiguus*	100	Private
Arthropoda	Insecta	Diptera	Culicidae	Culex	*perexiguus*	100	Private
Arthropoda	Insecta	Diptera	Culicidae	Culex	*perexiguus*	100	Private

Sampling Sites For Top Hits (>98% Match):

Phylogenetic tree

➢ Specimen from Iberian Peninsula (Port-256)

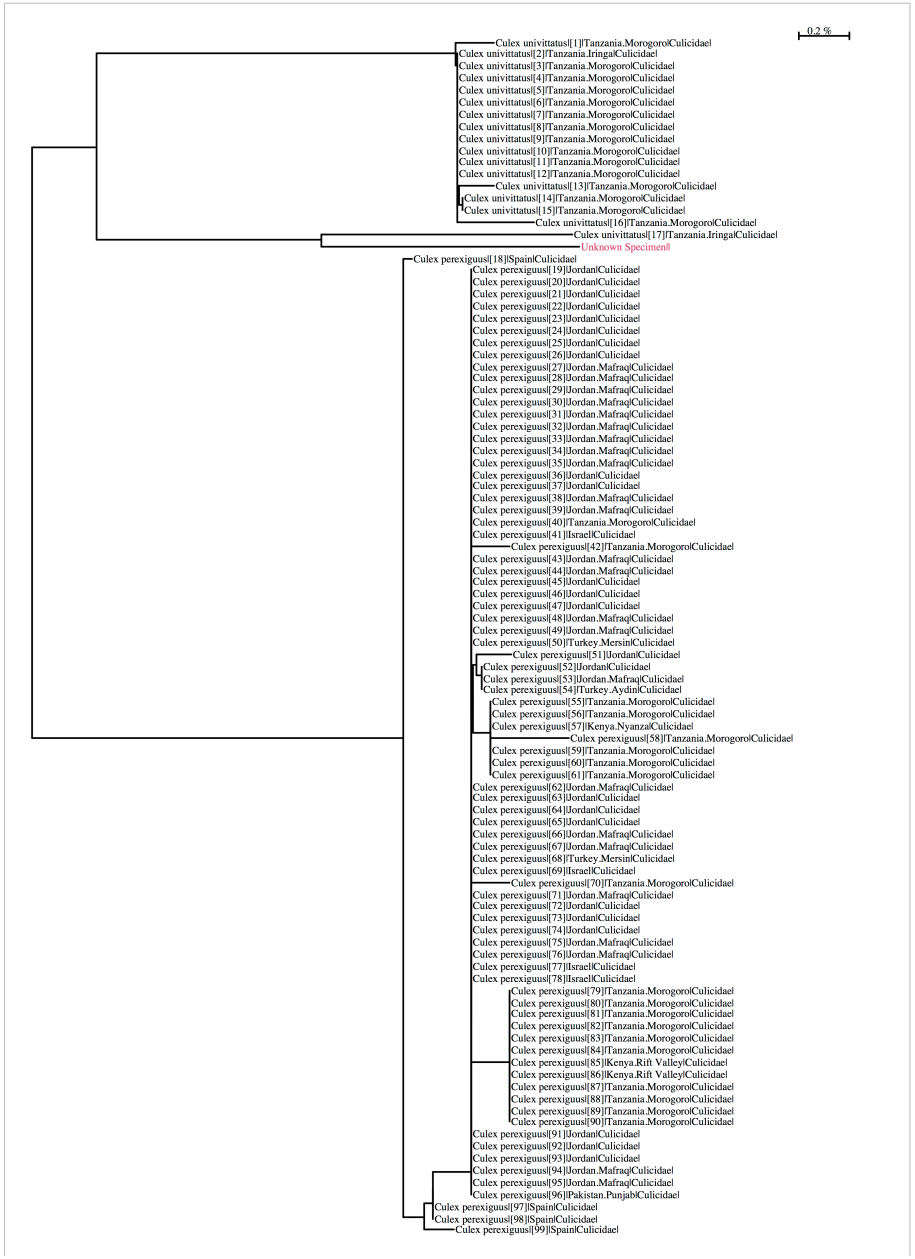

Phylogenetic tree

- Specimen from Iberian Peninsula (Spai-7C5)

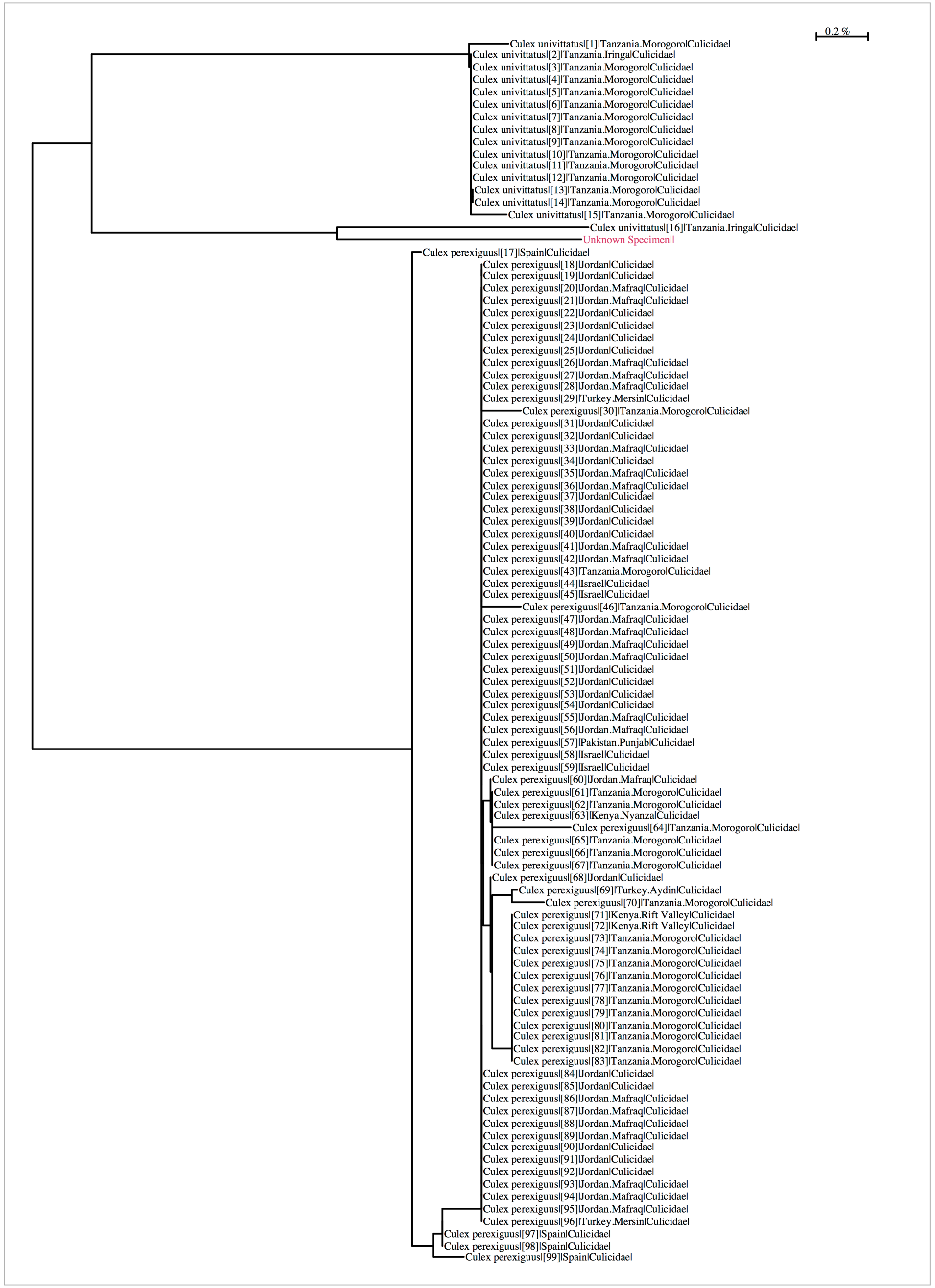

Phylogenetic tree

- Specimen from South Africa (GAU1)

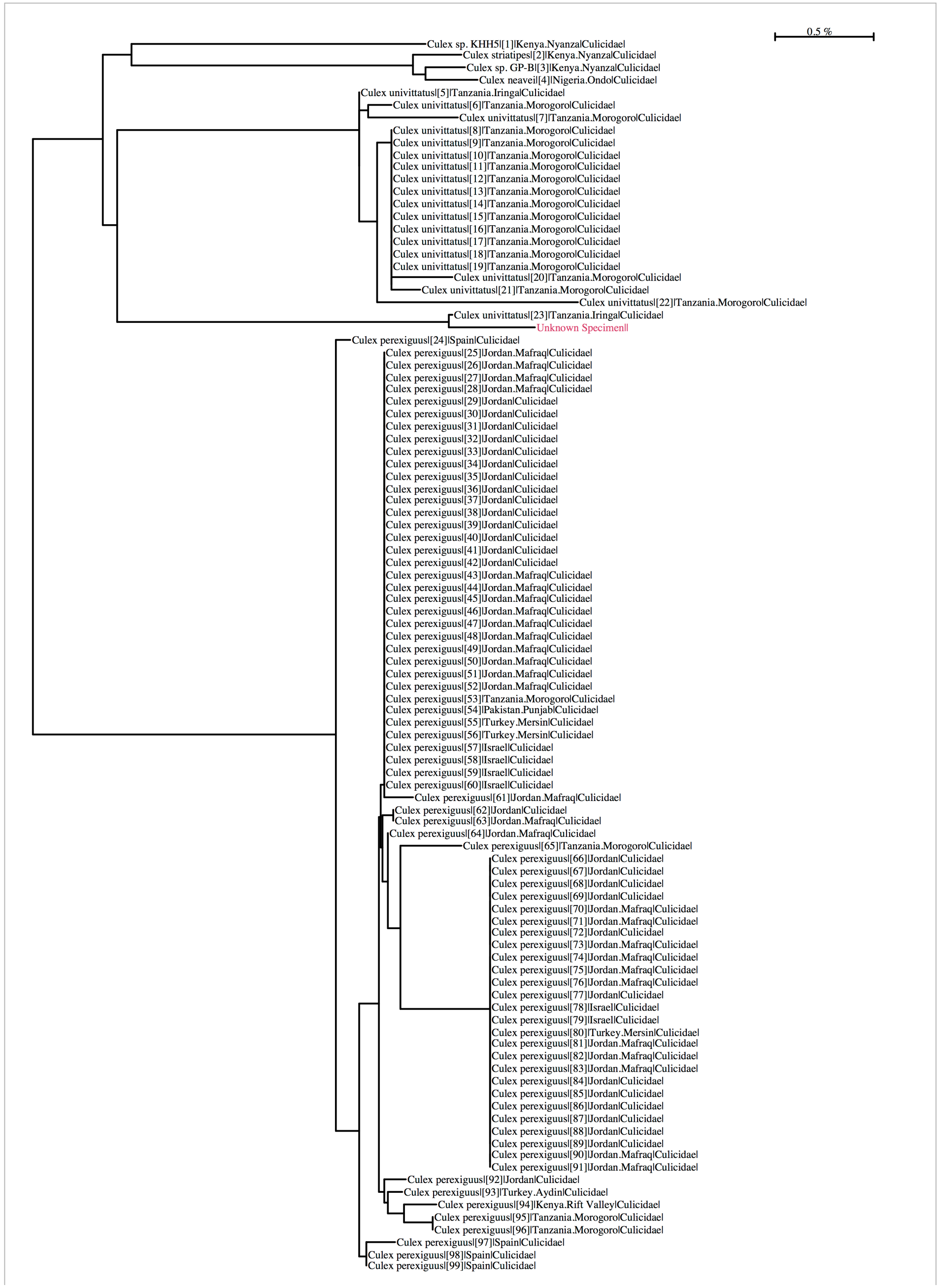

Phylogenetic tree

- Specimen from Turkey (access number: KJ012109.1)

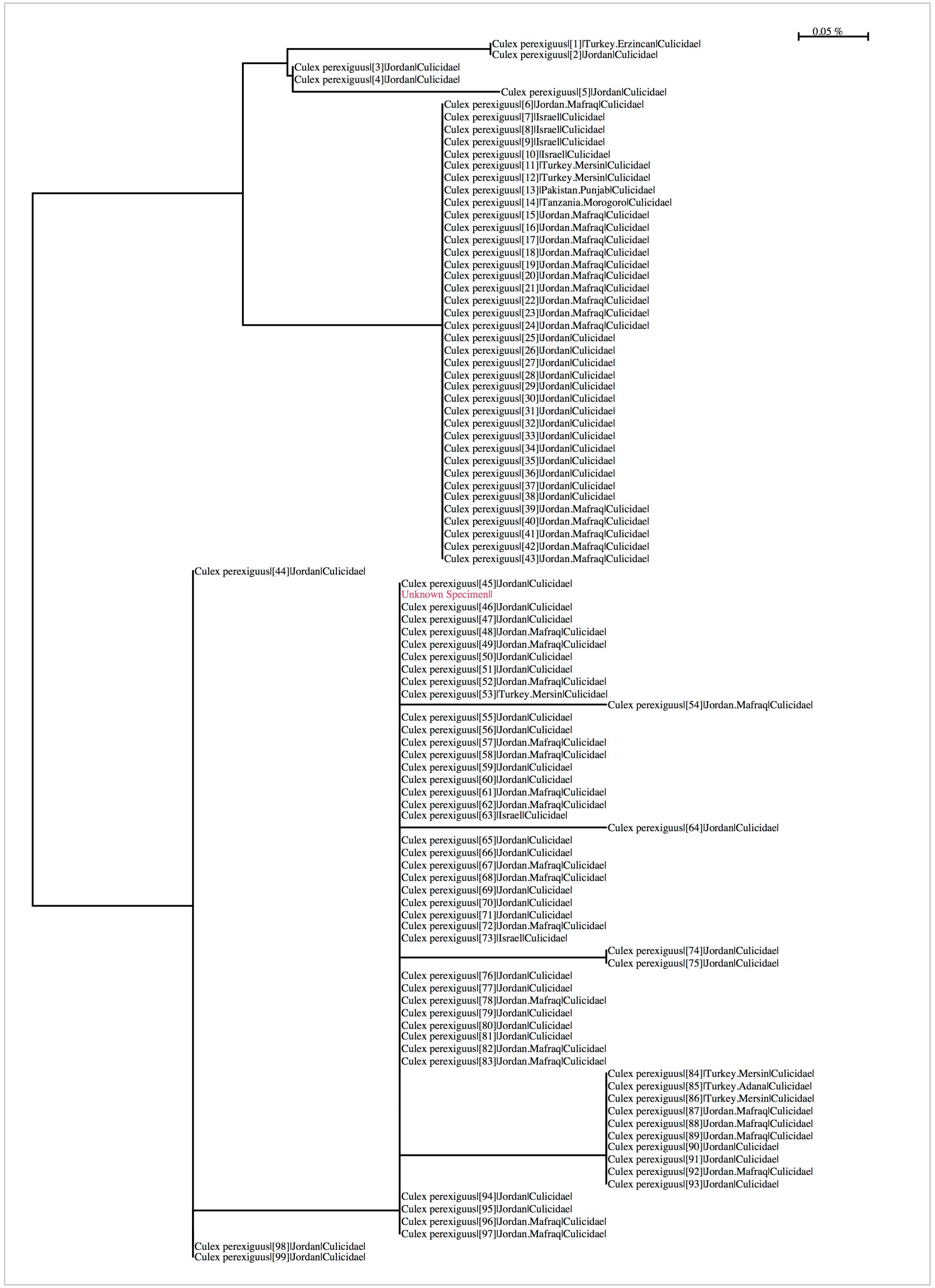

Supplement: Additional file 6: — Example of some results obtained in BOLD Systems identification tool. Percentage of similarity results and phylogenetic trees are shown. (PDF 10649 kb) [file 13071_2016_1877_MOESM6_ESM.pdf]
