# Supplementary material for: Comparative morphological and molecular analysis confirms the presence of the West Nile virus mosquito vector, Culex univittatus, in the Iberian Peninsula
Source: Parasit Vectors. 2016 Nov 25;9:601. doi: 10.1186/s13071-016-1877-7 (PMC5123335; doi:10.1186/s13071-016-1877-7)
Supplement: Additional file 7: — Variable sites found in a 637 bp region of cox1 mtDNA alignment of Univittatus subgroup. Abbreviations: SAfr, Culex univittatus from South Africa; Port, specimens from Portugal; Spai, specimens from Spain. (PDF 86 kb) [file 13071_2016_1877_MOESM7_ESM.pdf]

Additional file 7: Variable sites found in a 637 bp region of *cox*1 mtDNA alignment of Univittatus subgroup. Abbreviations: SAfr, Culex univittatus from South Africa; Port, specimens from Portugal; Spai, specimens from Spain.

		1 1 1 1 1 1 1 2 2 2 2 2 2 2 2 2 2 2 2 2 3 3 3 3 3 3 3 3 3 3 3 3 3 3 4 4 4 4 4 4 4 4 4 4 4 4 4 5 5 5 5 5 5 5 5 5 5 5 5 5 5 5 5 6 6 6 6 6
		2 4 4 5 7 8 9 0 0 1 1 2 4 4 0 1 1 2 2 2 3 3 7 7 8 8 9 0 0 0 1 2 2 3 5 6 6 7 7 7 8 1 1 3 5 6 7 7 8 8 8 9 9 9 0 1 2 2 3 3 3 4 4 5 5 6 6 6 7 8 0 1 3 3 3
**Sequence**	**Group**	1 2 3 9 5 9 5 1 0 6 2 8 1 2 8 5 1 7 0 6 9 2 8 1 8 6 9 8 0 2 4 6 2 8 4 5 4 7 0 3 6 2 2 5 3 1 3 5 8 4 5 7 0 6 9 8 7 0 6 0 6 8 2 7 3 6 2 5 8 8 9 7 9 1 4 7
Port-256	Portugal and Spain	A A A T A C T A A T T C T T A T G A C C G T C C T A G T G A A A T A T T C T T T T T T A A A T A T A C T T G C A T A T G T A C C T T T A T C C A A A C T
Port-276	Portugal and Spain	. . . .
Port-3435	Portugal and Spain	. . . .
Port-260	Portugal and Spain	. . . .
Port-738	Portugal and Spain	. . . .
Port-280	Portugal and Spain	. . . .
Port-521	Portugal and Spain	. . . .
Port-266	Portugal and Spain	. . . .
Port-527	Portugal and Spain	. . . .
Port-C13	Portugal and Spain	. . . .
Port-C14	Portugal and Spain	. . . .
Port-653	Portugal and Spain	. . . .
Port-C4	Portugal and Spain	. . . .
Port-C15	Portugal and Spain	. . . . C . . . .
Port-270	Portugal and Spain	. . . . G . . . .
Port-2467	Portugal and Spain	. . . . G . . . .
Spai-3X2	Portugal and Spain	. . . . G . . . .
Spai-6B	Portugal and Spain	. . . .
Spai-7C4	Portugal and Spain	. . . . A . . . .
Spai-3X1	Portugal and Spain	. . . .
Spai-7C1	Portugal and Spain	. . . .
Spai-11E3	Portugal and Spain	. . . .
Spai-13X	Portugal and Spain	. . . .
Spai-11A3	Portugal and Spain	. . . .
Spai-3X4	Portugal and Spain	. . . .
Spai-7C3	Portugal and Spain	. . . .
Spai-3X3	Portugal and Spain	. . . . A . . . .
Spai-67.2A	Portugal and Spain	. . . . C . . . .
Spai-7C2	Portugal and Spain	. . . .
Spai-7C5	Portugal and Spain	. . . .
Spai-1X	Portugal and Spain	. . . .
SAfr-GAU2	*Cx. univittatus*	G . . . . T . . . C . . . C . . A . T . . . . T . . A . . . . C . . . . . . C . . . . A . . . . T A . . . G . . . .
SAfr-GAU13	*Cx. univittatus*	G . . . . T . . . C . . . C . . A . T . . . . T . . A . . . . C . . . . . . C . . . . A . . . . T A . . . .
SAfr-GAU5	*Cx. univittatus*	. . G . . T . . . C . . . . . . A G . . . . . T . . A . . . . G . . . C . . . . G . . . . A . . . . C . . . T A . . . .
SAfr-GAU1	*Cx. univittatus*	G . . . . T . . G C . . . C . . A . T . . . . T . . A . . . . C . . . . A . . . . T A . . . . T .
SAfr-GAU3	*Cx. univittatus*	. . G . . T . . G C . . . . . . A G . . . . . T . . A . . . . G . . . C . . . C . . . . G . . . . A . . . . C . . . T A . . . .
SAfr-GAU9	*Cx. univittatus*	. . G . . T . . G C C . . . . . A G . . . . . T . . A . . . . G . . . C . . . . G . . . . A . . . . C . . . T A . . . .
SAfr-GAU7	*Cx. univittatus*	. . . . . T . . G C . . . C . . A . T . . . . T . . A . . . . C . . . . A . . . . T A . . . .
SAfr-GAU10	*Cx. univittatus*	. . . . . T . . G C . . . C . . A . T . . . . T . . A . . . . C . . . . A . . . . T A . . . . T .
SAfr-GAU4	*Cx. univittatus*	. G . . . T . . G C . . . C . . A . T . . . . T . . A . . . . C . . . . A . . . . T A . . . . C . . T .
SAfr-GAU6	*Cx. univittatus*	. . . . . T . . G C . . . C . . A . T . . . . T . . A . . . . C . . . . A . . . . T A . . . .
SAfr-GAU8	*Cx. univittatus*	. . . . . T . . G C . . . C . . A . T . . . . T . . A . . . . C . . . . A . . . . T A . . . .
SAfr-GAU12	*Cx. univittatus*	. . . . . T . . G C . . . C . . A . T . . . . T . . A . . . . C . . . . A . . . . T A . . . . T .
SAfr-GAU11	*Cx. univittatus*	. . . . . T . . G C . . . C . . A . T . . . . T . . A . . . . C . . . . A . . . . T A . . . . T .
KJ012109.1| Cx perexiguus	*Cx. perexiguus*	. . . . . T . . G . . T . . . A A . . T A . T T . G . . . . A A . C G . T . T . . . . C A T . . T . . . . T T A . . . . T T . T . . .
KJ012107.1| Cx perexiguus	*Cx. perexiguus*	. . . . . T . . G . . T . . . A A . . T A . T T . G . C . . . . A A . C G . T . T . . . . C A T . . T . . . . T T A . . . . T T . T . . .
KJ012106.1| Cx perexiguus	*Cx. perexiguus*	. . . . . T . . G . . T . . . A A . . T A . T T . G . C . . . . A A . C G . T . T . . . . C A T . . T . . . . T T A . . . . T T . T . . .
KJ012103.1| Cx perexiguus	*Cx. perexiguus*	. . . . . T . . G . . T . . . A A . . T A . T T . G . C . . . . A A . C G . T . T . . . . C A T . . T . . . . T T A . . . . T T . T . . .
KJ012108.1| Cx perexiguus	*Cx. perexiguus*	. . . . . T . . . . . T . . . A A . . T A . T T . . . . A A . C G . T . T . . . . C A T . . T . . . . T T A . C . . T T . . . . .
KF406802.1| Cx perexiguus	*Cx. perexiguus*	. . . . . T . . . . . T . . . A A . . T A . T T . . . . A A . C G . T . T . . . . C A T . . T . . . . T T A . C . . T T . . . . .
KJ012104.1| Cx perexiguus	*Cx. perexiguus*	. . . . . T . . . . . T . . . A A . . T A . T T . . . . A A . C G . T . T . . . . C A T . . T . . . . T T A . C . . T T . . . . .
KJ012105.1| Cx perexiguus	*Cx. perexiguus*	. . . . . T . . . . . T . . . A A . . T A . T T . . . . A A . C G . T . T . . . . C A T . . T . . . . T T A . C . . T T . . . . .
HQ398886| Cx fuscocephala	*Cx. fuscocephala*	. G T A T T . T . . . T A . . . . . T T A A T A C . A . . T . . A . A . T C C A A A . G T . . . G T T A . A A T A T . . C T T T . A C . C T T . . G . A
KF406799| Cx fuscocephala	*Cx. fuscocephala*	. G T A T T C T . . . T A . G . . . T T A A T A C . A . . T . . A . A . T . C A A A . G T . . . A T T A . A A T A T . . C T T T . A C . . T T . . . . A
KF406798| Cx fuscocephala	*Cx. fuscocephala*	. G T A T T C T . . . T A . G . . . T T A A T A C . A . . T . . A G A . T . C A A A . G T . . . A T T A . A A T A T . . C T T T . A C . . T T . . . . A
KF406797| Cx fuscocephala	*Cx. fuscocephala*	. G T A T T C T . . . T A . . . . . T T A A T G C . A . . T . . A . A . T . C G A A . G T . . . A T T A . A A T A T . . C T T T . A C . . T T . . . . A
