# Supplementary figures and images for: Comparative morphological and molecular analysis confirms the presence of the West Nile virus mosquito vector, Culex univittatus, in the Iberian Peninsula
Source: Parasit Vectors. 2016 Nov 25;9:601. doi: 10.1186/s13071-016-1877-7 (PMC5123335; doi:10.1186/s13071-016-1877-7)

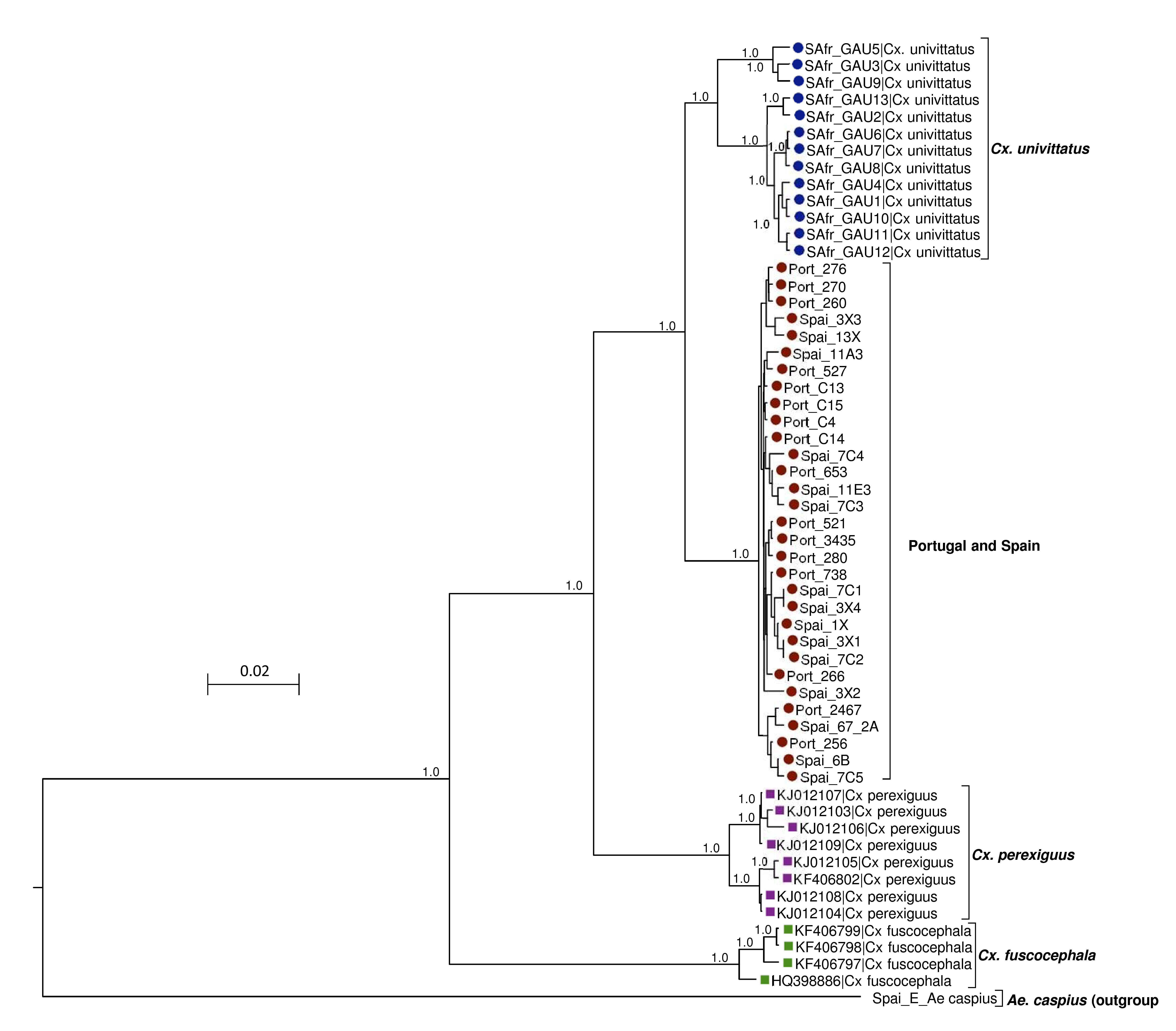

Supplement: Additional file 8: — Bayesian phylogenetic analysis (consensus tree) based on cox1 mosquito sequences. At specific branch nodes posterior probabilities ≥ 0.90 are indicated. The scale-bar indicates the number of nucleotide substitutions per site. The tree was rooted with a cox1 sequence from Ae. (Och.) caspius. (TIF 2231 kb) [file 13071_2016_1877_MOESM8_ESM.tif]

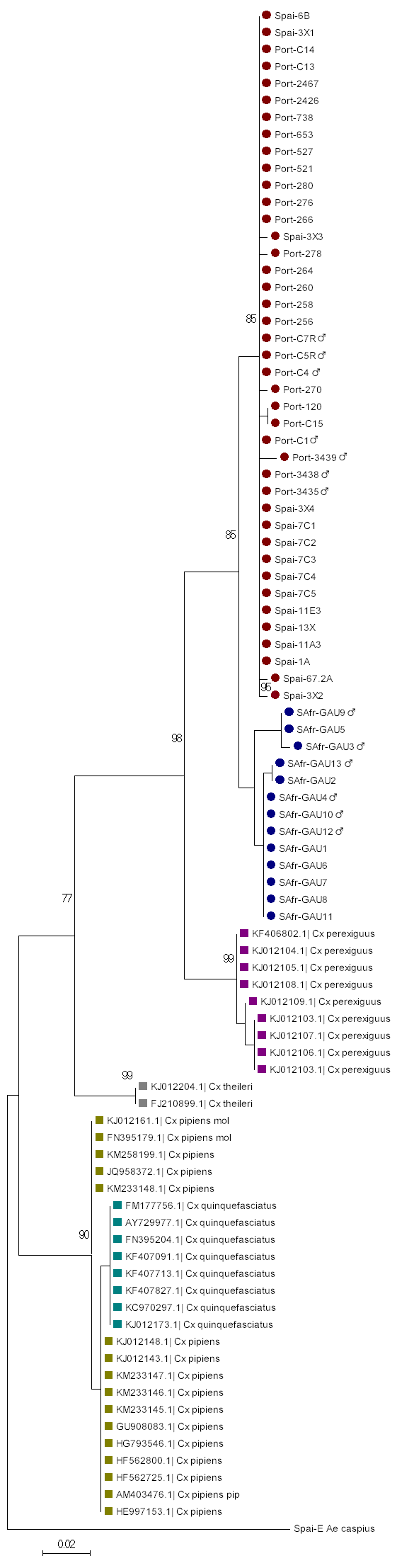

Supplement: Additional file 9: — Molecular phylogenetic analysis of a small fragment of the cox1 alignment, with a higher number of male sequences, by Maximum Likelihood. The tree with the highest log likelihood (-883.6486) is shown. The percentage of trees in which the associated taxa clustered together is shown next to the branches. The scale-bar indicates 0.02 substitutions per site. The analysis involved 90 nucleotide sequences. There were a total of 287 positions in the final dataset. (TIFF 240 kb) [file 13071_2016_1877_MOESM9_ESM.tiff]
