# Supplementary material for: Comparative morphological and molecular analysis confirms the presence of the West Nile virus mosquito vector, Culex univittatus, in the Iberian Peninsula
Source: Parasit Vectors. 2016 Nov 25;9:601. doi: 10.1186/s13071-016-1877-7 (PMC5123335; doi:10.1186/s13071-016-1877-7)
Supplement: Additional file 10: — Estimates of average evolutionary divergence in cox1 over sequence pairs within groups. The numbers of base substitutions per site from averaging over all sequence pairs within each group are shown. Standard error estimates are shown in the last column. The analysis involved 84 nucleotide sequences. Codon positions included were 1st + 2nd + 3rd + Noncoding. All positions containing gaps and missing data were eliminated. There were a total of 637 positions in the final dataset. (PDF 59 kb) [file 13071_2016_1877_MOESM10_ESM.pdf]

Additional file 10: Estimates of average evolutionary divergence in *cox*1 over sequence pairs within groups. The numbers of base substitutions per site from averaging over all sequence pairs within each group are shown. Standard error estimates are shown in the last column. The analysis involved 84 nucleotide sequences. Codon positions included were 1st+2nd+3rd+Noncoding. All positions containing gaps and missing data were eliminated. There were a total of 637 positions in the final dataset

Group	Number of base substitutions per site from averaging over all sequence pairs within each group	SE
Portugal and Spain	0,001	0,000
Cx. univittatus	0,008	0,002
Cx. perexiguus	0,004	0,002
Cx. fuscocephala	0,007	0,002
Cx. pipiens	0,001	0,001
Cx. quinquefasciatus	0,000	0,000
Cx. torrentium	0,001	0,001
Cx. theileri	0,002	0,001
